# Bridging integrator 1 protein loss in Alzheimer’s disease promotes synaptic tau accumulation and disrupts tau release

**DOI:** 10.1093/braincomms/fcaa011

**Published:** 2020-02-14

**Authors:** Elizabeth B Glennon, Dawn H W Lau, Rebecca M C Gabriele, Matthew F Taylor, Claire Troakes, Sarah Opie-Martin, Christina Elliott, Richard Killick, Diane P Hanger, Beatriz G Perez-Nievas, Wendy Noble

**Affiliations:** f1 Department of Basic and Clinical Neuroscience, Institute of Psychiatry, Psychology and Neuroscience, King’s College London, London SE5 9RX, UK; f2 MRC London Neurodegenerative Diseases Brain Bank, Institute of Psychiatry Psychology and Neuroscience, King’s College London, London SE5 8AB, UK; f3 Department of Old Age Psychiatry, Institute of Psychiatry, Psychology and Neuroscience, King’s College London, London SE5 9RX, UK

**Keywords:** Alzheimer’s disease, BIN1, genome‐wide association studies, tau, synapse

## Abstract

Polymorphisms associated with *BIN1* (bridging integrator 1) confer the second greatest risk for developing late-onset Alzheimer’s disease. The biological consequences of this genetic variation are not fully understood; however, BIN1 is a binding partner for tau. Tau is normally a highly soluble cytoplasmic protein, but in Alzheimer’s disease, tau is abnormally phosphorylated and accumulates at synapses to exert synaptotoxicity. The purpose of this study was to determine whether alterations in BIN1 and tau in Alzheimer’s disease promote the damaging redistribution of tau to synapses, as a mechanism by which *BIN1* polymorphisms may increase the risk of developing Alzheimer’s disease. We show that BIN1 is lost from the cytoplasmic fraction of Alzheimer’s disease cortex, and this is accompanied by the progressive mislocalization of phosphorylated tau to synapses. We confirmed proline 216 in tau as critical for tau interaction with the BIN1-SH3 domain and showed that the phosphorylation of tau disrupts this binding, suggesting that tau phosphorylation in Alzheimer’s disease disrupts tau–BIN1 associations. Moreover, we show that BIN1 knockdown in rat primary neurons to mimic BIN1 loss in Alzheimer’s disease brain causes the damaging accumulation of phosphorylated tau at synapses and alterations in dendritic spine morphology. We also observed reduced release of tau from neurons upon BIN1 silencing, suggesting that BIN1 loss disrupts the function of extracellular tau. Together, these data indicate that polymorphisms associated with BIN1 that reduce BIN1 protein levels in the brain likely act synergistically with increased tau phosphorylation to increase the risk of Alzheimer’s disease by disrupting cytoplasmic tau–BIN1 interactions, promoting the damaging mis-sorting of phosphorylated tau to synapses to alter synapse structure and reducing the release of physiological forms of tau to disrupt tau function.

## Teaser

Polymorphisms associated with bridging integrator 1 (BIN1) increase the risk of Alzheimer’s disease. We show that, in Alzheimer’s disease, cortex BIN1 is lost from the cytoplasmic fraction and phosphorylated tau accumulates at synapses. This can be mimicked by BIN1 knockdown in cultured neurons, which also caused disrupted dendritic morphology and tau release.

## Graphical Abstract

**Figure:**
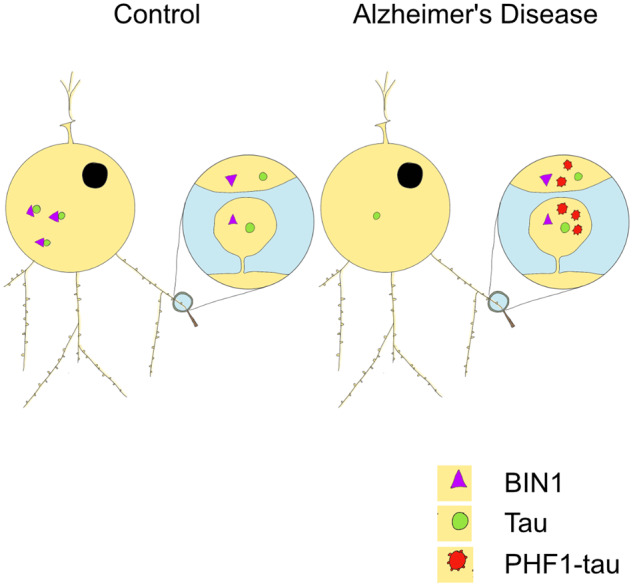

## Introduction

Tauopathies including Alzheimer’s disease are characterized by tau protein modifications that affect normal tau interactions and localization and lead to the development of neurofibrillary pathology (Guo *et al.*, 2017). The redistribution of highly phosphorylated and/or oligomeric tau species to pre- and post-synapses causes the disruption of synaptic vesicle mobility and neurotransmitter release (Zhou *et al.*, 2017; McInnes *et al.*, 2018) and excitotoxicity (Ittner *et al.*, 2010; Li and Gotz, 2017), respectively. As a result, the accumulation of phosphorylated tau at synapses is closely linked with synapse loss and dementia in Alzheimer’s disease (Perez-Nievas *et al.*, 2013; Hanseeuw *et al.*, 2019). Developing a better understanding of the causes of tau protein redistribution to synapses may elucidate potential new treatment strategies for Alzheimer’s disease and related tauopathies.

Recent genome-wide association studies have identified several gene variants that increase the risk of developing Alzheimer’s disease. Of those identified to date, polymorphisms associated with bridging integrator 1 (*BIN1*) confer the second largest genetic risk factor for developing sporadic Alzheimer’s disease, after the apolipoprotein E4 allele (*APOE4*) (Seshadri *et al.*, 2010; Hu *et al.*, 2011; Wijsman *et al.*, 2011; Lambert *et al.*, 2013; Naj *et al.*, 2014; Vardarajan *et al.*, 2015). Rare variants in coding regions of *BIN1* have been identified (Vardarajan *et al.*, 2015); however, the more common *BIN1* variants are upstream of the gene and do not affect protein structure. However, these may affect the tissue-specific splicing or expression of the cytoplasmic membrane-binding BIN1 protein, which is known to play important roles in endocytosis and subcellular trafficking (Prokic *et al.*, 2014). In support of this, the expression of the longer neuronal isoform of BIN1 is decreased and the shorter glial isoforms are increased in Alzheimer’s disease brain (Glennon *et al.*, 2013; Holler *et al.*, 2014; De Rossi *et al.*, 2016).

While most genetic variants that increase the risk of Alzheimer’s disease affect β-amyloid generation and/or clearance, BIN1 is relatively unusual in that its effects in Alzheimer’s disease appear to be mediated by tau (Chapuis *et al.*, 2013; Wang *et al.*, 2016). In Alzheimer’s disease brain, BIN1 may colocalize with neurofibrillary tangle-containing neurons (Holler *et al.*, 2014) and is associated with elevated tau phosphorylation (Wang *et al.*, 2016). Expression of BIN1 in a Drosophila model of Alzheimer’s disease was shown to modulate the toxicity of tau (Chapuis *et al.*, 2013) and the knockdown of BIN1 promotes tau propagation between neurons (Calafate *et al.*, 2016). Others have shown that BIN1 over-expression in mice causes microstructural changes in hippocampal circuits (Daudin *et al.*, 2018) that are among the first to show tau pathology in Alzheimer’s disease (Daudin *et al.*, 2018), suggesting that BIN1 may affect the development of Alzheimer’s disease by modulating tau effects at synapses, and possibly also synaptic activity-dependent tau release (Pooler *et al.*, 2013).

The effects of BIN1 on tau appear to be mediated by direct association between the two proteins. Interactions between BIN1 and tau have been demonstrated in cell models, Drosophila and mice (Chapuis *et al.*, 2013; Sottejeau *et al.*, 2015; Malki *et al.*, 2017; Sartori *et al.*, 2019*)*. BIN1 contains a src homology 3 (SH3) domain through which it interacts with prolines within PXXP motifs (Prokic *et al.*, 2014). Tau contains seven PXXP motifs in its proline-rich domain (Usardi *et al.*, 2011), and the SH3 domain of BIN1 interacts with the proline-rich region of tau in a phosphorylation-dependent manner (Chapuis *et al.*, 2013; Sottejeau *et al.*, 2015; Malki *et al.*, 2017; Sartori *et al.*, 2019). However, the mechanisms by which BIN1 affects tau to mediate pathological changes in tau proteins are not fully understood. The purpose of this study was to determine whether alterations in BIN1 and tau in Alzheimer’s disease promote the damaging redistribution of tau to synapses, as a mechanism by which BIN1 polymorphisms may increase the risk of developing Alzheimer’s disease.

## Materials and methods

### Human brain

Braak-staged post-mortem human temporal cortex was obtained from the Medical Research Council London Neurodegenerative Diseases Brain Bank at King’s College London following ethical approval (Research Ethics Committee reference: 08/MRE09/38 + 5). Neuropathological assessment was performed according to standard criteria. Samples were classified as control (no history of neurodegenerative or psychiatric disease and age-related pathology only), moderate Alzheimer’s disease (clinical diagnosis of Alzheimer’s disease and Braak stage III–IV pathology) and severe Alzheimer’s disease (clinical diagnosis of Alzheimer’s disease and Braak stage V–VI pathology). Characteristics of these samples are summarized in Tables 1 and 2.

**Table 1:** Summary of temporal cortex tissue used in this study

Sex	Age (years)	Post- mortem delay (h)	Braak stage	Diagnosis
F	74	64	II	Control
F	90	44	II	Control
F	73	27	I	Control
F	77	21	0	Control
F	80	22	II	Control
M	68	60	II	Control
M	80	55	II–III	Control
M	90	45	–	Control
M	78	24	III	Control
F	92	9	II	Control
M	82	47	I	Control
F	84	34	I–II	Control
F	90	50	II	Control
M	66	52	–	Control
M	82	18	I/II	Control
M	91	48	IV	Moderate Alzheimer’s disease
M	88	79	III–IV	Moderate Alzheimer’s disease
F	95	47	IV	Moderate Alzheimer’s disease
M	84	86	IV	Moderate Alzheimer’s disease
M	98	53	IV	Moderate Alzheimer’s disease
F	86	55.5	IV	Moderate Alzheimer’s disease
M	82	28	IV	Moderate Alzheimer’s disease
M	86	52.5	IV	Moderate Alzheimer’s disease
F	83	22	IV	Moderate Alzheimer’s disease
M	93	13.5	IV	Moderate Alzheimer’s disease
F	83	41.5	IV	Moderate Alzheimer’s disease
F	97	67.5	III–IV	Moderate Alzheimer’s disease
F	96	39	IV	Moderate Alzheimer’s disease
F	92	19.5	III	Moderate Alzheimer’s disease
F	92	29.5	IV	Moderate Alzheimer’s disease
F	73	30	VI	Severe Alzheimer’s disease
F	84	27	VI	Severe Alzheimer’s disease
F	79	31	VI	Severe Alzheimer’s disease
M	86	38	VI	Severe Alzheimer’s disease
F	85	79	VI	Severe Alzheimer’s disease
M	67	39.5	VI	Severe Alzheimer’s disease
F	69	73	VI	Severe Alzheimer’s disease
F	89	38.5	VI	Severe Alzheimer’s disease
F	93	49	VI	Severe Alzheimer’s disease
M	84	67	VI	Severe Alzheimer’s disease
F	81	20	VI	Severe Alzheimer’s disease
M	83	22	VI	Severe Alzheimer’s disease
F	81	17.5	VI	Severe Alzheimer’s disease
F	86	25	VI	Severe Alzheimer’s disease
M	66	41	VI	Severe Alzheimer’s disease

Table shows details of sex, age, post-mortem delay, Braak stage and Alzheimer’s disease diagnosis for cases from which frozen temporal cortex sections was obtained.

**Table 2:** Summary of temporal cortex cases and controls used in this study

Disease stage	Female (%)	Age (years), mean ± SEM	Post-mortem delay (h), mean ± SEM
Control	53.3	80.4 ± 2.07	38.1 ± 4.40
Moderate	57.1	89.6 ± 1.42	45.3 ± 5.50
Severe	64.2	80.9 ± 2.10	39.8 ± 5.00

Table shows the percentage of control, moderate Alzheimer’s disease and severe Alzheimer’s disease cases that were female, the mean age at death (± SEM) and the mean post-mortem delay (± SEM).

### Modification of BIN1 expression in primary neurons

All animal work was conducted in accordance with the UK Animals (Scientific Procedures) Act 1986 and the European Directive 2010/63/EU under UK Home Office Personal and Project Licenses and with agreement from the King’s College London (Denmark Hill) Animal Welfare and Ethical Review Board.

Pregnant female Sprague-Dawley rats were purchased from Charles River and were used within 1 day of delivery. Water and food were available (Picolab rodent diet 20; # 5053; Lab Diet, St Louis, MO, USA) *ad libitum*. Animals were housed at 19–22°C, humidity 55%, 12-h:12-h light:dark cycle with lights on at 07:30. Primary cortical neurons were dissected from embryonic day (E) 18 male and female rats and were cultured as previously described (Pooler *et al.*, 2012) on poly-d-lysine-coated plates or glass coverslips. Rat neurons were used since these provide a readily tractable cell model in which we can mimic findings from post-mortem human brain and they are a model in which ‘tau biology’ has been extensively studied. Lentivirus short hairpin RNA (shRNA) targeting BIN1 from the RNA interference consortium (TRCN0000088188) and a scrambled control sequence in the pLKO.1 vector were purchased from Dharmacon Horizon (CO, USA). PAX2 and pMG.2 lentiviral packaging vectors were kind gifts from Dr Maria Jimenez-Sanchez (King’s College London). Human embryonic kidney (HEK293) cells cultured in Dulbecco’s Modified Eagle’s Medium plus GlutaMAX (Thermo Fisher Scientific, MA, USA) supplemented with 10% (v/v) foetal bovine serum (Thermo Fisher Scientific) were transfected with PAX2, pMG.2 and shRNA lentivirus using Lipofectamine 2000 (Invitrogen, CA, USA). After 24 h, lentiviral particles were collected from culture medium, isolated and concentrated according to the manufacturer’s instructions. For lentiviral knockdown, neurons were cultured for 5 days *in vitro* (DIV) and then treated with either BIN1 targeting shRNA, scrambled or control shRNA lentiviral particles for 24 hours, after which time the virus was removed and neurons further cultured until 21–23 DIV prior to use. Alternatively, BIN1 was knocked down in primary neurons using Accell BIN1 small interfering RNA smart pool (E-095528) purchased from Dharmacon Horizon Discovery (UK). For these experiments, 19 DIV rat primary cortical neurons were transfected with 50 nM BIN1 or non-targeting control small interfering RNA (Dharmacon Horizon Discovery) using Lipofectamine 2000 for 96 h at 37°C, after which time neurons were imaged or harvested.

### Tau enzyme-linked immunosorbent assay and cell viability assays

Tau enzyme-linked immunosorbent assays were performed on Hank’s balanced salt solution with Ca^2+^ and Mg^2+^ medium that had been incubated for 4 h with 22–23 DIV primary neurons as we described previously (Croft *et al.*, 2017). The amount of lactate dehydrogenase in the media of cultured neurons was determined as a measure of neuron heath, using an LDH Cytotoxicity Kit from Thermo Fischer Scientific according to the manufacturer’s instructions.

### Immunofluorescence

Immunofluorescence was performed as described previously (Schurmann *et al.*, 2019), using 2% (v/v) foetal bovine serum (Life Technologies) in place of normal goat serum. Cells were incubated with primary antibodies against BIN1 (ab54764; Abcam), post-synaptic density 95 (PSD95, D74D3; Cell Signaling), synaptophysin (sc7568; Santa Cruz, TX, USA), glial fibrillary acidic protein (Agilent, ZO334) and microtubule-associated protein 2 (GTX82661; GeneTex) and the appropriate species of AlexaFluor-conjugated secondary antibodies (Life Technologies). Coverslips were mounted onto glass slides using the ProLong Diamond mounting media (Life Technologies). The labelled proteins were imaged under an Eclipse Ti2 inverted Nikon 3D structured illumination microscope, and images were reconstructed using Nikon Imaging Systems Elements software, or a Nikon Eclipse Ti2 inverted microscope with Vt-iSIM scan head and deconvolved using Nikon Imaging Systems Elements software.

### Analysis of synapses

Primary neurons were fractionated to generate cytosol- and synapse-enriched fractions using a protocol modified from Frandemiche *et al.* (2014). Total, cytosolic and synaptoneurosome fractions were isolated from post-mortem temporal cortex as we described previously (Perez-Nievas *et al.*, 2013). Equal protein amounts of total, synaptic and cytoplasmic fractions were immunoblotted.

For the analysis of dendritic spine structure, neurons at 22 DIV were transfected with an enhanced green fluorescent protein-N2 plasmid (Clontech, Kyoto, Japan) using Lipofectamine 2000, fixed 24 hours post-transfection, and the GFP-expressing cells were imaged using a Nikon Eclipse Ti2 inverted microscope with Vt-iSIM scan head. The 3 × 3 large image stacks were acquired covering the entire volume of the neuron, with 0.2 μm between each image in the Z plane. Neurolucida™ software (MBF Bioscience, VT, USA) was used to trace neurons and detect, classify and quantify dendritic spines and perform the Scholl analysis. Neuronal complexity was determined by (sum of the terminal orders + number of terminals) × (total dendritic length/number of primary dendrites), where terminals are the number of branch endings and terminal orders are the number of branches between the terminal and the cell body.

### Glutathione-*S*-transferase binding assays

BIN1-SH3 cDNA generously provided by Isabelle Landrieu (University of Lille Nord de France) was cloned into pGEX5X1 vectors using sequence- and ligation-independent cloning (Hill and Eaton-Rye, 2014). The BIN1-SH3 domain was amplified from the original vector using primers 5ʹ-TCG AGC GGC CGC ATC GTG ACA TGG GTC GTC TGG ATC TG-3ʹ and 5ʹ-AAA CGC GCG AGG CAG ATC GTC AGT TAC GGC ACA CGC TCA GTA AAA TTC-3ʹ, and pGEX5X1 was linearized using primers 5ʹ-CTG ACG ATC TGC CTC GCG-3ʹ and 5ʹ-GTC ACG ATG CGG CCG CTC-3ʹ. Sequence- and ligation-independent cloning products were used to transform BL21 *E. coli* (New England Biolabs, MA, USA) by heat shock. DNA was purified using the QIAgen Spin Miniprep Kit (QIAgen, Hilden, Germany), and the cloning was confirmed by sequencing (Source Bioscience, Nottingham, UK), using stock primers to the glutathione-*S*-transferase (GST) plasmid. BL21 *E. coli* containing either BIN1-SH3-pGEX5X1 or empty vector pGEX5X1 was used to produce GST fusion proteins. Wild-type human 2N4R tau and PXXP mutant tau plasmids have been described previously (Lau *et al.*, 2016). These were expressed in HEK293 cells for 24 h after which time cells were lysed and the lysates used for GST pull-downs, which were performed as we described previously (Lau *et al.*, 2016).

### Sodium Dodecyl Sulfate–Polyacrylamide Gel Electroresis and western blotting

Protein concentrations of samples were determined using a BCA protein assay kit (Thermo Fisher Scientific) and Ponceau Red staining of membranes. Equal protein amounts were electophoresed on 10% tris-glycine SDS-polyacrylamide gels, Nu-Page 4–12% or 10% bis–tris gels (Invitrogen), transferred to 0.45-μm nitrocellulose membrane (Millipore, MA, USA) and immunoblotted using standard methods. Primary antibodies were BIN1 (99D; Millipore), GST (GE Healthcare, IL, USA), total tau (total human tau; Agilent), Tau-1 (Millipore), PHF1 (Peter Davies, Donald and Barbara Zucker School of Medicine at Hofstra, Northwell), *N*-methyl-d-aspartate subunit 2B (06-600; Millipore), β-actin (ac15; Abcam), synaptophysin (sc17750; Santa Cruz), PSD95 (MAB 1596; Millipore) and Fyn (HPA023887; Sigma). The bound horseradish peroxidase-conjugated secondary antibodies (GE Healthcare) were detected using enhanced chemiluminesence solutions (Thermo Fisher Scientific) and visualized using a ChemiDoc imager (Bio-Rad, CA, USA). Densitometric analysis was performed using FIJI.

### Data analysis and statistics

Statistical tests were performed using GraphPad Prism 7.0 (CA, USA) or RStudio. Normality tests were performed on all data, and the appropriate statistical tests were then used to determine differences between experimental groups. Tests used and sample size (n) numbers are provided for each experiment in the figure legends.

### Data availability

The data supporting this study are available in the manuscript and Supplementary Material, and raw data will be made available on reasonable request following publication. Summary statistics including exact *P*-values, *F*-values and degrees of freedom are included in the Supplementary Material.

## Results

### BIN1 loss in cytoplasmic fractions correlates with increased synaptic tau in Alzheimer’s disease brain

**Figure 1:**
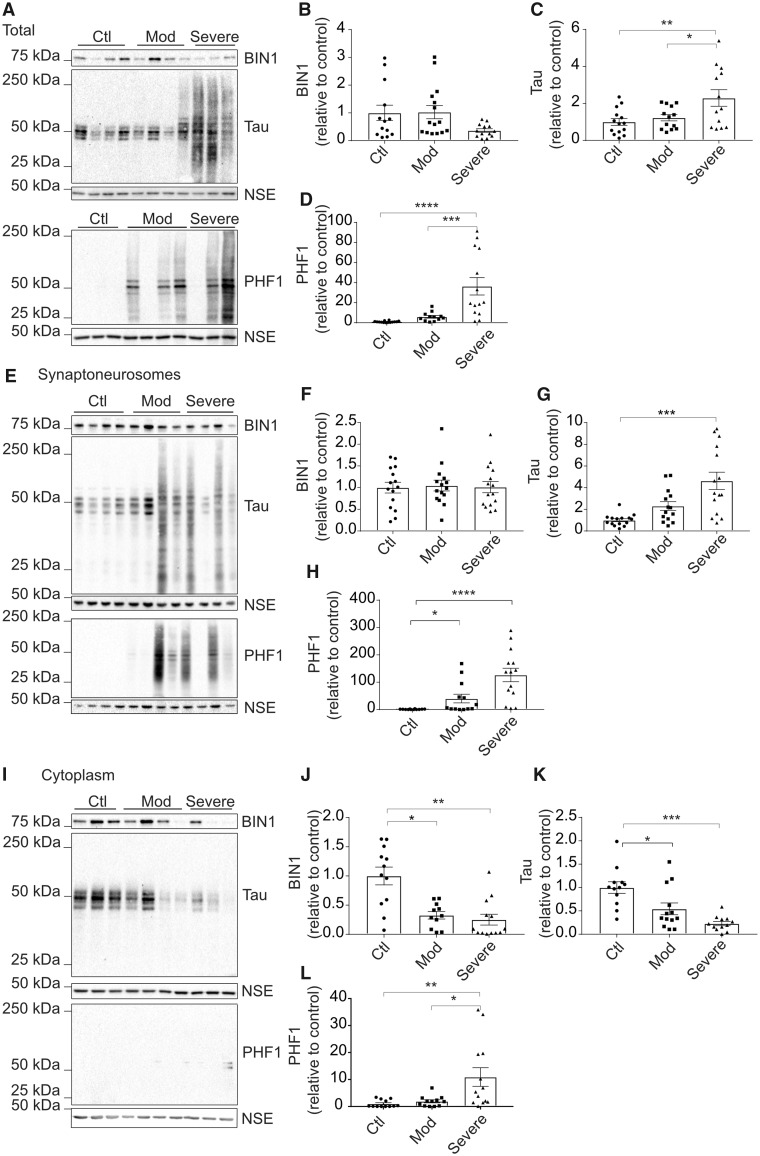
**BIN1 and tau are lost from the cytoplasm, and this is associated with the accumulation of phosphorylated tau at synapses in Alzheimer’s disease temporal cortex.** (**A**) Total homogenates from temporal cortex of control (Braak stage 0–III), moderate (Braak stage III–IV) and severe (Braak stage V–VI) Alzheimer’s disease brain were western blotted using antibodies against BIN1, total tau, phosphorylated tau (PHF1, pSer396/404) and neuron-specific enolase (NSE) as a loading control. Bar charts show the quantification of (**B**) BIN1, (**C**) tau and (**D**) tau phosphorylated at Ser396/404 (PHF1). Data shown are mean ± SEM expressed as fold average control. *n* = 13 per group (BIN1), *n* = 14 per group (tau) or *n* = 12 per group (PHF1). Following D’Agostino and Pearson normality testing, data were analysed using a one-way ANOVA with Holm–Sidak’s multiple comparisons test. (**E**) Synaptoneurosomes isolated from the same temporal cortex samples were immunoblotted with the same antibodies. Bar charts show the quantification of (**F**) BIN1, (**G**) tau and (**H**) PHF1 in synaptoneurosome fractions following normalization. Data are mean ± SEM expressed as fold average control. *n* = 15 per group (BIN1 and tau) or *n* = 12 per group (PHF1). Following D’Agostino and Pearson normality testing, data were analysed using non-parametric Kruskal–Wallis test with Dunn’s multiple comparison test. (**I**) The cytoplasmic fraction was blotted as above with antibodies against BIN1, tau, PHF1 and NSE. Bar charts show the quantification of (**J**) BIN1, (**K**) tau and (**L**) tau phosphorylated at Ser396/404 (PHF1) in the cytoplasmic fraction following normalization to NSE in the same sample. Data are mean ± SEM expressed as fold mean control. *n* = 11 per group (BIN1) or *n* = 12 per group (tau and PHF1). Following D’Agostino and Pearson normality testing, BIN1 data were analysed using non-parametric Kruskal–Wallis test with Dunn’s multiple comparison test and tau data using a one-way ANOVA with Holm–Sidak’s multiple comparisons test. *P < 0.05, **P < 0.01, ***P < 0.001 and ****P < 0.0001. Full uncut western blots are found in the Supplementary Material.

In Alzheimer’s disease, highly phosphorylated tau is mislocalized to synaptic compartments (Perez-Nievas *et al.*, 2013) where tau disrupts synapse function and mediates synaptotoxicity (Ittner *et al.*, 2010; Li and Gotz, 2017; Zhou *et al.*, 2017; McInnes *et al.*, 2018). The longest neuronal isoform of BIN1 protein is reduced in end-stage Alzheimer’s disease brain (Glennon *et al.*, 2013; De Rossi *et al.*, 2017). To determine whether BIN1 is lost at the earlier stages of Alzheimer’s disease, and whether its loss is associated with changes in the distribution of tau, we isolated synaptoneurosomes (Perez-Nievas *et al.*, 2013) from control (Braak stage 0–III), moderate (Braak stage III–IV) and severe (Braak stage V–VI) post-mortem Alzheimer’s disease temporal cortex and examined total, cytosolic and synaptic fractions on western blots (Fig. 1). The integrity of synaptic proteins in these samples was first confirmed, as described previously (Bayes *et al.*, 2014), by western blotting a subset of samples with an antibody against *N*-methyl-d-aspartate subunit 2B (Supplementary Fig. 1B and C). Relatively little degeneration of synaptic proteins indicating that acceptable levels of synaptic integrity is maintained in these tissues (Bayes *et al.*, 2014), and showed no differences in synaptic integrity between groups.

**Figure 2:**
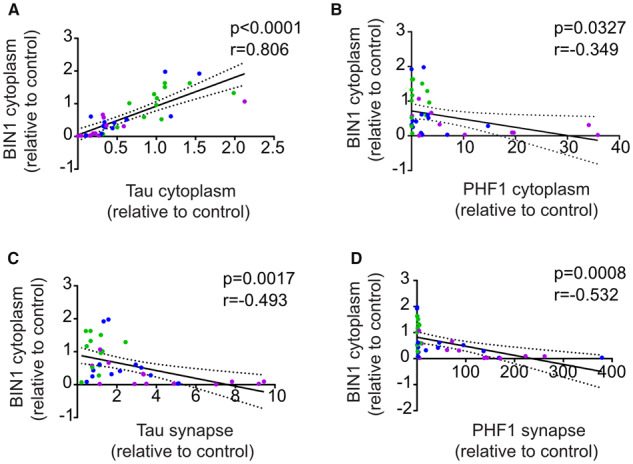
**Loss of BIN1 correlates with the loss of cytoplasmic tau and increased synaptic tau in Alzheimer’s disease temporal cortex.** Correlation analysis of (**A**) BIN1 and tau amounts and (**B**) BIN1 and phosphorylated (pSer396/404, PHF1) tau amounts in the cytoplasmic fractions showing a strong positive correlation between BIN1 and total tau, a negative correlation between BIN1 and phosphorylated tau (*n* = 38) and strong negative correlations between (**C**) cytoplasmic BIN1 and synaptic tau (*n* = 38) and (**D**) cytoplasmic BIN1 and synaptic tau phosphorylated at Ser396/404 (PHF1) (*n* = 36). Colours represent control (green), moderate (blue) and severe (purple) Alzheimer’s disease samples.

In total brain homogenates, we confirmed a trend towards reduction in BIN1 in severe relative to moderate stage Alzheimer’s disease and control tissues (Fig. 1A and B) and significant increases in total and phosphorylated tau amounts with increasing disease severity (Fig. 1A, C and D), as previously reported (Kurbatskaya *et al.*, 2016). Protein amounts were normalized to neuron-specific enolase in the same sample prior to quantification to control for any effects of neuronal loss and/or gliosis (Kurbatskaya *et al.*, 2016). Tau phosphorylation was detected using an antibody against tau phosphorylated at S396/404 (PHF1). The cytoplasmic and synaptoneurosome fractions isolated from the same brain samples were characterized to confirm their relative purity (Supplementary Fig. 1A). Blotting of these samples showed a significant accumulation of tau and phosphorylated tau in the synaptic compartment in severe Alzheimer’s disease brain, relative to moderate staged samples and controls (Fig. 1E, G and H). Tau phosphorylated at S396/S404 was found to accumulate at synapses in both moderate and severe stage Alzheimer’s disease relative to controls (Fig. 1E and H). The accumulation of synaptic tau paralleled the loss of cytoplasmic tau (Fig. 1I, K and L), suggesting that these results reflect tau mis-sorting from the cytoplasm to synapses. There were no differences in BIN1 levels in synaptoneurosomes between groups (Fig. 1E and F). However, we found marked and significant losses of cytoplasmic BIN1 in both moderate and severe Alzheimer’s disease tissues (Fig. 1I and J) that correlated positively with reductions in cytoplasmic tau (Fig. 2A) and inversely with increased synaptic tau (Fig. 2C and D). We also found negative correlations between levels of phosphorylated (pSer396/404, PHF1) tau and BIN1 in the cytoplasmic fraction (Fig. 2B). There were also no significant correlations between total tau or phosphorylated tau and BIN1 amounts in either the total or synaptoneurosome fractions (Supplementary Fig. 1G–J). Taken together, these data suggest that loss of cytoplasmic BIN1 may facilitate the mis-sorting of phosphorylated tau to the synapse.

### BIN1 knockdown causes synaptic accumulation of phosphorylated tau in neurons

**Figure 3:**
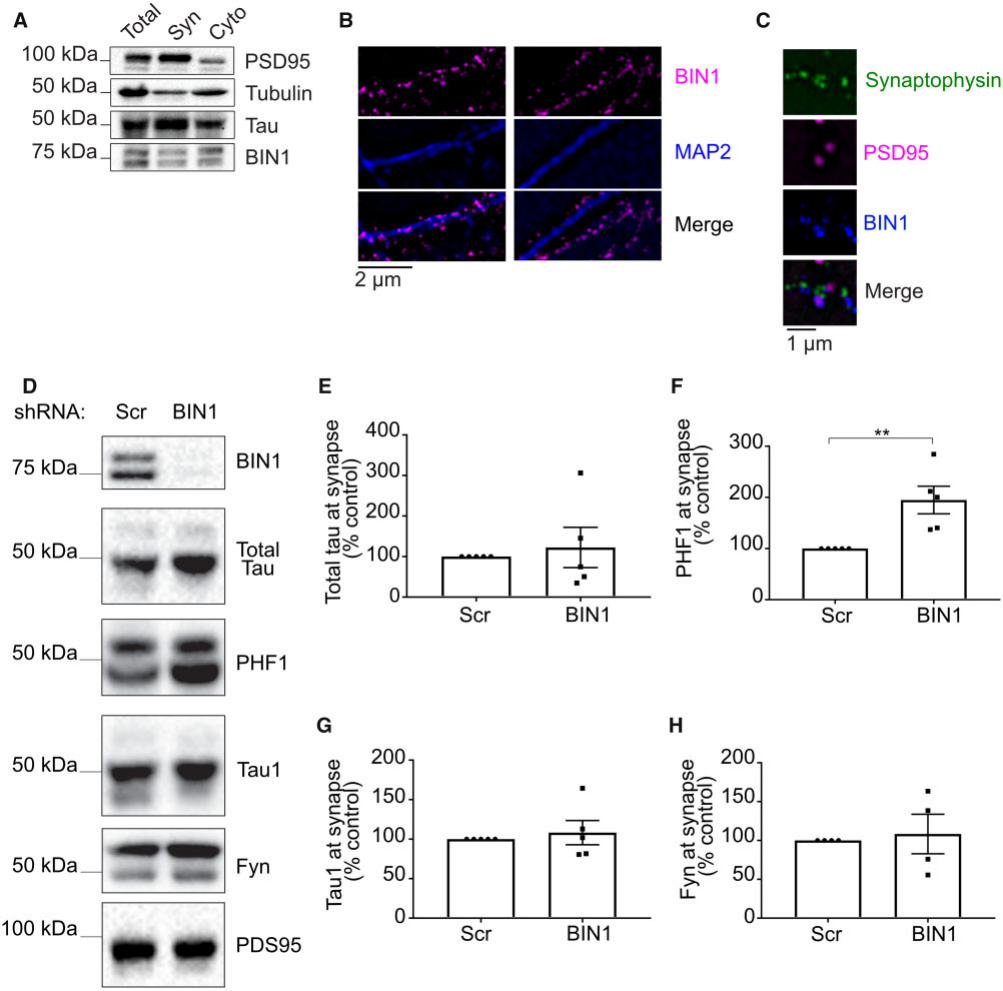
**BIN1 knockdown in neurons increases the abundance of phosphorylated tau at synapses.** (**A**) Proteins from 22 DIV primary cortical neurons were biochemically fractionated using a synaptosome fractionation protocol into total, synaptic protein- (syn) and cytoplasmic (cyto) protein-enriched fractions, which were western blotted with antibodies against PSD95, tubulin, tau and BIN1. Blots show the presence of BIN1 and tau in all fractions and an enrichment of PSD95 in the synaptic relative to the cytosolic fraction. (**B**) N-SIM super-resolution images of primary cortical neurons immunolabelled with antibodies against BIN1 (ab54764, pink) and the dendritic marker MAP2 (blue) showing that BIN1 is present within dendrites and axons in cultured neurons. (**C**) N-SIM super-resolution images show close associations and some colocalization of BIN1 (ab54764, blue) with the pre-synaptic marker synaptophysin (green) and the post-synaptic marker PSD95 (pink). (**D**) Lysates from primary cortical neurons transduced with scrambled control shRNA (Scr) lentivirus or BIN1 shRNA (BIN1) lentivirus at 5 DIV and biochemically fractionated at 22 DIV as above were immunoblotted with antibodies against BIN1, total tau, tau phosphorylated at Ser396/404 (PHF1), tau dephosphorylated at Ser199/202/Thr205 (Tau-1), Fyn kinase and PSD95. Bar charts show the quantification of synaptic (**E**) total tau, (**F**) tau phosphorylated at Ser396/404 (PHF1), (**G**) dephosphorylated tau (Tau-1) and (**H**) Fyn protein amounts. Data were normalized to the synaptic marker PSD95 in the same sample and are expressed as percentage mean control (scrambled shRNA). Data are mean ± SEM and were analysed using Mann–Whitney test. *n* = 4-5, **P < 0.01. Full uncut western blots are found in the Supplementary Material. MAP2 = microtubule-associated protein 2; N-SIM = Nikon 3D structured illumination microscope.

To model BIN1 loss and tau mislocalization to synapses *in vitro*, we silenced BIN1 in rat primary cortical neurons using lentivirus. We confirmed the efficiency of BIN1 knockdown by western blotting and proximity ligation assay (Supplementary Fig. 2). In control neurons, BIN1 and tau are found in fractions enriched in cytoplasmic and synaptic proteins (Fig. 3A). Nikon 3D structured illumination microscope imaging of neuronal processes showed that BIN1 decorates microtubule-associated protein 2-positive and microtubule-associated protein 2-negative fibres (Fig. 3B) and localizes in close proximity to pre-synaptic (synaptophysin) and post-synaptic (PSD95) markers, with a portion of BIN1 co-localizing with PSD95 (Fig. 3C). Cultures were also immunolabelled with an antibody against glial fibrillary acidic protein, which suggests that a small proportion of BIN1 is astrocytic (Supplementary Fig. 2I and J), in agreement with recent reports (Taga *et al.*, 2019).

We found that knockdown of BIN1 did not alter the total amount of tau or its phosphorylation in total cell lysates (Supplementary Fig. 2A–D). However, following BIN1 knockdown, there was a significant increase in the amounts of phosphorylated, but not total or dephosphorylated, tau in synapse-enriched fractions relative to controls (Fig. 3D–G). These data show that reducing BIN1 in neurons causes the accumulation of phosphorylated tau at synapses and suggest that the increased synaptic phospho-tau that we observe in post-mortem Alzheimer’s disease brain may result from tau mislocalization from the cytoplasm upon the loss of cytoplasmic BIN1.

When bound to tau, the non-receptor tyrosine kinase, Fyn, is trafficked to the post-synapses where it is believed to mediate β-amyloid toxicity in Alzheimer’s disease (Ittner *et al.*, 2010). We previously showed that the Fyn-SH3 domain also binds preferentially to proline 216 in tau (Usardi *et al.*, 2011; Pooler *et al.*, 2012). We therefore examined the localization of Fyn in synaptic fractions following BIN1 knockdown. Our data suggest that there is no competition between BIN1 and Fyn for binding to tau since we found no alterations in synaptic Fyn amounts in neurons treated with *BIN1* shRNA (Fig. 3H).

**Figure 4:**
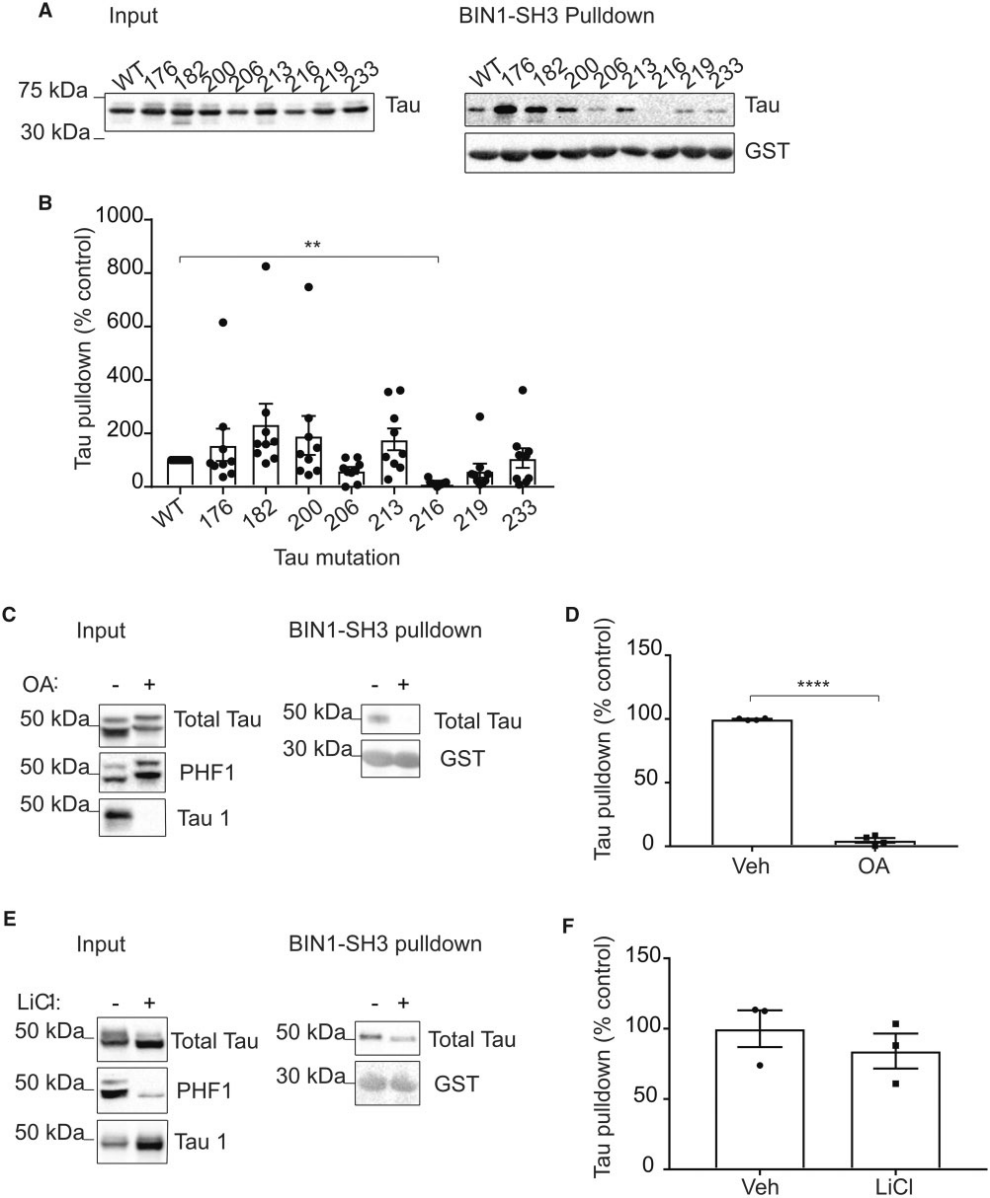
**BIN1 and tau interact via BIN1-SH3 and P216 in tau in a phosphorylation-dependent manner.** (**A**) HEK293 cells were transfected with WT 2N4R tau or PXXP mutant tau constructs in which a single proline residue at sites 176, 182, 200, 206, 213, 216, 219 or 223 was mutated to alanine to disrupt the PXXP sequence. Proteins in lysates from HEK293 cells (input) were pulled down with BIN1-SH3-GST beads and western blotted with antibodies against total tau or GST. (**B**) The amount of WT or PXXP mutant tau pulled down by BIN1-SH3-GST was quantified, and the bar chart shows this data relative to WT 2N4R tau (control). When P216 was mutated to alanine, tau binding to BIN1-SH3 was significantly reduced (**P < 0.001). Following D’Agostino and Pearson normality testing, data were analysed using non-parametric Kruskal–Wallis test and Dunn’s multiple comparisons test. Data shown are mean ± SEM, *n* = 9. (**C**) Primary cortical neurons were treated with either vehicle (−) or 50 nM OA (+) for 4 h. Proteins were pulled down from primary neuron lysates with BIN1-SH3-GST. Western blots of neuronal lysates (input) with antibodies against total tau, tau phosphorylated at Ser394/404 (PHF1) and tau dephosphorylated at Ser199/202/Thr 205 (Tau-1) show increased tau phosphorylation following OA treatment. (**D**) Quantification shows that the amount of tau pulled down by BIN1-SH3-GST is reduced following OA treatment of primary neurons. Data are shown as percentage relative to the mean of controls (vehicle). Following Shapiro–Wilk normality testing, the data were analysed using an unpaired *T*-test. Data shown are mean ± SEM, *n* = 4. ****P < 0.0001. (**E**) Lysates from primary cortical neurons show reduced tau phosphorylation following treatment with 25 mM LiCl (+) for 4 h relative to vehicle-treated neurons (−). BIN1-SH3-GST pull-downs show that there was no difference in the amount of tau pulled down by BIN1-SH3-GST following LiCl treatment relative to vehicle-treated conditions. (**F**) Quantification shows no difference in the amount of tau from vehicle- or LiCl-treated neurons pulled down by BIN1-SH3-GST. Following Shapiro–Wilk normality testing, data were analysed using a non-parametric Mann–Whitney test. Data shown are mean ± SEM, *n* = 3. Full uncut western blots are found in the Supplementary Material. LiCl = lithium chloride; OA = okadaic acid; WT = wild type.

The interaction of BIN1 with tau is reported to be regulated by tau phosphorylation and via direct association of the BIN1-SH3 domain and the proline-rich region of tau (Sottejeau *et al.*, 2015; Lasorsa *et al.*, 2018). To confirm this, we generated GST-BIN1-SH3 constructs (Supplementary Fig. 3A) and we used these and GST-only constructs in binding assays using lysates from HEK293 cells transfected with wild-type human 2N4R tau, mutant tau constructs or empty vector. The mutant tau constructs are human 2N4R tau in which a single proline in each PXXP motif is mutated to alanine (Supplementary Fig. 3B), as we reported previously (Lau *et al.*, 2016). Immunoblotting of BIN1-SH3-GST pull-downs with an antibody against tau confirmed that the BIN1-SH3 domain binds human 2N4R tau (Supplementary Fig. 3A and Fig. 4A and B). The analysis of BIN1-SH3 binding to mutant relative to wild-type human tau showed that P216 is important for the tau–BIN1 interaction. The amount of P216A tau bound to BIN1-SH3 was significantly decreased relative to wild-type tau. There were no significant differences relative to wild type in tau binding to BIN1-SH3 when any other proline residue was mutated to alanine (Fig. 4A and B).

We next confirmed that increasing tau phosphorylation, to mimic tau modifications in Alzheimer’s disease, affects the interaction of tau with BIN1 in cultured primary rat neurons. Cells were treated for 4 h with 50 nM okadaic acid, a protein phosphatase inhibitor that prevents the removal of phosphate from residues throughout the tau molecule to allow efficient tau phosphorylation (Van Dolah and Ramsdell, 1992; Pooler *et al.*, 2012) (Fig. 4C). GST pull-downs with lysates from these cells confirmed that phosphorylated tau shows only trace amounts of binding to BIN1-SH3-GST when compared with lysates from vehicle-treated cells (Fig. 4D). When tau was dephosphorylated upon application of 25 mM of the glycogen synthase kinase-3 inhibitor lithium chloride (Stambolic *et al.*, 1996; Pooler *et al.*, 2012) (Fig. 4E and F), or 20 μM of the casein kinase-1 inhibitor IC261 (Pooler *et al.*, 2012) (Supplementary Fig. 4), the amount of tau pulled down by BIN1-SH3 was similar to controls. Glycogen synthase kinase-3 and casein kinase-1 inhibitors were used to modulate tau phosphorylation since these kinases phosphorylate tau throughout the protein (Guo *et al.*, 2017), and phosphorylation of tau sites distal and proximal to P216 mediates tau interactions with BIN1-SH3 (Sottejeau *et al.*, 2015; Lasorsa *et al.*, 2018). These data confirm and extend previous findings to show that BIN1-SH3 interacts with P216 in tau, predominantly when tau is dephosphorylated. Taken together, our data suggest that tau phosphorylation in Alzheimer’s disease may disrupt cytoplasmic BIN1–tau interactions and allow unbound tau to mislocalize to synapses.

### Loss of BIN1 alters spine morphology and reduces tau release from neurons

Synaptic tau is closely linked with the release and propagation of modified tau proteins in Alzheimer’s disease and related tauopathies (Guo *et al.*, 2017; Yamada, 2017). However, the release of soluble tau species in physiological conditions allows important signalling roles of extracellular tau (Gomez-Ramos *et al.*, 2008; Pooler *et al.*, 2013) and this tau function may be lost in Alzheimer’s disease (Croft *et al.*, 2017).

**Figure 5:**
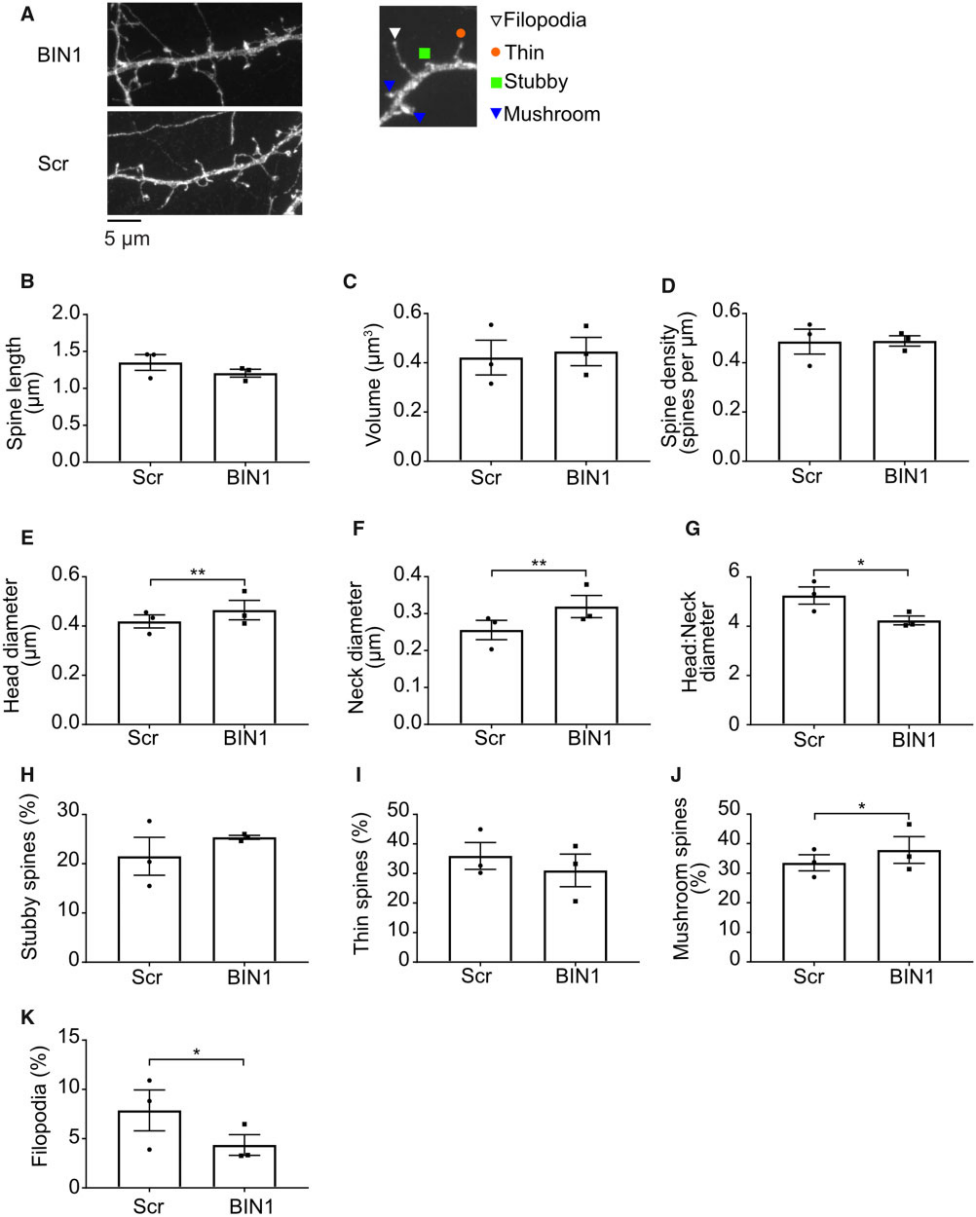
**BIN1 knockdown alters dendritic spine morphology.** (**A**) Primary cortical neurons transduced with BIN1 shRNA (BIN1) lentivirus or scrambled control shRNA (Scr) lentivirus at 5 DIV were transfected with a plasmid expressing eGFP and fixed at 23 DIV. Maximum intensity projections were generated from Z-stacks acquired using I-SIM super-resolution imaging. Five to ten different neurons per condition were analysed in each of three separate experiments, one dendrite from each cell was selected randomly for spine quantification and all branches of that dendrite were analysed. Dendritic spines were classified as either filopodia, stubby, thin or mushroom spines (right). Bar charts show the quantification of spine (**B**) length, (**C**) volume, (**D**) density, (**E**) head diameter, (**F**) neck diameter, (**G**) ratio of spine head to neck diameter and percentage of (**H**) stubby, (**I**) thin, (**J**) mushroom spines and (**K**) filopodia. Data are mean ± SEM and were analysed using a randomized block 2-way ANOVA. *n* = 3. *P < 0.05 and **P < 0.01. eGFP = enhanced green fluorescent protein.

Tau release is predominantly mediated by synaptic activity, and modulating BIN1 expression affects dendritic spine morphology and α-amino-3-hydroxy-5-methyl-4-isoxazolepropionic acid (AMPA) receptor-mediated synaptic transmission via changes in AMPA receptor surface expression and trafficking (Daudin *et al.*, 2018; Schurmann *et al.*, 2019). Since we and others have previously shown that neuronal depolarization and stimulation of AMPA receptors mediate endogenous tau release (Pooler *et al.*, 2013; Croft *et al.*, 2017), we examined the effects of BIN1 knockdown by shRNA on synapse morphology and tau release. Examination of neurons by iSIM showed that BIN1 knockdown affects synaptic morphology in 23 DIV primary neurons exogenously expressing enhanced green fluorescent protein. BIN1 knockdown did not cause any alterations in dendritic spine length, volume or density (Fig. 5A–D) but resulted in significant increases in the diameter of spine heads and necks (Fig. 5E and F) and a reduced head:neck diameter ratio (Fig. 5G). When spine morphologies were examined, BIN1 knockdown had no effect on the proportion of immature stubby or thin spines (Fig. 5H and I) but significantly reduced the proportion of filopodia (Fig. 5K) and increased the proportion of mature mushroom spines (Fig. 5J), which are relatively stable and have a high density of AMPA receptors (Hanley, 2008; Lee *et al.*, 2012; Woolfrey and Srivastava, 2016). The structure and density of dendritic spines varies according to the branch order of the neurite, and Scholl analysis (Supplementary Fig. 5) showed that BIN1 knockdown increases the branching of dendrites and increases neuronal complexity, which may contribute to the difference in spine structure that we observe since spines on all branches of neurites were quantified.

**Figure 6:**
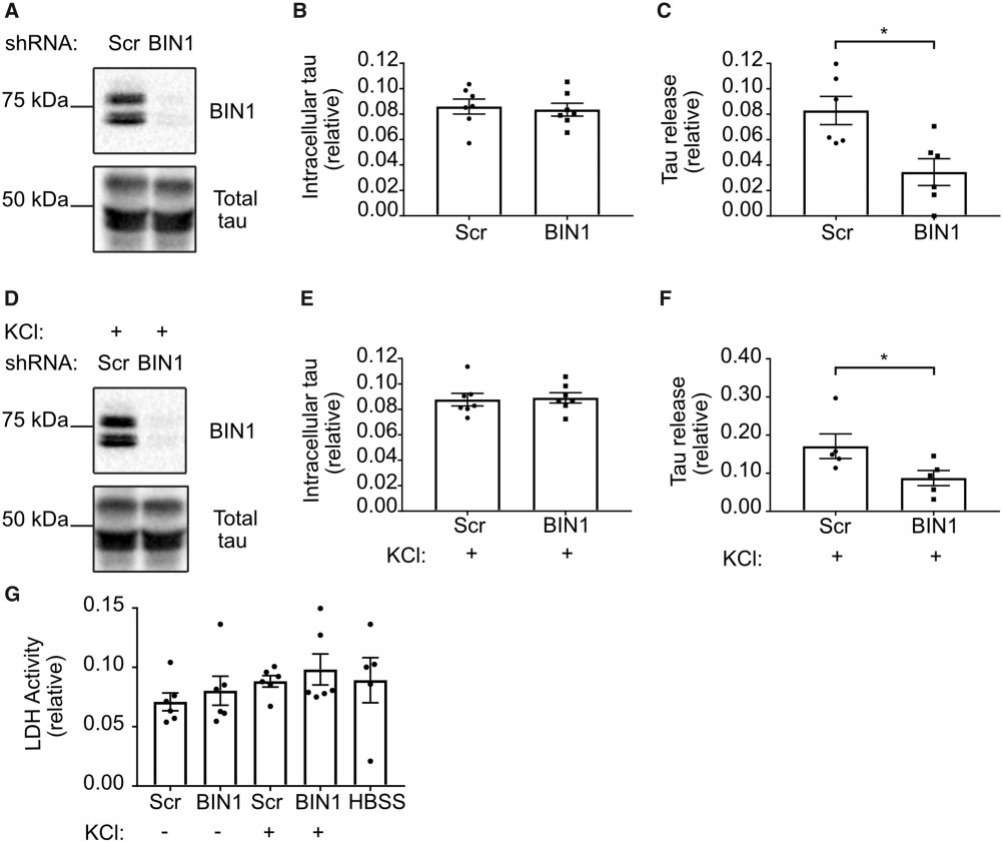
**BIN1 knockdown reduces basal and stimulated tau release.** (**A**) Cell lysates from 21 DIV primary cortical neurons transduced with scrambled control shRNA (Scr) lentivirus or BIN1 shRNA (BIN1) lentivirus at 5 DIV were western blotted with antibodies against BIN1 and total tau. (**B**) Quantification shows no effect of BIN1 knockdown on intracellular tau amount. Shapiro–Wilk normality test demonstrated that the data were normally distributed, and so data were analysed using an unpaired *T*-test. (**C**) Tau content in conditioned media from the same neurons was determined by ELISA. Extracellular tau amounts were quantified relative to intracellular tau from the same well, and the data show reduced tau release upon BIN1 knockdown. Shapiro–Wilk normality test demonstrated that the data were normally distributed, so data were analysed using an unpaired *T*-test. (**D**) Cells transduced as above were depolarized with 50 nM KCl (+) for 30 min, and the lysates were western blotted with antibodies against BIN1 and total tau. (**E**) KCl treatment had no effect on intracellular tau amounts. Shapiro–Wilk normality test demonstrated that the data were normally distributed, so data were analysed using an unpaired *T*-test. (**F**) Tau in conditioned media from KCl-stimulated cells was measured as described for basal conditions. Tau release from neurons in which BIN1 was knocked down was reduced upon neuron depolarization with KCl. Shapiro–Wilk normality test demonstrated that the data were not normally distributed, so data were analysed using a Mann–Whitney test. (**G**) Lactate dehydrogenase amounts were measured in medium from unstimulated (−) or KCl-stimulated (+) primary cortical neurons transduced with scrambled control shRNA (Scr) lentivirus or BIN1 shRNA (BIN1) lentivirus as above and show no effect of treatment on cell viability. Shapiro–Wilk normality test demonstrated that the data were not normally distributed, so data were analysed using a Kruskal–Wallis test with Dunn’s multiple comparison test. All graphs show mean ± SEM, *n* = 7 (intracellular tau), *n* = 6 (tau release/intracellular tau and lactate dehydrogenase assay). *P < 0.05. Full uncut western blots are found in the Supplementary Material. ELISA = enzyme-linked immunosorbent assay; KCl = potassium chloride.

To determine whether BIN1 knockdown affects tau release, tau content in culture medium from 21 DIV neurons treated with BIN1 and control shRNA was measured by enzyme-linked immunosorbent assay and normalized to the total amount of tau in neurons from the same culture well, as we reported previously (Croft *et al.*, 2017). BIN1 knockdown caused a significant reduction in basal tau release without affecting the amount of intracellular tau (Fig. 6A–C). BIN1 knockdown followed by the depolarization of neurons with potassium chloride to stimulate tau release (Pooler *et al.*, 2013; Croft *et al.*, 2017) also significantly reduced tau release relative to controls (Fig. 6D–F). The observed changes in tau release were not due to cell toxicity since there were no alterations in lactate dehydrogenase content in medium between conditions (Fig. 6G). Thus, BIN1 knockdown results in alterations in synapse morphology and reduces basal and stimulated tau release. Our findings, therefore, suggest that since BIN1 knockdown affects tau release, loss of BIN1 in Alzheimer’s disease will disrupt the functions of extracellular tau in addition to allowing phosphorylated tau to mislocalize to synapses and exert toxicity. Our data provide novel insights into the mechanisms by which *BIN1* polymorphisms may increase the risk of Alzheimer’s disease.

## Discussion

BIN1 is closely linked with tau abnormalities that underlie the progression of sporadic Alzheimer’s disease (Chapuis *et al.*, 2013; Calafate *et al.*, 2016; Dourlen *et al.*, 2019; Sartori *et al.*, 2019). Our results suggest that BIN1 and tau interact predominantly when tau is dephosphorylated in the cytoplasm and that altered tau phosphorylation, together with BIN1 loss in Alzheimer’s disease, allows tau to be mislocalized to synapses where it is detrimental to synapse health. In support of this, we found that P216 of tau interacts with the BIN1-SH3 and that interactions between these two proteins are disrupted when tau phosphorylation is increased in primary neurons. Our results also indicated that there may be some loss of tau interactions with BIN1-SH3 when tau proline 219 was mutated to alanine. Notably, even when an apparent (but not statistically validated) outlier was removed from this analysis, binding of BIN1-SH3 with P219A mutant tau was not significantly reduced. However, others have reported that proline residues 216 and 219 of tau are in direct contact with BIN1 (Lasorsa *et al.*, 2018). Our findings also showed that treatment with OA to increase tau phosphorylation effectively abolished tau interactions with BIN1. This is in keeping with observations of tau interactions with other SH3 domain-containing proteins such as Fyn (Reynolds *et al.*, 2008; Usardi *et al.*, 2011; Pooler *et al.*, 2012). Nevertheless, it is somewhat surprising that the binding of tau with BIN1 is not increased from baseline when tau is dephosphorylated since a degree of tau phosphorylation is expected in basal cell culture conditions. Future experiments using tau phosphomimics in which tau residues are mutated to mimic permanent phosphorylation or dephosphorylation, or conducting dose response experiments with tau phosphorylation modulators, will be useful to fully examine the effects of tau phosphorylation on its interactions with BIN1.

Loss of BIN1 from Alzheimer’s disease cytoplasm at moderate and severe stages of disease was accompanied by the accumulation of phosphorylated tau in synaptic compartments. The average age of patients classified as ‘moderate’ Alzheimer’s disease on the basis of Braak stage was found to be significantly higher than those with severe Alzheimer’s disease, and this may indicate that our ‘severe’ Alzheimer’s disease group consisted of cases with increased resilience to the accumulation of Alzheimer’s disease pathology. Our analysis of the relationship between phosphorylated tau and BIN1 subcellular distributions was conducted independently of Braak stage and, therefore, we do not consider this to confound our findings. However, it could be of interest to examine the relationship between tau and BIN1 further in well-matched resilient and susceptible populations in future studies.

To directly ascertain the effects of BIN1 loss on tau localization, we knocked down BIN1 in rat primary neurons and showed that this caused an accumulation of phosphorylated tau at synapses. This finding is in agreement with previous reports that over-expression of BIN1 in mice expressing human tau leads to a reduced number of somatodendritic tau inclusions (Sartori *et al.*, 2019). This is an important concept since alterations in the trafficking and normal positioning of tau are considered to be early pathogenic changes in Alzheimer’s disease (Zempel and Mandelkow, 2014; Zempel and Mandelkow, 2015; Guo *et al.*, 2017). We also investigated the effects of BIN1 knockdown on synapse structure and tau release, since in addition to promoting the spread of tau pathology (Yamada, 2017), synapse function is important for the release of physiological forms of tau and, therefore, the signalling functions of extracellular tau (Gomez-Ramos *et al.*, 2008). We show here that BIN1 knockdown affects the morphology of dendritic spines, a feature closely linked with synapse function (Chidambaram *et al.*, 2019). BIN1 knockdown caused an increased abundance of spines with a larger head and neck diameter and a higher ratio of mature mushroom spines to immature filopodia. Although, in these experiments, we transduced cells with BIN1 shRNA at DIV5, a time at which neurons and synapses are still developing, Sartori *et al.* (2019) have reported that BIN1 is not expressed *in vitro* until DIV14. Therefore, the alterations we observe in synapse morphology are not expected to have occurred as a result of any developmental roles of BIN1. In support of this, BIN1 over-expression in mice results in the opposite changes to spines, leading to structural alterations in the hippocampus and memory deficits (Daudin *et al.*, 2018). Mushroom spines are considered to be more stable and in general, as spine size increases the number of AMPA receptors on the spine increases, promoting synaptic strength (Hanley, 2008; Lee *et al.*, 2012; Woolfrey and Srivastava, 2016). This may appear to be in contrast with our assertion that BIN1 knockdown promotes synapse damage by directing phosphorylated tau into synapses. However, synapse enlargement is reported to occur in the early stages of neurodegeneration in Alzheimer’s disease (DeKosky and Scheff, 1990) as a compensatory mechanism for the pre-synapse loss occurring early in the disease process. A similar mechanism at the post-synapse could explain the increased spine diameter we report here. It is also possible that the effect of altering BIN1 expression on dendritic spines is independent of its binding to tau. Schurmann *et al.* (2019) report that BIN1 modulates vesicle trafficking from recycling endosomes to the cell surface, thereby altering the surface localization of AMPA receptors at the post-synapse. Hence, alterations in vesicle trafficking may be another mechanism by which BIN1 alters the structure of dendritic spines.

Finally, we demonstrate that reducing *BIN1* expression reduced tau secretion from neurons, both in basal conditions and following neuronal depolarization. We previously showed that tau released from primary neurons is largely intact, dephosphorylated (Pooler *et al.*, 2012), and others have shown that this form of extracellular tau has important trans-cellular signalling functions (Gomez-Ramos *et al.*, 2008) distinguishing it from the aggregated, cleaved and highly phosphorylated tau species implicated in trans-synaptic tau spread/propagation (Wu *et al.*, 2016). Our results therefore suggest that BIN1 loss in Alzheimer’s disease could reduce the availability of extracellular tau, resulting in a loss of extracellular tau function.

In conclusion, our data demonstrate that BIN1 binds in a tau phosphorylation-dependent manner to P216 of tau. We find that BIN1 is lost in Alzheimer’s disease cytoplasm and that this correlates with tau accumulation in synapses and its loss from the cytoplasm. Modelling the loss of BIN1 in Alzheimer’s disease in primary neurons showed that when BIN1 is knocked down, phosphorylated tau accumulates at synapses. We also see that BIN1 loss causes alterations to synapse structure and disrupts tau release. We hypothesize that disruptions to BIN1 proteins in Alzheimer’s disease affect normal tau functions in the extracellular space and promote phosphorylated tau-mediated synaptotoxicity. These data provide a potential mechanism by which polymorphisms near *BIN1* may increase Alzheimer’s disease risk.

## Supplementary Material

fcaa011_Supplementary_Data

## Abbreviations

AMPA =: α-amino-3-hydroxy-5-methyl-4-isoxazolepropionic acid
BIN1 =: Bridging integrator 1
DIV =: days *in vitro*
GST =: glutathione-*S*-transferase
SH3 =: src homology 3
shRNA =: short hairpin RNA
