# Supplementary material for: Bridging integrator 1 protein loss in Alzheimer’s disease promotes synaptic tau accumulation and disrupts tau release
Source: Brain Commun. 2020 Feb 14;2(1):fcaa011. doi: 10.1093/braincomms/fcaa011 (PMC7272218; doi:10.1093/braincomms/fcaa011)
Supplement: fcaa011_Supplementary_Data [file fcaa011_supplementary_data.zip › Original_submission.pdf]

OXFORD UNIVERSITY PRESS | Brain Communications

Loss of BIN1 protein in Alzheimer's disease promotes synaptic accumulation of phosphorylated tau and disrupts tau release

Journal:	*Brain Communications*
Manuscript ID	BRAINCOM-2019-115
Manuscript Type:	Original Article
Date Submitted by the Author:	06-Sep-2019

Complete List of Authors:	Glennon, Elizabeth; King's College London, Basic and Clinical Neuroscience<br>Lau, Dawn; King's College London, Basic and Clinical Neuroscience<br>Gabriele, Rebecca; King's College London, Basic and Clinical Neuroscience<br>Taylor, Matthew; King's College London, Basic and Clinical Neuroscience<br>Troakes, Claire; King's College London, Basic and Clinical Neuroscience<br>Opie-Martin, Sarah; King's College London, Basic and Clinical Neuroscience<br>Elliot, Christina; King's College London, Basic and Clinical Neuroscience<br>Killick, Richard; King's College London, Basic and Clinical Neuroscience<br>Hanger, Diane; King's College London, Basic and Clinical Neuroscience<br>Gomez Perez-Nievas, Beatriz; King's College London, Basic and Clinical Neuroscience<br>Noble, Wendy; King's College London, Basic and Clinical Neuroscience

Keywords:	Alzheimer's Disease, BIN1, Tau, GWAS, Synapse, Missorting

SCHOLARONE™ Manuscripts

Loss of BIN1 protein in Alzheimer's disease promotes synaptic accumulation of phosphorylated tau and disrupts tau release

Elizabeth B. Glennon[1]*, Dawn H-W Lau[1], Rebecca M.C. Gabriele[1], Matthew F. Taylor[1], Claire Troakes[1], Sarah Opie-Martin[1], Christina Elliott[1], Richard Killick[1], Diane P. Hanger[1], Beatriz G. Perez-Nievas[1], Wendy Noble[1]*

[1]King's College London, Institute of Psychiatry, Psychology and Neuroscience, Department of Basic and Clinical Neuroscience, Maurice Wohl Clinical Neuroscience Institute, 5 Cutcombe Road, London, SE5 9RX. UK. [2]King's College London, MRC London Neurodegenerative Diseases Brain Bank, London, UK.

*Dr Elizabeth Glennon, King's College London, Institute of Psychiatry, Psychology and Neuroscience, Department of Basic and Clinical Neuroscience, Maurice Wohl Clinical Neuroscience Institute, 5 Cutcombe Road, London, SE5 9RX. UK.

Tel +44 (0)20 7848 0090, Fax: +44(0)20 7708 0017

Email: elizabeth.glennon@kcl.ac.uk

*Dr Wendy Noble,

King's College London, Institute of Psychiatry, Psychology and Neuroscience, Department of Basic and Clinical Neuroscience, Maurice Wohl Clinical Neuroscience Institute, Rm1.23, 5 Cutcombe Road, London, SE5 9RX. UK.

Tel +44 (0)20 7848 0578, Fax: +44(0)20 7708 0017,

Email: wendy.noble@kcl.ac.uk

Running title: BIN1 loss affects tau trafficking and release

For Review Only

Author Contributions

EBG performed most experiments and EBG and SO-M analysed data; MFT, DHWL, CT, RMCG, CE, RK, DPH and BP-N performed additional experiments, and provided expertise and advice. EBG and WN designed the research, wrote and revised the paper.

This PDF file includes:

Main Text

Figures 1 to 6

Tables 1 to 2

Supplementary Data

For Review Only

Abstract

Polymorphisms associated with BIN1 confer the second greatest risk for developing late onset Alzheimer's disease. The biological consequences of this genetic variation are not fully understood, however BIN1 is a binding partner for tau. Tau is normally a highly soluble cytoplasmic protein, but in Alzheimer's disease tau is abnormally phosphorylated and accumulates at synapses to exert synaptotoxicity. We show here that BIN1 is lost from the cytoplasmic fraction of Alzheimer's disease cortex, and this is accompanied by the progressive mislocalization of phosphorylated tau to synapses. We confirmed proline 216 in tau as critical for tau interaction with the BIN1-SH3 domain and show that phosphorylation of tau disrupts this interaction, suggesting that tau-BIN1 associations may be disrupted in Alzheimer's disease when tau is highly phosphorylated. Moreover, mimicking the loss of BIN1 that we find in Alzheimer's disease brain by BIN1 knockdown in neurons led to the damaging accumulation of phosphorylated tau at synapses. We also observed alterations in dendritic spine morphology indicative of altered synapse function, and reduced release of tau from neurons upon BIN1 silencing, suggesting that BIN1 loss disrupts the function of extracellular tau. Together, these data indicate that polymorphisms associated with BIN1 that reduce BIN1 protein levels in the brain likely act synergistically with increased tau phosphorylation to increase risk of Alzheimer's disease by disrupting cytoplasmic tau-BIN1 interactions, promoting the damaging mis-sorting of phosphorylated tau to synapses to alter synapse structure, and by reducing the release of physiological forms of tau to disrupt tau function.

Keywords

Alzheimer's Disease, BIN1, GWAS, Tau, Synapse

For Review Only

Main Text

Introduction

Tauopathies including Alzheimer's disease are characterized by tau protein modifications that affect normal tau interactions and localization, and the development of neurofibrillary pathology (Guo, Noble, and Hanger 2017). The redistribution of highly phosphorylated and/or oligomeric tau species to pre- and post-synapses causes disruption of synaptic vesicle mobility and neurotransmitter release (McInnes et al. 2018; Zhou et al. 2017), and excitotoxicity (Ittner et al. 2010; Li and Gotz 2017), respectively. As a result, the accumulation of phosphorylated tau at synapses is closely linked with synapse loss and dementia in Alzheimer's disease (Perez-Nievas et al. 2013; Hanseeuw et al. 2019). Developing a better understanding of the causes of tau protein redistribution to synapses may elucidate potential new treatment strategies for Alzheimer's disease and related tauopathies.

Recent genome-wide association studies have identified several gene variants that increase risk of developing Alzheimer's disease. Of those identified to date, polymorphisms associated with bridging integrator 1 (BIN1) confer the second largest genetic risk factor for developing sporadic Alzheimer's disease, after the apolipoprotein E4 allele (APOE4) (Hu et al. 2011; Naj et al. 2014; Seshadri et al. 2010; Vardarajan et al. 2015; Lambert et al. 2013; Wijsman et al. 2011). Rare variants in coding regions of BIN1 have been identified (Vardarajan et al. 2015), however the more common *BIN1* variants are upstream of the gene and do not affect protein structure. However, these may affect tissue-specific splicing or expression of the cytoplasmic membrane-binding BIN1 protein which is known to play important roles in endocytosis and subcellular trafficking (Prokic, Cowling, and Laporte 2014). In support of this, expression of

For Review Only

the longer neuronal isoforms of BIN1 are decreased and the shorter glial isoforms are increased in Alzheimer's disease brain (De Rossi et al. 2016; Glennon et al. 2013; Holler et al. 2014).

Whilst most genetic variants that increase risk of Alzheimer's disease affect β-amyloid generation and/or clearance, BIN1 is relatively unusual in that its effects in Alzheimer's disease appear to be mediated by tau (Chapuis et al. 2013; Wang et al. 2016). In Alzheimer's disease brain, BIN1 may colocalize with neurofibrillary tangle-containing neurons (Holler et al. 2014), and is associated with elevated tau phosphorylation (Wang et al. 2016). Expression of BIN1 in a Drosophila model of Alzheimer's disease was shown to modulate the toxicity of tau (Chapuis et al. 2013) and knockdown of BIN1 promotes tau propagation between neurons (Calafate et al. 2016). Others have shown that BIN1 over-expression in mice causes microstructural changes in hippocampal circuits (Daudin et al., 2018). These are the first circuits to show tau pathology in Alzheimer's disease (Daudin et al., 2018), suggesting that BIN1 may affect the development of Alzheimer's disease by modulating tau effects at synapses, and possibly also synaptic activity-dependent tau release (Pooler et al. 2013)(23).

The effects of BIN1 on tau appear to be mediated by direct association between the two proteins. Interactions between BIN1 and tau have been demonstrated in cell models, drosophila and mice (Chapuis et al. 2013; Sottejeau et al. 2015; Malki et al. 2017; Sartori et al. 2019). BIN1 contains a src homology 3 (SH3) domain through which it interacts with prolines (P) within PXXP motifs (Prokic, Cowling, and Laporte 2014). Tau contains seven PXXP motifs in its proline-rich domain (Usardi et al. 2011), and the SH3 domain of BIN1 interacts with the proline rich region of tau in a phosphorylation-dependent manner (Chapuis et al. 2013; Sottejeau et al. 2015; Malki et al. 2017; Sartori et al. 2019). However, the mechanisms by which BIN1 affects tau to mediate pathological changes in tau proteins are not fully understood. The purpose of this study was to determine if alterations to BIN1 and tau in

For Review Only

Alzheimer's disease promote the damaging redistribution of tau to synapses, as a mechanism by which BIN1 polymorphisms may increase risk of developing Alzheimer's disease.

Materials and Methods

Human Brain

Braak staged post-mortem human temporal cortex tissue was obtained from the Medical Research Council London Neurodegenerative Diseases Brain Bank at King's College London following ethical approval (Research Ethics Committee reference: 08/MRE09/38 + 5). Neuropathological assessment was performed according to standard criteria. Samples were classified as control (no history of neurodegenerative or psychiatric disease and age-related pathology only), moderate Alzheimer's disease (clinical diagnosis of Alzheimer's disease and pathology reaching Braak stage III-IV) and severe Alzheimer's disease (clinical diagnosis of Alzheimer's disease and pathology reaching Braak stage V-VI). Characteristics of these samples are summarized in Tables 1 and 2.

For Review Only

Modification of BIN1 expression in primary neurons

Primary cortical neurons dissected from embryonic day (E)18 Sprague-Dawley rats were cultured as previously described (Pooler et al. 2012) on poly-D-lysine coated plates or glass cover-slips. Lentivirus shRNA targeting BIN1 from the RNAi consortium (TRCN0000088188), and a scrambled control sequence in the pLKO.1 vector, were purchased from Dharmacon Horizon (CO, USA). PAX2 and pMG.2 lentiviral packaging vectors were kind gifts from Dr Maria Jimenez-Sanchez (King's College London). Human embryonic kidney (HEK293) cells cultured in Dulbecco's modified eagle medium plus GlutaMAX

(DMEM, Thermo Fisher Scientific, MA, USA) supplemented with 10 % (v/v) fetal bovine serum (FBS, Thermo Fisher Scientific) were transfected with PAX2, pMG.2, and shRNA lentivirus using lipofectamine 2000 (Invitrogen, CA, USA). After 24 hours, lentiviral particles were collected from culture medium, isolated and concentrated according to the manufacturer's instructions. For lentiviral knockdown, neurons were cultured for 5 DIV, then treated with either BIN1 targeting shRNA, scrambled or control shRNA lentiviral particles for 24 hours, after which time the virus was removed and neurons further cultured until 21-23 days *in vitro* (DIV) prior to use. Alternatively, BIN1 was knocked down in primary neurons using Accell BIN1 siRNA smart pool (E-095528) purchased from Dharmacon Horizon Discovery UK. At 19 DIV, rat primary cortical neurons were transfected with 50 nM BIN1 or non-targeting control siRNA (Dharmacon Horizon Discovery) using lipofectamine 2000 for 96 hours at 37°C, after which time neurons were imaged or harvested.

For Review Only

Tau enzyme-linked immunosorbent assay (ELISA) and cell viability assays

The tau ELISA was performed on HBSS without with Ca^{2+} and Mg^{2+} incubated for 4 hours with 22-23 DIV primary neurons as we described previously (Croft et al. 2017). Lactate dehydrogenase activity in the media of cultured neurons was determined using an LDH cytotoxicity kit from Thermo Fischer Scientific according to the manufacturer's instructions.

Immunofluorescence

Immunofluorescence was performed as described (Schurmann et al. 2019), using 2 % (v/v) fetal bovine serum (FBS, Life Technologies) in place of normal goat serum. Cells were incubated with primary antibodies against BIN1 (ab54764, Abcam), post-synaptic density 95

(PSD-95, D74D3, Cell Signaling), synaptophysin (sc7568, Santa Cruz, TX, USA) and microtubule associated protein 2 (MAP2, GTX82661, GeneTex), and the appropriate species of alexafluor-conjugated secondary antibodies (Life Technologies). Coverslips mounted onto glass slides using pro-long diamond mounting media (Life Technologies). Labelled proteins were imaged using an Eclipse Ti2 inverted Nikon 3D structured illumination microscope (N-SIM) and images reconstructed using Nikon Imaging Systems Elements software, or a Nikon Eclipse Ti-2 inverted microscope with Vt-iSIM scan head and deconvolved using Nikon Imaging Systems Elements software.

For Review Only

Analysis of synapses

Neurons were fractionated to generate cytosol- and synapse- enriched fractions using a protocol modified from (Frandemiche et al. 2014). Total, cytosolic and synaptoneurosome fractions were isolated from post-mortem temporal cortex as described by us previously (Perez-Nievas et al. 2013). Equal protein amounts of total, synaptic and cytoplasmic fractions were immunoblotted.

For analysis of dendritic spine structure, neurons at 22 DIV were transfected with enhanced green fluorescent protein (GFP) eGFP-N2 plasmid (Clontech, Kyoto, Japan) using lipofectamine 2000 for 24 hours, fixed and the GFP signal imaged using a Nikon Eclipse Ti-2 inverted microscope with Vt-iSIM scan head. 3x3 large image stacks were acquired covering the entire volume of the neuron, with 0.2 μm between each image in the Z plane. Neurolucida™ software (MBF Bioscience, VT, USA) was used to trace neurons and detect, classify and quantify dendritic spines and perform the Scholl analysis. Neuronal complexity was determined by (sum of the terminal orders + number of terminals) * (total dendritic length /

number of primary dendrites), where terminals is the number of branch endings, and terminal orders is the number of branches between the terminal and the cell body.

GST binding assays

BIN1-SH3 cDNA generously provided by Isabelle Landrieu (University of Lille Nord de France) was cloned into pGEX5X1 using sequence and ligation independent cloning (SLIC) (Hill and Eaton-Rye 2014). The BIN1-SH3 domain was amplified from the original vector using primers 5'-TCG AGC GGC CGC ATC GTG ACA TGG GTC GTC TGG ATC TG-3' and 5'-AAA CGC GCG AGG CAG ATC GTC AGT TAC GGC ACA CGC TCA GTA AAA TTC-3', and pGEX5X1 was linearized using primers 5'-CTG ACG ATC TGC CTC GCG-3' and 5'-GTC ACG ATG CGG CCG CTC-3'. SLIC products were used to transform BL21 *E.coli* (New England Biolabs, MA, USA) by heat shock. DNA was purified using QIAgen spin miniprep kit (QIAgen, Hilden, Germany), and the cloning was confirmed by sequencing (Source Bioscience, Nottingham, UK, using stock primers to GST plasmid). BL21 *E.coli* containing either BIN1-SH3-pGEX5X1 or empty vector pGEX5X1 were used to produce glutathione S-transferase (GST) fusion proteins, and GST-pulldown assays were performed as described previously (Lau et al. 2016). Wild type (WT) human 2N4R tau and PxxP mutant tau plasmids have been described previously (Lau et al. 2016). These were expressed in HEK293 cells for 24 hours after which time, cells were lysed and the lysates used for GST pull-downs, which were performed as we described before (Lau et al. 2016).

SDS-PAGE and western blotting

For Review Only

Samples were electophoresed on 10 % tris-glycine-SDS-polyacrylamide gels, Nu-Page 4-12 % or 10 % bis-tris gels (Invitrogen), transferred to 0.45 µm nitrocellulose membrane (Millipore, MA, USA), and immunoblotted using standard methods. Primary antibodies were BIN1 (99D, Millipore), glutathione S-transferase (GST) (GE Healthcare, IL, USA), total tau (total human tau, Agilent), Tau-1 (Millipore), PHF1 (Peter Davies, Donald and Barbara Zucker School of Medicine at Hofstra, Northwell), N-methyl-D-aspartate subunit 2A (NR2A) (07-632, Millipore), β-actin (ac15, abcam), synaptophysin (sc17750, Santa Cruz), and PSD95 (MAB 1596, Millipore). Bound horseradish peroxidise (HRP)-conjugated secondary antibodies (GE Healthcare) were detected using enhanced chemiluminesence solutions (Thermo Fisher Scientific) and visualized using a chemi-doc imager (Bio-rad, CA, USA). Densitometric analysis was performed using FIJI.

Data analysis and statistics

Statistical tests were performed using GraphPad Prism 7.0 (CA, USA) or RStudio. Normality tests were performed on all data, and the appropriate statistical tests were then used to determine differences between experimental groups. Tests used and n numbers are provided for each experiment in the figure legends.

For Review Only

Data Availability

The data supporting this study are available in the manuscript and supplementary material

Results

BIN1 loss in cytoplasmic fractions correlates with increased synaptic tau in Alzheimer's disease brain

Early in Alzheimer's disease, highly phosphorylated tau is mislocalized to synaptic compartments (Perez-Nievas et al. 2013) where tau disrupts synapse function and mediates synaptotoxicity (Ittner et al. 2010; Li and Gotz 2017; McInnes et al. 2018; Zhou et al. 2017). The longest neuronal isoform of BIN1 protein is reduced in end-stage Alzheimer's disease brain (De Rossi et al. 2017; Glennon et al. 2013). To determine if BIN1 is lost at earlier stages of Alzheimer's disease, and if its loss is associated with changes in the distribution of tau, we isolated synaptoneurosomes (Perez-Nievas et al. 2013) from control (Braak stage 0-III), moderate (Braak stage III-IV) and severe (Braak stage V-VI) post-mortem Alzheimer's disease temporal cortex and examined total, cytosolic and synaptic fractions on western blots. The integrity of synaptic proteins in these samples was first confirmed, as previously described (Hesse et al., 2019), by western blotting a subset of samples with an antibody against NR2A (Supplementary Fig.1).

In total brain homogenates, we confirmed a trend towards reduction of BIN1 in severe relative to moderate Alzheimer's disease and control tissues (Figure 1A, B), and significant increases in total tau amounts with increasing disease severity (Figure 1A, C), as previously reported (Kurbatskaya et al. 2016). Protein amounts were normalized to neuron-specific enolase in the same sample prior to quantification to control for any effects of neuronal loss and/or gliosis

For Review Only

(Kurbatskaya et al. 2016). The cytoplasmic and synaptoneurosome fractions isolated from the same brain samples were characterization to confirm their purity (Supplementary Fig. 1). Blotting of these samples showed a significant accumulation of tau in the synaptic compartment in severe Alzheimer's disease, relative to moderate Alzheimer's disease and controls (Figure 1D, F). Tau phosphorylated at S396/S404 (PHF1) was found to accumulate at synapses in both moderate and Alzheimer's disease relative to controls (Figure 1D, G). The accumulation of synaptic tau paralleled the loss of cytoplasmic tau (Figure 1H, J) suggesting that these results reflect tau mis-sorting from the cytoplasm to synapses. There were no differences in BIN1 levels in synaptoneurosomes between groups (Figure 1D, E). However, we found marked and significant losses of cytoplasmic BIN1 in both moderate and severe Alzheimer's disease tissues (Figure 1H, I) that correlated positively with reductions in cytoplasmic tau (Figure 2J, 4A) and inversely with increased synaptic tau (Figure 2B, C). Taken together, these data suggest that loss of cytoplasmic BIN1 may facilitate the mis-sorting of phosphorylated tau to the synapse.

For Review Only

BIN1 knockdown causes synaptic accumulation of phosphorylated tau in neurons

To test if BIN1 loss induces tau mislocalization to synapses, we silenced BIN1 in rat primary cortical neurons using lentivirus. We confirmed the efficiency of BIN1 knockdown by western blotting (Supplementary Fig. 2A) and proximity ligation assay (PLA) (Supplementary Fig. 2G). In control neurons, BIN1 and tau are found in cytoplasmic and synaptic fractions (Fig. 3A; Supplementary Fig. 2G). N-SIM imaging of neuronal processes showed that BIN1 decorates MAP2-positive and MAP2-negative fibers (Figure 3B) and localizes in close proximity to pre-synaptic (synaptophysin) and post-synaptic (PSD95) markers, with a portion of BIN1 co-localizing with PSD95 (Figure 3C). Cultures were also immunolabelled with an

antibody against GFAP which suggests that a small proportion of BIN1 is astrocytic (Supplementary Fig. 2H-I), in agreement with recent reports (Taga et al., 2019).

We found that knockdown of BIN1 did not alter the total amount of tau or its phosphorylation in total cell lysates (Supplementary Fig. 2). However, following BIN1 knockdown there was a significant increase in the amounts of phosphorylated tau in synaptic fractions relative to controls (Figure 3D, F). These data show that reducing BIN1 in neurons causes the accumulation of phosphorylated tau at synapses. These data suggest that the increased synaptic phospho-tau we observe in Alzheimer's disease brain may result from the loss of cytoplasmic BIN1.

The interaction of BIN1 with tau is reported to be regulated by tau phosphorylation via direct association of the BIN1-SH3 domain and the proline-rich region of tau. To confirm this, we generated GST-BIN1-SH3 constructs (Supplementary Fig. 3A) and we used these and GST-only constructs in binding assays using lysates from HEK293 cells transfected with wild-type human 2N4R tau, mutant tau constructs or empty vector. The mutant tau constructs are human 2N4R tau in which a single proline (P) in each PXXP motif is mutated to alanine (Supplementary Fig. 3B), as we reported previously (Lau et al. 2016). Immunoblotting of BIN1-SH3-GST pull-downs with an antibody against tau confirmed that the BIN1-SH3 domain binds human 2N4R tau (Supplementary Fig. 3A). Analysis of BIN1-SH3 binding to mutant tau constructs showed that P216 is important for the tau-BIN1 interaction since the amount of P216A tau bound to BIN1-SH3 was significantly decreased relative to WT tau (Fig. 4A, B). There were no significant differences in tau binding to BIN1-SH3 when any other proline residue was mutated to alanine.

To confirm that increasing tau phosphorylation, to mimic tau modifications in Alzheimer's disease, affects the interaction of tau with BIN1 in rat primary cortical cultures, cells were

For Review Only

treated for 4 hours with 50 nM okadaic acid (OA), a protein phosphatase inhibitor that prevents removal of phosphate residues throughout the tau molecule (Pooler et al. 2012; Van Dolah and Ramsdell 1992) (Fig. 4D). GST pulldowns with lysates from these cells confirmed that phosphorylated tau shows only trace amounts of binding to BIN1-SH3-GST when compared to lysates from vehicle-treated cells (Figure 4E). When tau was dephosphorylated upon application of 25 mM of the glycogen synthase kinase-3 (GSK3) inhibitor lithium chloride (LiCl) (Stambolic, Ruel, and Woodgett 1996; Pooler et al. 2012) (Figure 4F-G), or 20 μM of the casein kinase-1 (CK1) inhibitor IC261 (Pooler et al. 2012) (Supplementary Fig. 4), the amount of tau pulled down by BIN1-SH3 was similar to controls. GSK3 and CK1 inhibitors were used to modulate tau phosphorylation since these kinases phosphorylate tau throughout the protein (Guo, Noble, and Hanger 2017), and phosphorylation of tau sites distal and proximal to P216 mediate tau interactions with BIN1-SH3 (Sottejeau et al. 2015; Lasorsa et al. 2018). These data confirm and extend previous findings to show that BIN1-SH3 interacts with P216 in tau, predominantly when tau is dephosphorylated. Taken together, our data suggest that tau phosphorylation in Alzheimer's disease disrupts the BIN1-tau interaction and allows tau to mislocalize to synapses.

For Review Only

Loss of BIN1 alters spine morphology and reduces tau release from neurons

Synaptic tau is closely linked with the propagation of tau pathology in Alzheimer's disease and related tauopathies (Guo, Noble, and Hanger 2017; Yamada 2017). In addition, the release of soluble tau species in physiological conditions allows important signaling roles of extracellular tau (Pooler et al. 2013; Gomez-Ramos et al. 2008), and this tau function may be lost in Alzheimer's disease (Croft et al. 2017).

Tau release is predominantly mediated by synaptic activity and modulating BIN1 expression affects dendritic spine morphology and α-amino-3-hydroxy-5-methyl-4-isoxazolepropionic acid (AMPA) receptor-mediated synaptic transmission via changes in AMPA receptor surface expression and trafficking (Daudin et al., 2018; (Schurmann et al. 2019). Since we and others have previously shown that neuronal depolarization and stimulation of AMPA receptors mediates endogenous tau release (Croft et al. 2017; Pooler et al. 2013), we examined the effects of BIN1 knockdown on synapse morphology and tau release. Examination of neurons by iSIM showed that BIN1 knockdown affects synaptic morphology in 23 DIV primary neurons exogenously expressing eGFP. BIN1 knockdown did not cause any alterations in dendritic spine length, volume or density (Figure 5A-D), but resulted in significant increases in the diameter of spine heads and necks (Figure 5E-F) and a reduced head:neck diameter ratio (Figure 5G). When spine morphologies were examined, BIN1 knockdown had no effect on the proportion of immature stubby or thin spines (Figure 6H,I), but significantly reduced the proportion of filopodia (Figure 5K) and increased the proportion of mature mushroom spines (Figure 6J), which are relatively stable and have a high density of AMPA receptors (Hanley 2008; Lee, Soares, and Beique 2012; Woolfrey and Srivastava 2016). The structure and density of dendritic spines varies according to branch order of the neurite, and Scholl analysis (Supplementary Fig. 5) showed that BIN1 knockdown increases branching of dendrites and increases neuronal complexity which may contribute to the difference in spine structure we observe since spines on all branches of neurites were quantified.

To determine if BIN1 knockdown affects tau release, tau content in culture medium from 21 DIV neurons treated with BIN1 and control siRNAs was measured by ELISA and normalized to the total amount of tau in neurons from the same culture well, as we reported previously (Croft et al. 2017). BIN1 knockdown caused a significant reduction in basal tau release without affecting the amount of intracellular tau (Figure 6A-C). BIN1 knockdown followed by

For Review Only

depolarization of neurons with potassium chloride (KCl) to stimulate tau release (Croft et al. 2017; Pooler et al. 2013), also significantly reduced tau release relative to controls (Figure 6D-F). The observed changes in tau release were not due to cell toxicity since there were no alterations in lactate dehydrogenase content in medium between conditions (Figure 6G). Thus, BIN1 knockdown results in alterations in synapse morphology and reduces basal and stimulated tau release. Taken together, our findings suggest that BIN1 knockdown affects tau release and thereby functions of extracellular tau, and in Alzheimer's disease -like conditions allows phosphorylated tau to mislocalised to synapses where it can exert toxicity. Our data provide novel insights into the mechanisms by which BIN1 polymorphisms may increase risk of Alzheimer's disease.

For Review Only

Discussion

BIN1 is closely linked with tau abnormalities that underlie the progression of sporadic Alzheimer's disease (Calafate et al. 2016; Sartori et al. 2019; Chapuis et al. 2013; Dourlen et al. 2019). Our results suggest that BIN1 and tau interact predominantly when tau is dephosphorylated in the cytoplasm and that altered tau phosphorylation, together with BIN1 loss in Alzheimer's disease, allows tau to be mislocalised to synapses where it can exert toxic effects. In support of this we found that P216 of tau interacts with the BIN1-SH3 and that interactions between these two proteins are disrupted when tau phosphorylation is increased in primary neurons. In addition, loss of BIN1 from Alzheimer's disease cytoplasm at moderate and severe stages of disease was accompanied by increased phosphorylated tau in synaptic compartments. To directly ascertain the effects of BIN1 loss on tau localization, we knocked down BIN1 in rat primary neurons and showed that this caused an accumulation of phosphorylated tau at synapses. In agreement with this finding, over-expression of BIN1 in

mice expressing human tau led to a reduced number of somatodendritic tau inclusions (26). This is an important concept since alterations in the trafficking and normal positioning of tau are considered to be early pathogenic changes in Alzheimer's disease (Guo, Noble, and Hanger 2017; Zempel and Mandelkow 2014; Zempel and Mandelkow 2015).

We also investigated the effects of BIN1 knockdown on synapse structure and tau release, since in addition to promoting the spread of tau pathology (Yamada 2017), synapse function is also important for the release of tau and its extracellular signaling activity (Gomez-Ramos et al. 2008). We show here that BIN1 knockdown affects the morphology of dendritic spines, a feature closely linked with synapse function (Chidambaram et al. 2019). BIN1 knockdown caused an increased abundance of spines with a larger head and neck diameter, and a higher ratio of mature mushroom spines to immature filopodia. In agreement with this, BIN1 over-expression in mice results in the opposite changes to spines, leading to structural alterations in the hippocampus and memory deficits (Daudin et al., 2018). Mushroom spines are considered to be more stable and in general as spine size increases the number of AMPA receptors on the spine increases, promoting synaptic strength Hanley, 2008; Lee et al., 2012; Woolfrey and Srivastava, 2016). This may appear to be in contrast with our assertion that BIN1 knockdown promotes synapse damage by directing phosphorylated tau into synapses. However, synapse enlargement is reported to occur in the early stages of neurodegeneration in Alzheimer's disease (DeKosky and Scheff 1990) as a compensatory mechanism for the pre-synapse loss occurring early in the disease process, and a similar mechanism at the post-synapse could explain the increased spine diameter we report here. It is also possible that the effect of altering BIN1 expression on dendritic spines is independent of its binding to tau. Schurmann et al. (2019) report that BIN1 modulates vesicle trafficking from recycling endosomes to the cell surface thereby altering the surface localization of AMPA receptors at the post-synapse. Hence

For Review Only

altered in vesicle trafficking may be another mechanism by which BIN1 alters the structure of dendritic spines.

Finally, we demonstrate that reducing BIN1 expression caused reduced tau secretion from neurons, both in basal conditions and following neuronal depolarization. We previously showed that tau released from primary neurons is largely intact, dephosphorylated, and likely to be important for trans-cellular signaling functions of extracellular tau (Gomez-Ramos et al. 2008) distinguishing it from the aggregated, cleaved and highly phosphorylated tau species implicated in trans-synaptic tau spread/propagation (Wu et al. 2016). Our results therefore suggest that BIN1 loss in Alzheimer's disease could reduce the availability of extracellular tau, resulting in a loss of extracellular tau function.

In conclusion, our data demonstrate that BIN1 binds in a tau phosphorylation-dependent manner to P216 of tau. We find that BIN1 is lost in Alzheimer's disease cytoplasm, and that this correlates with tau accumulation in synapses and its loss from the cytoplasm. Modelling the loss of BIN1 in Alzheimer's disease in primary neurons showed that when BIN1 is knocked down, phosphorylated tau accumulates at synapses. We also see that BIN1 loss causes alterations to synapse structure and disrupts tau release. We hypothesize that disruptions to BIN1 proteins in Alzheimer's disease affect normal tau functions in the extracellular space and promote phosphorylated tau-mediated synaptotoxicity. These data provide a potential mechanism by which polymorphisms near BIN1 may increase Alzheimer's disease risk.

For Review Only

Acknowledgments

We are grateful to Professor Isabelle Landrieu (University of Lille Nord de France) for her generous gift of BIN1-SH3 domain plasmid, and Professor Peter Davies (Feinstein Institute of Medical Research, NY, USA) for his kind gift of tau antibodies.

Funding

This work was supported by Alzheimer's Research UK (ARUK-RF2015-5, ARUK-PhD2017-4 to EG and ARUK-RF2014-2 to BP-N and ARUK-EG2013-B1 to WN).

Competing Interests

The authors declare no competing interests

References

Calafate, S., W. Flavin, P. Verstreken, and D. Moechars. 2016. 'Loss of Bin1 Promotes the Propagation of Tau Pathology', *Cell Rep*, 17: 931-40.

Chapuis, J., F. Hansmannel, M. Gistelinck, A. Mounier, C. Van Cauwenberghe, K. V. Kolen, F. Geller, Y. Sottejeau, D. Harold, P. Dourlen, B. Grenier-Boley, Y. Kamatani, B. Delepine, F. Demiautte, D. Zelenika, N. Zommer, M. Hamdane, C. Bellenguez, J. F. Dartigues, J. J. Hauw, F. Letronne, A. M. Ayral, K. Sleegers, A. Schellens, L. V. Broeck, S. Engelborghs, P. P. De Deyn, R. Vandenberghe, M. O'Donovan, M. Owen, J. Epelbaum, M. Mercken, E. Karran, M. Bantscheff, G. Drewes, G. Joberty, D. Campion, J. N. Octave, C. Berr, M. Lathrop, P. Callaerts, D. Mann, J. Williams, L. Buee, I. Dewachter, C. Van Broeckhoven, P. Amouyel, D. Moechars, B. Dermaut, J. C. Lambert, and Gerad consortium. 2013. 'Increased expression of BIN1 mediates Alzheimer genetic risk by modulating tau pathology', *Mol Psychiatry*, 18: 1225-34.

Chidambaram, S. B., A. G. Rathipriya, S. R. Bolla, A. Bhat, B. Ray, A. M. Mahalakshmi, T. Manivasagam, A. J. Thenmozhi, M. M. Essa, G. J. Guillemin, R. Chandra, and M. K.

For Review Only

Sakharkar. 2019. 'Dendritic spines: Revisiting the physiological role', *Prog Neuropsychopharmacol Biol Psychiatry*, 92: 161-93.

Croft, C. L., M. A. Wade, K. Kurbatskaya, P. Mastrandreas, M. M. Hughes, E. C. Phillips, A. M. Pooler, M. S. Perkinton, D. P. Hanger, and W. Noble. 2017. 'Membrane association and release of wild-type and pathological tau from organotypic brain slice cultures', *Cell Death Dis*, 8: e2671.

Daudin R, Marechal D, Wang Q, Abe Y, Bourg N, Sartori M, et al. 2018. BIN1 genetic risk factor for Alzheimer is sufficient to induce early structural tract alterations in entorhinal cortex-dentate gyrus pathway and related hippocampal multi-scale impairments. *bioRxiv*.437228.

De Rossi, P., V. Buggia-Prevot, R. J. Andrew, S. V. Krause, E. Woo, P. T. Nelson, P. Pytel, and G. Thinakaran. 2017. 'BIN1 localization is distinct from Tau tangles in Alzheimer's disease', *Matters (Zur)*, 2017.

De Rossi, P., V. Buggia-Prevot, B. L. Clayton, J. B. Vasquez, C. van Sanford, R. J. Andrew, R. Lesnick, A. Botte, C. Deyts, S. Salem, E. Rao, R. C. Rice, A. Parent, S. Kar, B. Popko, P. Pytel, S. Estus, and G. Thinakaran. 2016. 'Predominant expression of Alzheimer's disease-associated BIN1 in mature oligodendrocytes and localization to white matter tracts', *Mol Neurodegener*, 11: 59.

DeKosky, S. T., and S. W. Scheff. 1990. 'Synapse loss in frontal cortex biopsies in Alzheimer's disease: correlation with cognitive severity', *Ann Neurol*, 27: 457-64.

Dourlen, P., D. Kilinc, N. Malmanche, J. Chapuis, and J. C. Lambert. 2019. 'The new genetic landscape of Alzheimer's disease: from amyloid cascade to genetically driven synaptic failure hypothesis?', *Acta Neuropathol*, 138: 221-36.

Frandemiche, M. L., S. De Seranno, T. Rush, E. Borel, A. Elie, I. Arnal, F. Lante, and A. Buisson. 2014. 'Activity-dependent tau protein translocation to excitatory synapse is disrupted by exposure to amyloid-beta oligomers', *J Neurosci*, 34: 6084-97.

Glennon, E. B., I. J. Whitehouse, J. S. Miners, P. G. Kehoe, S. Love, K. A. Kellett, and N. M. Hooper. 2013. 'BIN1 is decreased in sporadic but not familial Alzheimer's disease or in aging', *PLoS One*, 8: e78806.

Gomez-Ramos, A., M. Diaz-Hernandez, A. Rubio, M. T. Miras-Portugal, and J. Avila. 2008. 'Extracellular tau promotes intracellular calcium increase through M1 and M3 muscarinic receptors in neuronal cells', *Mol Cell Neurosci*, 37: 673-81.

For Review Only

Guo, T., W. Noble, and D. P. Hanger. 2017. 'Roles of tau protein in health and disease', *Acta Neuropathol*, 133: 665-704.

Hanley, J. G. 2008. 'AMPA receptor trafficking pathways and links to dendritic spine morphogenesis', *Cell Adh Migr*, 2: 276-82.

Hanseeuw, B. J., R. A. Betensky, H. I. L. Jacobs, A. P. Schultz, J. Sepulcre, J. A. Becker, D. M. O. Cosio, M. Farrell, Y. T. Quiroz, E. C. Mormino, R. F. Buckley, K. V. Papp, R. A. Amariglio, I. Dewachter, A. Ivanoiu, W. Huijbers, T. Hedden, G. A. Marshall, J. P. Chhatwal, D. M. Rentz, R. A. Sperling, and K. Johnson. 2019. 'Association of Amyloid and Tau With Cognition in Preclinical Alzheimer Disease: A Longitudinal Study', *JAMA Neurol*.

Hesse R, Hurtado ML, Jackson RJ, Eaton SL, Herrmann AG, Colom-Cadena M, et al. 2019. Comparative profiling of the synaptic proteome from Alzheimer's disease patients with focus on the APOE genotype. *bioRxiv*. 631556.

Hill, R. E., and J. J. Eaton-Rye. 2014. 'Plasmid construction by SLIC or sequence and ligation-independent cloning', *Methods Mol Biol*, 1116: 25-36.

Holler, C. J., P. R. Davis, T. L. Beckett, T. L. Platt, R. L. Webb, E. Head, and M. P. Murphy. 2014. 'Bridging integrator 1 (BIN1) protein expression increases in the Alzheimer's disease brain and correlates with neurofibrillary tangle pathology', *J Alzheimers Dis*, 42: 1221-7.

Hu, X., E. Pickering, Y. C. Liu, S. Hall, H. Fournier, E. Katz, B. Dechairo, S. John, P. Van Eerdewegh, H. Soares, and Initiative Alzheimer's Disease Neuroimaging. 2011. 'Meta-analysis for genome-wide association study identifies multiple variants at the BIN1 locus associated with late-onset Alzheimer's disease', *PLoS One*, 6: e16616.

Ittner, L. M., Y. D. Ke, F. Delerue, M. Bi, A. Gladbach, J. van Eersel, H. Wolfing, B. C. Chieng, M. J. Christie, I. A. Napier, A. Eckert, M. Staufenbiel, E. Hardeman, and J. Gotz. 2010. 'Dendritic function of tau mediates amyloid-beta toxicity in Alzheimer's disease mouse models', *Cell*, 142: 387-97.

Kurbatskaya, K., E. C. Phillips, C. L. Croft, G. Dentoni, M. M. Hughes, M. A. Wade, S. Al-Sarraj, C. Troakes, M. J. O'Neill, B. G. Perez-Nievas, D. P. Hanger, and W. Noble. 2016. 'Upregulation of calpain activity precedes tau phosphorylation and loss of synaptic proteins in Alzheimer's disease brain', *Acta Neuropathol Commun*, 4: 34.

Lambert, J. C., C. A. Ibrahim-Verbaas, D. Harold, A. C. Naj, R. Sims, C. Bellenguez, A. L. DeStafano, J. C. Bis, G. W. Beecham, B. Grenier-Boley, G. et al. 2013. 'Meta-analysis

For Review Only

of 74,046 individuals identifies 11 new susceptibility loci for Alzheimer's disease', *Nat Genet*, 45: 1452-8.

Lasorsa, A., I. Malki, F. X. Cantrelle, H. Merzougui, E. Boll, J. C. Lambert, and I. Landrieu. 2018. 'Structural Basis of Tau Interaction With BIN1 and Regulation by Tau Phosphorylation', *Front Mol Neurosci*, 11: 421.

Lau, D. H., M. Hogseth, E. C. Phillips, M. J. O'Neill, A. M. Pooler, W. Noble, and D. P. Hanger. 2016. 'Critical residues involved in tau binding to fyn: implications for tau phosphorylation in Alzheimer's disease', *Acta Neuropathol Commun*, 4: 49.

Lee, K. F., C. Soares, and J. C. Beique. 2012. 'Examining form and function of dendritic spines', *Neural Plast*, 2012: 704103.

Li, C., and J. Gotz. 2017. 'Somatodendritic accumulation of Tau in Alzheimer's disease is promoted by Fyn-mediated local protein translation', *EMBO J*, 36: 3120-38.

Malki, I., F. X. Cantrelle, Y. Sottejeau, G. Lippens, J. C. Lambert, and I. Landrieu. 2017. 'Regulation of the interaction between the neuronal BIN1 isoform 1 and Tau proteins - role of the SH3 domain', *FEBS J*, 284: 3218-29.

McInnes, J., K. Wierda, A. Snellinx, L. Bounti, Y. C. Wang, I. C. Stancu, N. Apostolo, K. Gevaert, I. Dewachter, T. L. Spires-Jones, B. De Strooper, J. De Wit, L. Zhou, and P. Verstreken. 2018. 'Synaptogyrin-3 Mediates Presynaptic Dysfunction Induced by Tau', *Neuron*, 97: 823-35 e8.

Naj, A. C., G. Jun, C. Reitz, B. W. Kunkle, W. Perry, Y. S. Park, G. W. Beecham, R. A. Rajbhandary, K. L. Hamilton-Nelson, L. S. Wang, J. S. et al. 2014. 'Effects of multiple genetic loci on age at onset in late-onset Alzheimer disease: a genome-wide association study', *JAMA Neurol*, 71: 1394-404.

Perez-Nievas, B. G., T. D. Stein, H. C. Tai, O. Dols-Icardo, T. C. Scotton, I. Barroeta-Espar, L. Fernandez-Carballo, E. L. de Munain, J. Perez, M. Marquie, A. Serrano-Pozo, M. P. Frosch, V. Lowe, J. E. Parisi, R. C. Petersen, M. D. Ikonomovic, O. L. Lopez, W. Klunk, B. T. Hyman, and T. Gomez-Isla. 2013. 'Dissecting phenotypic traits linked to human resilience to Alzheimer's pathology', *Brain*, 136: 2510-26.

Pooler, A. M., E. C. Phillips, D. H. Lau, W. Noble, and D. P. Hanger. 2013. 'Physiological release of endogenous tau is stimulated by neuronal activity', *EMBO Rep*, 14: 389-94.

Pooler, A. M., A. Usardi, C. J. Evans, K. L. Philpott, W. Noble, and D. P. Hanger. 2012. 'Dynamic association of tau with neuronal membranes is regulated by phosphorylation', *Neurobiol Aging*, 33: 431 e27-38.

For Review Only

Prokic, I., B. S. Cowling, and J. Laporte. 2014. 'Amphiphysin 2 (BIN1) in physiology and diseases', *J Mol Med (Berl)*, 92: 453-63.

Sartori, M., T. Mendes, S. Desai, A. Lasorsa, A. Herledan, N. Malmanche, P. Makinen, M. Marttinen, I. Malki, J. Chapuis, A. Flaig, A. C. Vreulx, M. Ciancia, P. Amouyel, F. Leroux, B. Deprez, F. X. Cantrelle, D. Marechal, L. Pradier, M. Hiltunen, I. Landrieu, D. Kilinc, Y. Herault, J. Laporte, and J. C. Lambert. 2019. 'BIN1 recovers tauopathy-induced long-term memory deficits in mice and interacts with Tau through Thr(348) phosphorylation', *Acta Neuropathol*.

Schurmann, B., D. P. Bermingham, K. J. Kopeikina, K. Myczek, S. Yoon, K. E. Horan, C. J. Kelly, M. D. Martin-de-Saavedra, M. P. Forrest, J. M. Fawcett-Patel, K. R. Smith, R. Gao, A. Bach, A. C. Burette, J. Z. Rappoport, R. J. Weinberg, M. Martina, and P. Penzes. 2019. 'A novel role for the late-onset Alzheimer's disease (LOAD)-associated protein Bin1 in regulating postsynaptic trafficking and glutamatergic signaling', *Mol Psychiatry*.

Seshadri, S., A. L. Fitzpatrick, M. A. Ikram, A. L. DeStefano, V. Gudnason, M. Boada, J. C. Bis, A. V. Smith, M. M. Carassquillo, J. C. Lambert, et al. 2010. 'Genome-wide analysis of genetic loci associated with Alzheimer disease', *JAMA*, 303: 1832-40.

Sottejeau, Y., A. Bretteville, F. X. Cantrelle, N. Malmanche, F. Demiaute, T. Mendes, C. Delay, H. Alves Dos Alves, A. Flaig, P. Davies, P. Dourlen, B. Dermaut, J. Laporte, P. Amouyel, G. Lippens, J. Chapuis, I. Landrieu, and J. C. Lambert. 2015. 'Tau phosphorylation regulates the interaction between BIN1's SH3 domain and Tau's proline-rich domain', *Acta Neuropathol Commun*, 3: 58.

Stambolic, V., L. Ruel, and J. R. Woodgett. 1996. 'Lithium inhibits glycogen synthase kinase-3 activity and mimics wingless signalling in intact cells', *Curr Biol*, 6: 1664-8.

Taga M, Petyuk V, White C, Marsh G, Ma Y, Klein H-U, Connor SM, Khairallah A, Olah M, Schneider J et al. 2019. BIN1 protein isoforms are differentially expressed in astrocytes, neurons, and microglia: neuronal and astrocyte BIN1 implicated in Tau pathology. bioRxiv 535682.

Usardi, A., A. M. Pooler, A. Seereeram, C. H. Reynolds, P. Derkinderen, B. Anderton, D. P. Hanger, W. Noble, and R. Williamson. 2011. 'Tyrosine phosphorylation of tau regulates its interactions with Fyn SH2 domains, but not SH3 domains, altering the cellular localization of tau', *FEBS J*, 278: 2927-37.

For Review Only

For Review Only

Van Dolah, F. M., and J. S. Ramsdell. 1992. 'Okadaic acid inhibits a protein phosphatase activity involved in formation of the mitotic spindle of GH4 rat pituitary cells', *J Cell Physiol*, 152: 190-8.

Vardarajan, B. N., M. Ghani, A. Kahn, S. Sheikh, C. Sato, S. Barral, J. H. Lee, R. Cheng, C. Reitz, R. Lantigua, D. Reyes-Dumeyer, M. Medrano, I. Z. Jimenez-Velazquez, E. Rogaeva, P. St George-Hyslop, and R. Mayeux. 2015. 'Rare coding mutations identified by sequencing of Alzheimer disease genome-wide association studies loci', *Ann Neurol*, 78: 487-98.

Wang, H. F., Y. Wan, X. K. Hao, L. Cao, X. C. Zhu, T. Jiang, M. S. Tan, L. Tan, D. Q. Zhang, L. Tan, J. T. Yu, and Alzheimer's Disease Neuroimaging Initiative. 2016. 'Bridging Integrator 1 (BIN1) Genotypes Mediate Alzheimer's Disease Risk by Altering Neuronal Degeneration', *J Alzheimers Dis*, 52: 179-90.

Wijsman, E. M., N. D. Pankratz, Y. Choi, J. H. Rothstein, K. M. Faber, R. Cheng, J. H. Lee, T. D. Bird, D. A. Bennett, R. Diaz-Arrastia, A. M. Goate, M. Farlow, B. Ghetti, R. A. Sweet, T. M. Foroud, R. Mayeux, and Nia-Load Ncrad Family Study Group. 2011. 'Genome-wide association of familial late-onset Alzheimer's disease replicates BIN1 and CLU and nominates CUGBP2 in interaction with APOE', *PLoS Genet*, 7: e1001308.

Woolfrey, K. M., and D. P. Srivastava. 2016. 'Control of Dendritic Spine Morphological and Functional Plasticity by Small GTPases', *Neural Plast*, 2016: 3025948.

Wu, J. W., S. A. Hussaini, I. M. Bastille, G. A. Rodriguez, A. Mrejeru, K. Rilett, D. W. Sanders, C. Cook, H. Fu, R. A. Boonen, M. Herman, E. Nahmani, S. Emrani, Y. H. Figueroa, M. I. Diamond, C. L. Clelland, S. Wray, and K. E. Duff. 2016. 'Neuronal activity enhances tau propagation and tau pathology in vivo', *Nat Neurosci*, 19: 1085-92.

Yamada, K. 2017. 'Extracellular Tau and Its Potential Role in the Propagation of Tau Pathology', *Front Neurosci*, 11: 667.

Zempel, H., and E. Mandelkow. 2014. 'Lost after translation: missorting of Tau protein and consequences for Alzheimer disease', *Trends Neurosci*, 37: 721-32.

Zempel, H., and E. M. Mandelkow. 2015. 'Tau missorting and spastin-induced microtubule disruption in neurodegeneration: Alzheimer Disease and Hereditary Spastic Paraplegia', *Mol Neurodegener*, 10: 68.

Zhou, L., J. McInnes, K. Wierda, M. Holt, A. G. Herrmann, R. J. Jackson, Y. C. Wang, J. Swerts, J. Beyens, K. Miskiewicz, S. Vilain, I. Dewachter, D. Moechars, B. De

Strooper, T. L. Spires-Jones, J. De Wit, and P. Verstreken. 2017. 'Tau association with synaptic vesicles causes presynaptic dysfunction', *Nat Commun*, 8: 15295.

For Review Only

Figures and Tables

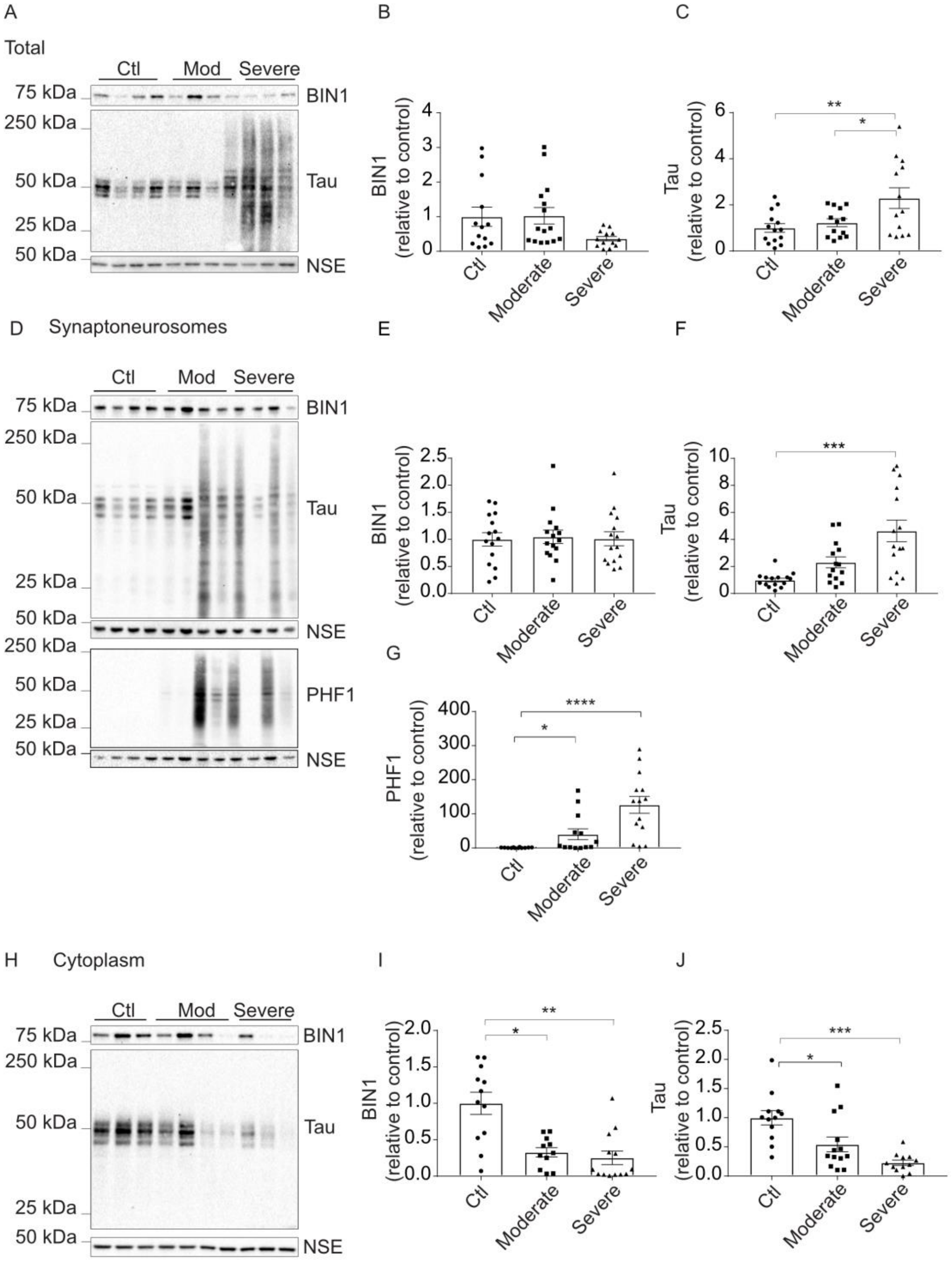

Figure 1. BIN1 and tau are lost from the cytoplasm and this is associated with accumulation of synaptic phosphorylated tau in Alzheimer's disease temporal cortex. A)

Total homogenates from temporal cortex of control (Braak stage 0-III), moderate (Braak stage III-IV) and severe (Braak stage V-VI) Alzheimer's disease brain were western blotted using antibodies against BIN1, total tau, and neuron specific enolase (NSE). Bar charts show quantification of B) BIN1 and C) tau amounts following normalisation to NSE in the same sample. Data shown are mean ± S.E.M. expressed as fold average control. Following D'Agostino and Pearson normality testing, data were analysed using a one-way ANOVA with Holm-Sidak's multiple comparisons test. n=13 per group (BIN1) or 14 per group (tau). D) Synaptoneurosomes isolated from the same temporal cortex samples were also immunoblotted with antibodies against BIN1, tau, tau phosphorylated at Ser396/404 (PHF1), and neuron-specific enolase (NSE). Bar charts show quantification of E) BIN1, F) tau, and G) PHF1 in synaptoneurosome fractions following normalisation to NSE in the same sample. Data are mean ± S.E.M. expressed as fold average control. Following D'Agostino and Pearson normality testing, data was analysed using non-parametric Kruskal-Wallis test with Dunn's multiple comparison test. n=15 per group (BIN1 and tau) or 12 per group (PHF1). H) The cytoplasmic fraction was blotted as above with antibodies against BIN1, tau, and NSE. Bar charts show quantification of I) BIN1 and J) tau in the cytoplasmic fraction following normalisation to NSE in the same sample. Data are mean ± S.E.M. expressed as fold mean control. Following D'Agostino and Pearson normality testing, BIN1 data was analysed using non-parametric Kruskal-Wallis test with Dunn's multiple comparison test and tau data using a one-way ANOVA with Holm-Sidak's multiple comparisons test. n=11 per group (BIN1) or 12 per group (tau). *$p<0.05$, ** $p<0.01$, ***$p<0.001$, ****$p<0.0001$.

For Review Only

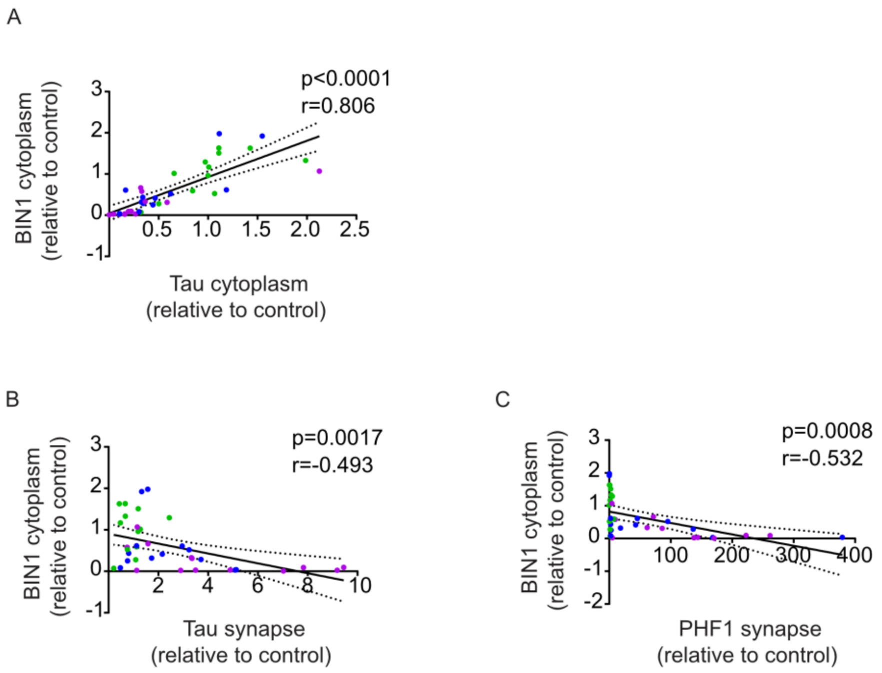

Figure 2. Loss of BIN1 correlates with loss of cytoplasmic tau and increased synaptic tau in Alzheimer's disease temporal cortex. Correlation analysis of BIN1 and tau amounts in A) cytoplasmic fractions shows a strong positive correlation between BIN1 and tau (n=38), and strong negative correlations between B) cytoplasmic BIN1 and synaptic tau (n=38), and C) cytoplasmic BIN1 and synaptic tau phosphorylated at Ser396/404 (PHF1) (n=36). Colours represent control (green), moderate (blue) and severe (purple) Alzheimer's disease Braak stage samples.

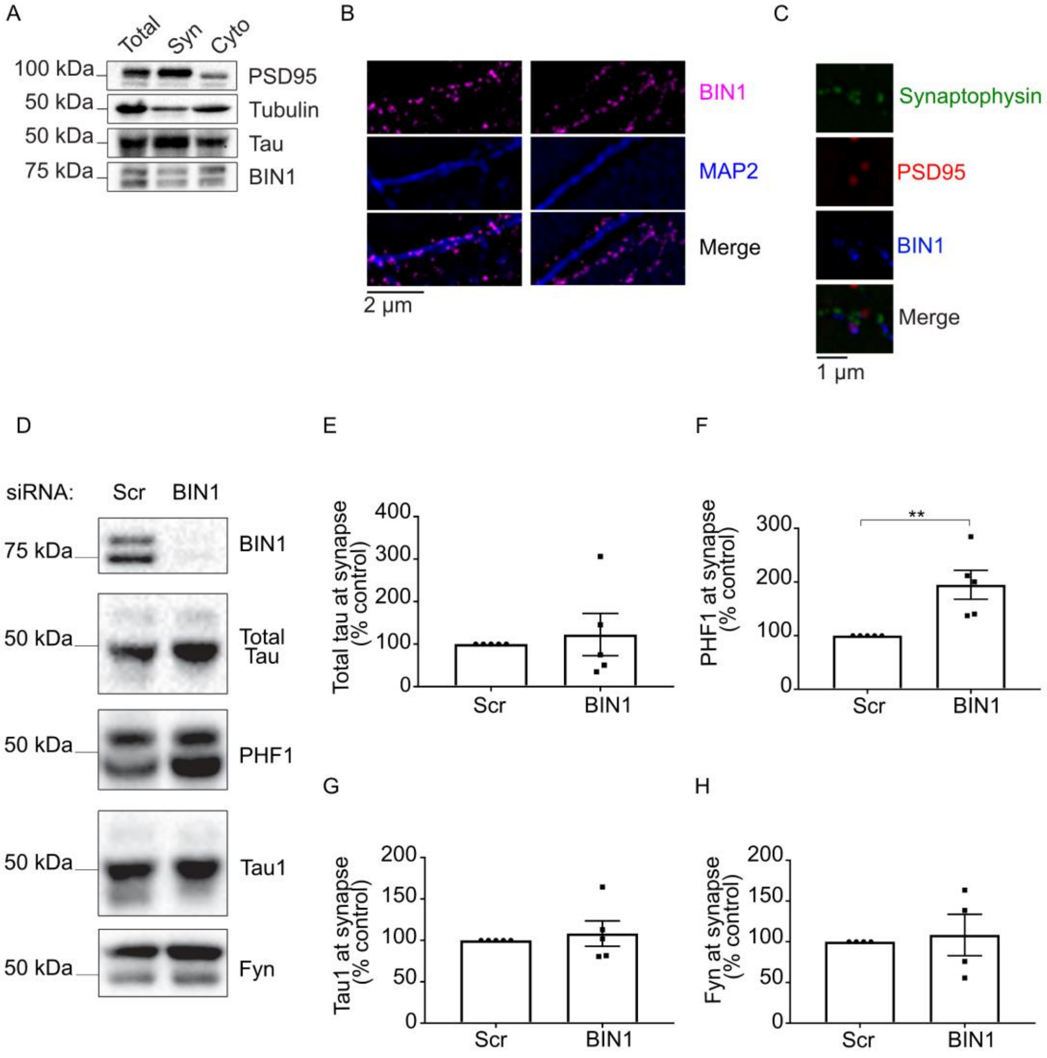

Figure 3: BIN1 knockdown in neurons increases the abundance of phosphorylated tau at synapses. A) Proteins from 22 DIV primary cortical neurons were biochemically fractionated into total, synaptic protein- enriched (syn) and cytoplasmic (cyto) fractions and western blotted with antibodies against PSD95, tubulin, tau and BIN1. Blots show the presence of BIN1 and tau in the synaptic fraction. B) N-SIM super resolution images of primary cortical neurons immunolabelled with antibodies against BIN1 (ab54764, pink) and the dendritic marker MAP2 (blue) showing that BIN1 is present within dendrites and axons in cultured neurons. C) N-SIM super-resolution images show close associations and some colocalization of BIN1 (ab54764, blue) with the pre-synaptic marker synaptophysin (green) and the post-synaptic marker PSD95

(red). D) Lysates from primary cortical neurons transduced with scrambled control shRNA (Scr) lentivirus or BIN1 shRNA (BIN1) lentivirus and biochemically fractionated as above were immunoblotted with antibodies against BIN1, total tau, tau phosphorylated at Ser396/404 (PHF1) and tau dephosphorylated at Ser199/202/Thr205 (Tau-1). Bar charts show quantification of synaptic E) total tau, F) tau phosphorylated at Ser396/404 (PHF1) and G) dephosphorylated tau (Tau-1) protein amounts. Data were normalised to the synaptic marker PSD95 in the same sample and are expressed as percentage mean control (scrambled siRNA). Data are mean ± S.E.M. and were analysed using Mann-Whitney test. n=4-5, **p<0.01.

For Review Only

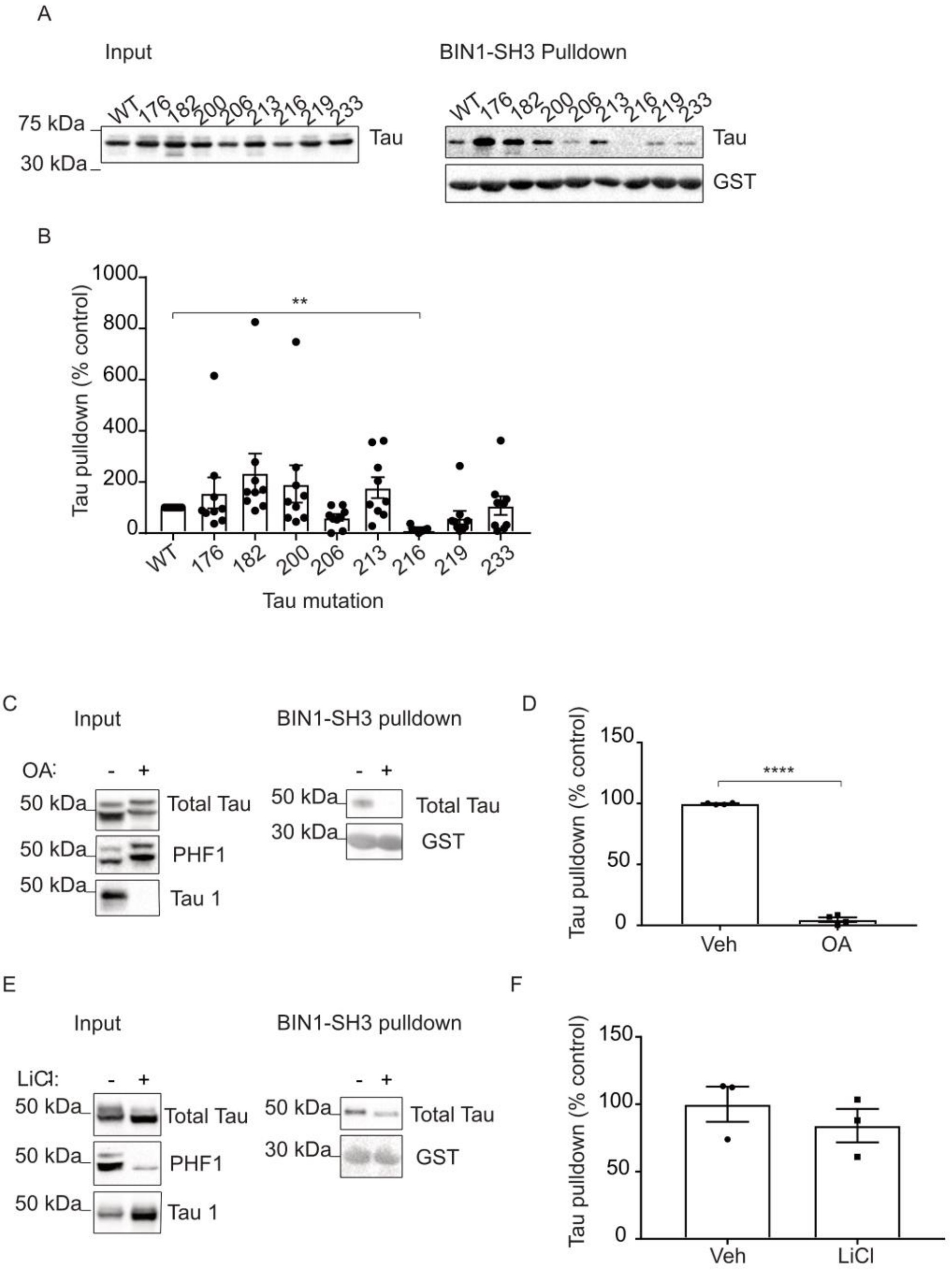

Figure 4: BIN1-SH3 interact via BIN1-SH3 and P216 in tau in a phosphorylation-dependent manner. A) HEK293 cells were transfected with wild type 2N4R tau (WT) or PxxP mutant tau constructs in which a single proline residue at site 176, 182, 200, 206, 213, 216, 219 or 223 was mutated to alanine to disrupt the PxxP sequence. Proteins in lysates from

HEK293 cells (input) were pulled down with BIN1-SH3-GST beads, and western blotted with antibodies against total tau or GST. B) The amount of PXXP mutant tau pulled down by BIN1-SH3-GST was quantified and the bar chart shows this data relative to WT 2N4R tau (control). When P216 was mutated to alanine, tau binding to BIN1-SH3 was significantly reduced (**p=0.001). Following D'Agostino and Pearson normality testing, data were analysed using non-parametric Kruskal-Wallis test and Dunn's multiple comparisons test. Data shown are mean ± S.E.M, n=9. C) Primary cortical neurons were treated with either vehicle (-) or 50 nM okadaic acid (OA,+) for 4 hours. Proteins were pulled down from lysates with BIN1-SH3-GST. Western blots of neuronal lysates (input) with antibodies against total tau, tau phosphorylated at Ser394/404 (PHF1) and tau dephosphorylated at Ser199/202/Thr 205 (Tau-1) show increased tau phosphorylation following okadaic acid treatment. D) Quantification of the amount of tau from vehicle- or okadaic acid-treated neurons pulled down by BIN1-SH3-GST. Data is shown as percentage relative to the mean of controls (vehicle). The amount of tau pulled down by BIN1-SH3-GST was reduced following okadaic acid treatment of primary neurons. Following Shapiro-Wilk normality testing, the data were analysed using an unpaired T-test. Data shown are mean ± S.E.M, n=4. ****p<0.0001. E) Lysates from primary cortical neurons show reduced tau phosphorylation following treatment with 25 mM LiCl (+) for 4 hours relative to vehicle-treated neurons (-). BIN1-SH3-GST pulldowns show that there was no difference in the amount of tau pulled down by BIN1-SH3-GST following LiCl treatment. F) Quantification of the amount of tau from vehicle- or LiCl-treated neurons pulled down by BIN1-SH3-GST. Following Shapiro-Wilk normality testing, data were analysed using a non-parametric Mann-Whitney test. Data shown are mean ± S.E.M, n = 3.

For Review Only

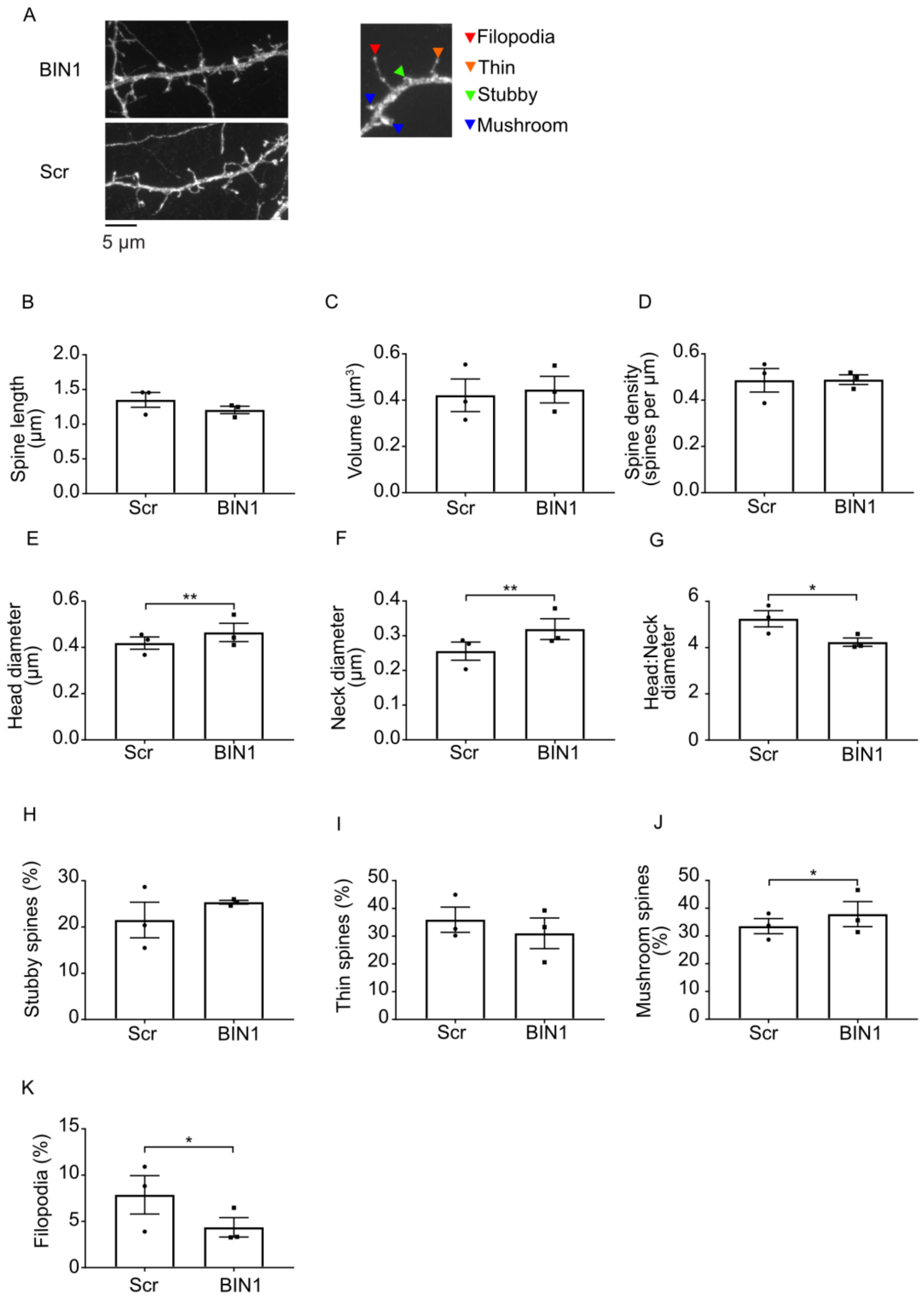

Figure 5: BIN1 knockdown alters dendritic spine morphology. A) Primary cortical neurons transduced with BIN1 shRNA (BIN1) lentivirus (top left) or scrambled control shRNA (Scr) lentivirus (bottom left) were transfected with a plasmid expressing eGFP and fixed at 23 DIV.

Maximum intensity projections were generated from Z-stacks acquired using I-SIM super-resolution imaging. Five to ten different neurons per condition were analysed in each of three separate experiments, one dendrite from each cell was selected randomly for spine quantification and all branches of that dendrite were analysed. Dendritic spines were classified as either filopodia or stubby, thin or mushroom spines (right). Bar charts show quantification of spine B) length, C) volume, D) density, E) head diameter, F) neck diameter, G) ratio of spine head to neck diameter, and percentage of H) stubby, I) thin, J) mushroom spines and K) filopodia. Data are mean ± S.E.M. and were analysed using a randomised block 2-way ANOVA. n=3. *p<0.05, **p<0.01.

For Review Only

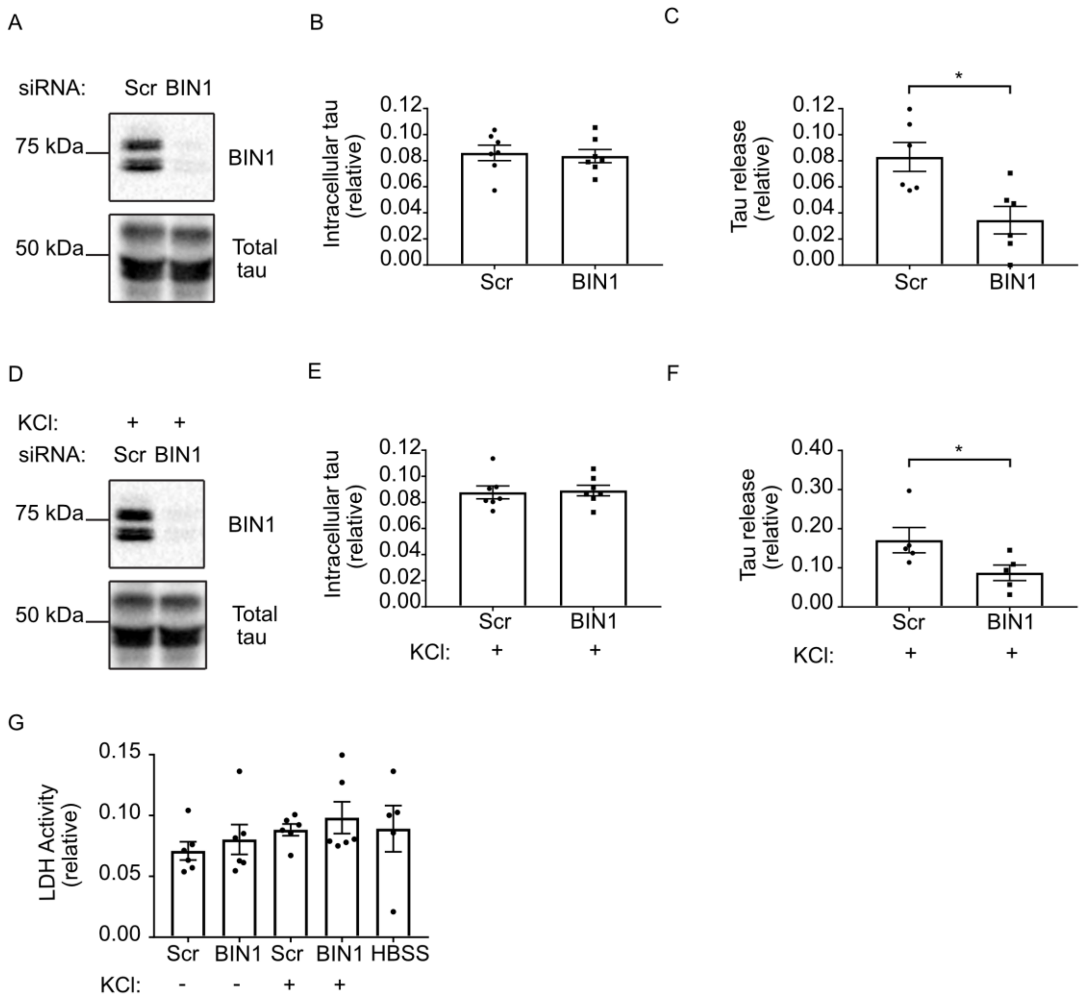

Only

Figure 6: BIN1 knockdown reduces basal and stimulated tau release. A) Cell lysates from 21 DIV primary cortical neurons transduced with scrambled control shRNA (Scr) lentivirus or BIN1 shRNA (BIN1) lentivirus were western blotted with antibodies against BIN1 and total tau. B) Quantification shows no effect of BIN1 knockdown on intracellular tau amount. Shapiro-Wilk normality test demonstrated that the data were normally distributed, and so data were analysed using an unpaired T-test. C) Tau content in conditioned media from neurons was determined by ELISA. Extracellular tau amounts were quantified relative to intracellular tau from the same well, and the data shows reduced tau release upon BIN1 knockdown.

Shapiro-Wilk normality test demonstrated that the data were normally distributed, so data were analysed using an unpaired T-test. D) Cells transduced as above were depolarised with 50 nM KCl (+) for 30 minutes, and the lysates were western blotted with antibodies against BIN1 and total tau. E) KCl treatment had no effect on intracellular tau amounts. Shapiro-Wilk normality test demonstrated that the data were normally distributed, so data were analysed using an unpaired T-test. F) Tau in conditioned media from KCl-stimulated cells was measured as described for basal conditions. Tau release from neurons in which BIN1 was knocked down remained reduced upon neuron depolarisation with KCl. Shapiro-Wilk normality test demonstrated that the data were not normally distributed, so data were analysed using a Mann-Whitney test. G) Lactate dehydrogenase amounts were measured in medium from unstimulated (-) or KCl- stimulated (+) primary cortical neurons transduced with scrambled control shRNA (Scr) lentivirus or BIN1 shRNA (BIN1) lentivirus, and show no effect of treatment on cell viability. Shapiro-Wilk normality test demonstrated that the data were not normally distributed, so data were analysed using a Kruskal-Wallis test with Dunn's multiple comparison test. All graphs show mean ± S.E.M, n=7 (intracellular tau), n= 6 (tau release/intracellular tau, and lactate dehydrogenase assay). *$p<0.05$.

For Review Only

Table 1: Characteristics of temporal cortex tissue used in this study. Table shows details of sex, age, post-mortem delay (hours), Braak stage and Alzheimer's disease diagnosis for cases from which frozen temporal cortex sections was obtained.

**Sex**	**Age** (years)	**Post-mortem delay** (hours)	**Braak stage**	**Diagnosis**
F	74	64	II	Control
F	90	44	II	Control
F	73	27	I	Control
F	77	21	0	Control
F	80	22	II	Control
M	68	60	II	Control
M	80	55	II-III	Control
M	90	45	-	Control
M	78	24	III	Control
F	92	9	II	Control
M	82	47	I	Control
F	84	34	I-II	Control
F	90	50	II	Control
M	66	52	-	Control
M	82	18	I/II	Control

For Review Only

M	91	48	IV	Moderate Alzheimer's disease
M	88	79	III-IV	Moderate Alzheimer's disease
F	95	47	IV	Moderate Alzheimer's disease
M	84	86	IV	Moderate Alzheimer's disease
M	98	53	IV	Moderate Alzheimer's disease
F	86	55.5	IV	Moderate Alzheimer's disease
M	82	28	IV	Moderate Alzheimer's disease
M	86	52.5	IV	Moderate Alzheimer's disease
F	83	22	IV	Moderate Alzheimer's disease

For Review Only

M	93	13.5	IV	Moderate Alzheimer's disease
F	83	41.5	IV	Moderate Alzheimer's disease
F	97	67.5	III-IV	Moderate Alzheimer's disease
F	96	39	IV	Moderate Alzheimer's disease
F	92	19.5	III	Moderate Alzheimer's disease
F	92	29.5	IV	Moderate Alzheimer's disease
F	73	30	VI	Severe Alzheimer's disease
F	84	27	VI	Severe Alzheimer's disease
F	79	31	VI	Severe Alzheimer's disease

For Review Only

M	86	38	VI	Severe Alzheimer's disease
F	85	79	VI	Severe Alzheimer's disease
M	67	39.5	VI	Severe Alzheimer's disease
F	69	73	VI	Severe Alzheimer's disease
F	89	38.5	VI	Severe Alzheimer's disease
F	93	49	VI	Severe Alzheimer's disease
M	84	67	VI	Severe Alzheimer's disease
F	81	20	VI	Severe Alzheimer's disease
M	83	22	VI	Severe Alzheimer's disease

For Review Only

F	81	17.5	VI	Severe Alzheimer's disease
F	86	25	VI	Severe Alzheimer's disease
M	66	41	VI	Severe Alzheimer's disease

For Review Only

Table 2. Summary of temporal cortex cases and controls used in this study. Table shows the percentage of control, moderate Alzheimer's disease and severe Alzheimer's disease cases that were female, the mean age at death (+/-SEM) and the mean post-mortem delay (+/-SEM).

**Disease stage**	**Female** (%)	**Age (years)** Mean +/- SEM	**Post-mortem delay (hours)** Mean +/- SEM
Control	53.3	80.4 ± 2.07	38.1 ± 4.40
Moderate	57.1	89.6 ± 1.42	45.3 ± 5.50
Severe	64.2	80.9 ± 2.10	39.8 ± 5.00

For Review Only

SUPPLEMENTARY DATA

Supplementary Figure 1

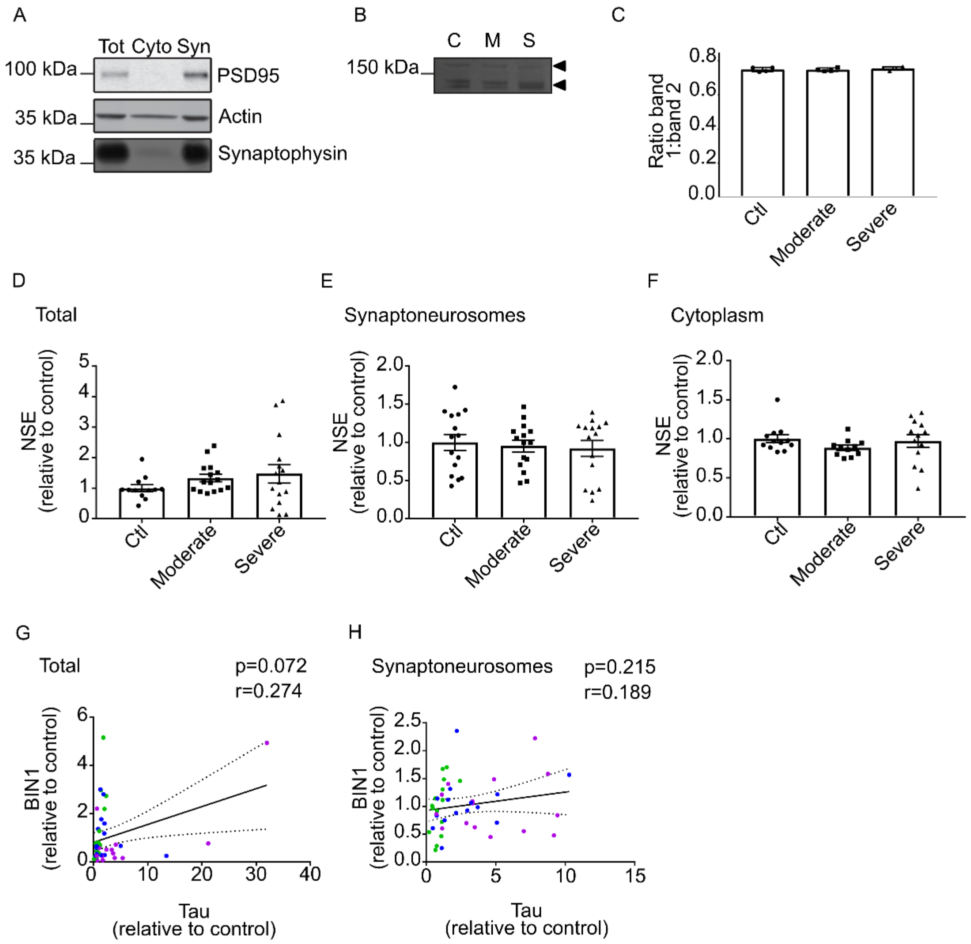

Supplementary Figure 1: Fractionation of Alzheimer's disease temporal cortex to isolate synaptoneurosomes A) Total temporal cortex homogenates and cytoplasmic and synaptoneurosome fractions isolated from the same brain samples were western blotted using antibodies against PSD95 (top), actin (middle), and synaptophysin (bottom) loading to confirm that synaptic proteins are found within the synaptoneurosome but not cytosolic fraction. B) A subset of samples were western blotted using an antibody against the NMDA NR2A subunit which recognises the NR2A subunit (top arrow, 170 kDa) and a degradation product (bottom arrow, 150 kDa) in order to test for sample degradation. The ratio of the top and bottom bands

are quantified in C. Total homogenates, synaptoneurosome and cytoplasmic fractions of control (Braak stage 0-III), moderate (Braak stage III-IV) and severe (Braak stage V-VI) Alzheimer's disease brain were immunoblotted and probed for neuron-specific enolase (NSE) (Figure 3). Bar charts show quantification of NSE in D) total (n=13), E) synaptoneurosome (n=15) and F) cytoplasmic fractions (n=11) following normalisation to controls. Following D'Agostino and Pearson normality testing, data were analysed by Kruskal-Wallis test with Dunn's multiple comparison test (total and cytoplasm) or one-way ANOVA with Tukey's multiple comparisons test (synaptoneurosomes). Graphs show mean ± S.E.M. Correlation analysis of BIN1 and tau amounts in G) total homogenates (n=44), and H) synaptoneurosomes (n=45) shows no correlation between tau and BIN1 in these fractions. Colours in G and H represent mild (green), moderate (blue) and severe (purple) stage samples.

For Review Only

Supplementary Figure 2

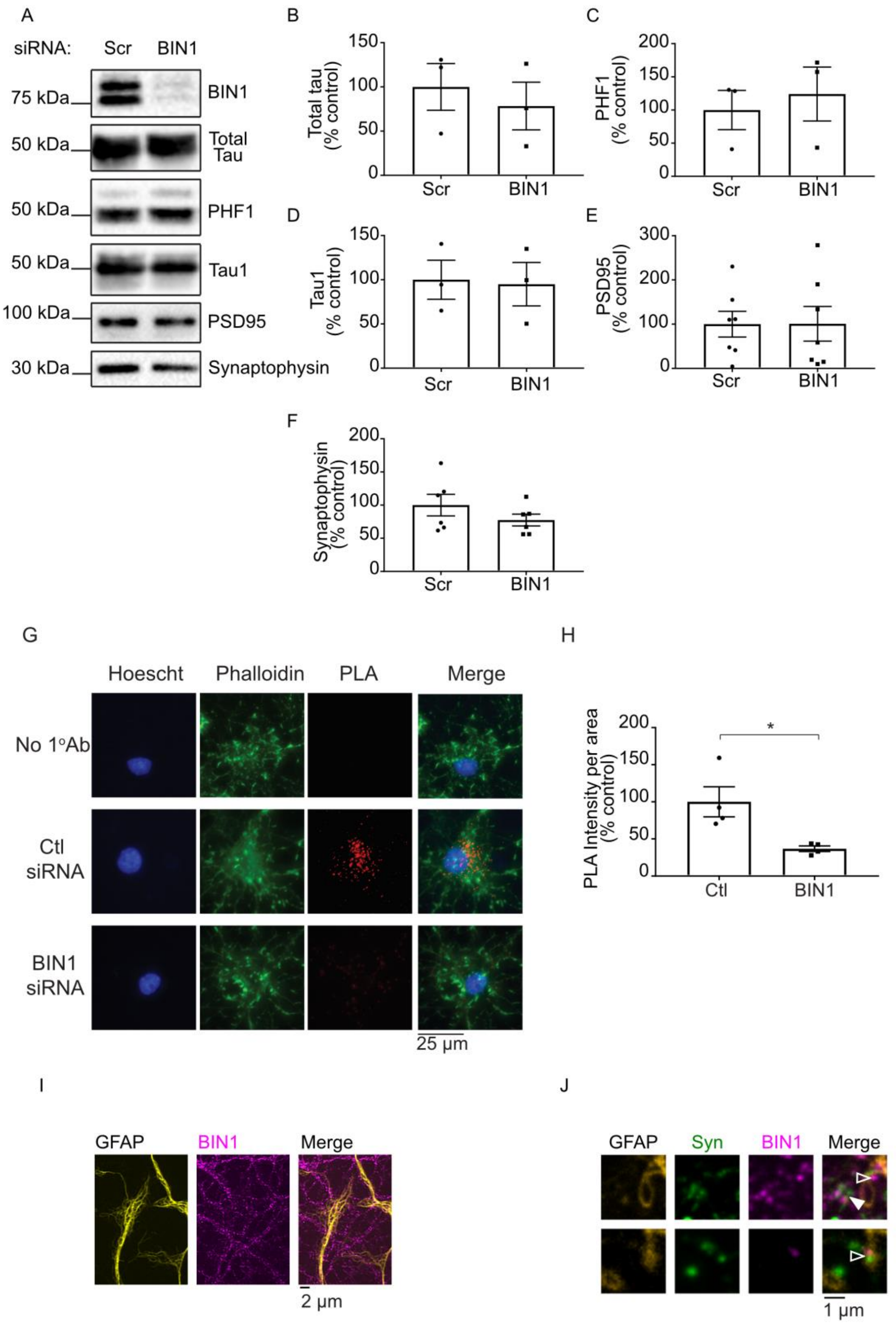

Supplementary Figure 2: BIN1 localization and validation of BIN1 knockdown in cortical neurons. A) Lysates from primary cortical neurons transduced with scrambled control shRNA (Scr) lentivirus or BIN1 shRNA (BIN1) lentivirus were western blotted for BIN1, total tau, tau phosphorylated at Ser396/404 (PHF1), tau dephosphorylated at Ser199/202/Thr205 (tau-1), PSD95, and synaptophysin. Quantification of western blots for B) total tau, C) PHF1, D) tau 1, E) PSD95, and F) synaptophysin. Data are expressed as a percentage of average control (Scr). Following Shapiro-Wilk normality testing total tau, tau1, PSD95, and synaptophysin were analysed using an un-paired T-test. PHF1 data were analysed by Mann-Whitney test. Graphs show the mean ± S.E.M of 3 (total tau, PHF1 tau and tau 1), 7 (PSD95) or 6 (synaptophysin) independent experiments. G) Proximity ligation assays (PLA) were used to demonstrate interactions between endogenous BIN1 and tau in rat primary cortical neurons and to confirm BIN1 knockdown by BIN1 siRNA. Images show PLA signals (red) in neurons treated with Ctl (scrambled) siRNA indicating regions of BIN1-tau interactions. Strong PLA signals identified in the soma of neurons and were markedly reduced upon BIN1 siRNA knockdown, residual interactions likely occurring as a result of incomplete knockdown. Nuclei of cells were stained with Hoechst 33352 (blue) and the actin cytoskeleton labelled with phalloidin (green). While phalloidin can stain glial cells as well as neurons, previous work has shown our cultures contain fewer than 4 % glial cells (Garwood et al., 2011), furthermore tau is not found in glial cells therefore we are confident that the interactions between BIN1 and tau are in neurons. No PLA signals were observed in controls lacking primary antibody or in primary cortical neurons treated with BIN1 siRNA. Bar chart shows significantly reduced PLA signal intensity/area in neurons exposed to BIN1 siRNA (BIN1) relative to non-targeting control (Ctl) siRNA. Data were analysed using Mann-Whitney tests. Data shown are mean ±S.E.M. n=3, *$p<0.05$. H) Instant SIM images show a close association of some BIN1 puncta (ab54764, pink) with astrocytes (GFAP, yellow). I) Some BIN1 (ab54764, pink) close to

For Review Only

synaptophysin (syp, green) puncta were found in association with the distal end of astrocyte processes (GFAP, yellow, open arrow heads), however many BIN1-synaptophysin pairs did not (closed arrow heads).

For Review Only

Supplementary Figure 3

A

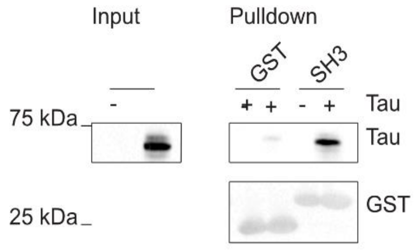

B

151 IATPRGAAPP GQKGQANATR IPAKT**p**PAPK T**p**PSSGEPPK SGDRSGYSS**p** 200
201 GSPGT**p**GSRS RT**p**SL**p**TP**p**T REPKKVAVVR TP**p**KSPSSAK SRLQTAPVPM 250

For Review Only

Supplementary Figure 3: BIN1-SH3 binds to PXXP motifs in tau. A) Lysates from HEK293 cells transfected with empty vector (-) or 2N4R human tau (+) were incubated with BIN1-SH3-GST glutathione beads, or GST- beads as a control. Lysates (input) and GST-bound proteins (pulldown) were probed on western blots with antibodies against total (phosphorylated and nonphosphorylated) tau (top) or GST (bottom). Tau was pulled down by BIN1-SH3-GST but not GST-only, no interactions were observed with GST-only and empty vector controls. Specificity of the signal was confirmed by siRNA-mediated BIN1 knockdown. B) Amino acid sequence 151-250 of human 2N4R tau in which the seven PxxP motifs are underlined, and prolines in these motifs that were mutated to alanine are indicated in lowercase and bold type.

Supplementary Figure 4

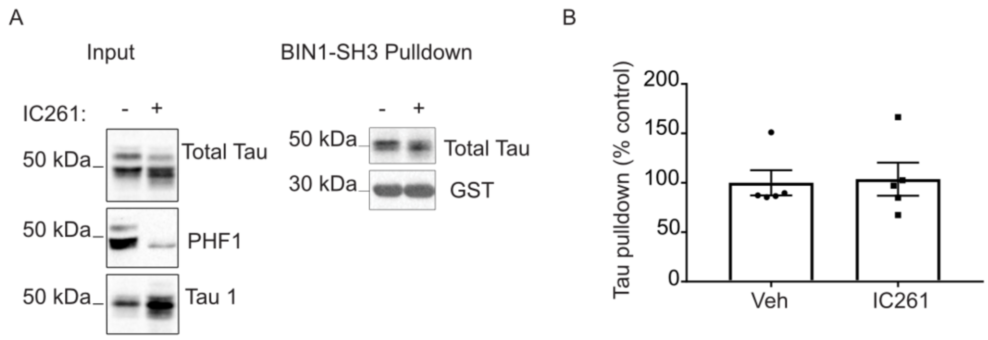

Supplementary Figure 4: Inhibiting casein-kinase 1 activity to reduce tau phosphorylation does not alter the interaction of tau with BIN1-SH3. A) Lysates from 21-23 DIV primary cortical neurons show reduced tau phosphorylation following treatment with 20 µM IC261 (+) for 4 hours relative to vehicle-treated neurons (-). BIN1-SH3-GST pulldowns show that there was no apparent difference in the amount of tau pulled down by BIN1-SH3-GST following reduction of tau phosphorylation by IC261 treatment. B) Quantification of the amount of tau from vehicle- or IC261-treated neurons pulled down by BIN1-SH3-GST shown as percentage mean control (vehicle). Following Shapiro-Wilk normality testing, data were analysed using a Mann-Whitney test. Data is mean ± S.E.M., n = 3.

For Review Only

Supplementary Figure 5

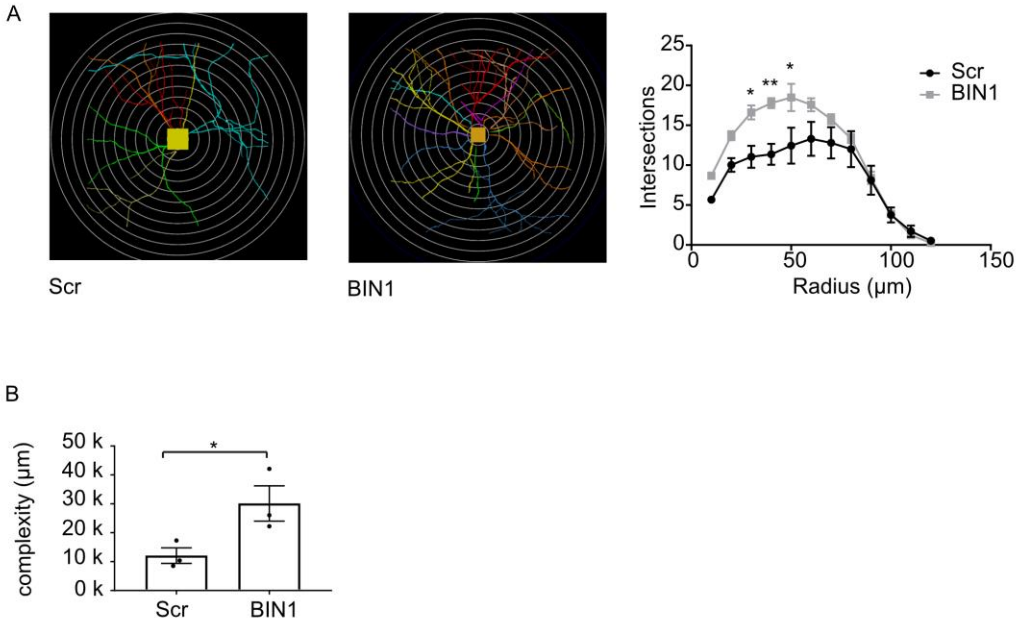

Supplementary Figure 5: BIN1 Knockdown alters neuronal complexity. A) Primary cortical neurons transduced with BIN1 shRNA (BIN1) lentivirus (top left) or scrambled control shRNA (Scr) lentivirus (bottom left) were transfected with a plasmid expressing eGFP and fixed at 23 DIV. Maximum intensity projections were generated from Z-stacks acquired using I-SIM super-resolution imaging. Five to ten different neurons per condition were analysed in each of three separate experiments. The number of intersections at each radius were quantified (graph). Shapiro-Wilk test showed the data were normally distributed, data were analysed by One-way ANOVA with Sidak's multiple comparisons test. B) Quantification of complexity of primary cortical neurons transduced with scrambled control shRNA (Scr) lentivirus or BIN1 shRNA (BIN1) lentivirus. Complexity was calculated as (Sum of the terminal orders + Number of terminals) * (Total dendritic length / Number of primary dendrites]), where terminals is the number of branch endings, and terminal orders is the number of branches between the terminal

and the cell body. Data are mean ± S.E.M. and were analysed using a randomised block 2-way ANOVA. n=3. Graphs show mean ±S.E.M of 3 independent experiments. *p<0.05, **p<0.01.

For Review Only

Supplementary methods

Proximity Ligation Assays

Proximity ligation assays were performed as described by us previously (Gomez-Suaga et al., 2019) in 23DIV neurons using primary antibodies against BIN1 (ab54764, Abcam, Cambridge, UK) and total tau (Agilent, CA, USA). The actin cytoskeleton was labelled with phalloidin-488 (Life Technologies, CA, USA), and nuclei were stained with 10 μgml^{-1} Hoescht-33352 (Thermo Fischer Scientific). Image stacks covering the whole volume of each cell were acquired using a Nikon Eclipse Ti-E microscope and images analysed using Fiji. Z-stacks were converted to maximum intensity projections, and the phalloidin signal used to identify cell outlines. The intensity of duo-link signals in each cell was quantified using image J and is expressed as a proportion of cell area.

For Review Only

Analysis of neuronal complexity

For analysis of dendrite structure, neurons at 22 DIV were transfected with an eGFP-N2 plasmid (Clontech, Kyoto, Japan) using lipofectamine 2000 for 24 hours, fixed and the GFP signal imaged using a Nikon Eclipse Ti-2 inverted microscope with Vt-iSIM scan head. 3x3 large image stacks were acquired covering the entire volume of the neuron, with 0.2 µm between each image in the Z plane. Neurolucida™ software (MBF Bioscience, VT, USA) was used to trace neurons and detect, classify and quantify the dendritic spines, and perform Scholl analysis. Neuronal complexity was determined as (sum of terminal orders + number of terminals) * (total dendritic length / number of primary dendrites]), where terminals is the number of branch endings, and terminal order is the number of branches between the terminal and the cell body (Pillai et al., 2012).

References

Garwood CJ, Pooler AM, Atherton J, Hanger DP, Noble W. Astrocytes are important mediators of Abeta-induced neurotoxicity and tau phosphorylation in primary culture. 2011. Cell Death Disease. 2:e167.

Gomez-Suaga P, Perez-Nievas BG, Glennon EB, Lau DHW, Paillusson S, Morotz GM, et al. The VAPB-PTPIP51 endoplasmic reticulum-mitochondria tethering proteins are present in neuronal synapses and regulate synaptic activity. 2019. Acta Neuropathol Commun. 7:35.

Pillai AG, de Jong D, Kanatsou S, Krugers H, Knapman A, Heinzmann JM, et al. Dendritic morphology of hippocampal and amygdalar neurons in adolescent mice is resilient to genetic differences in stress reactivity. 2102. PLoS One. 7:e38971.

For Review Only

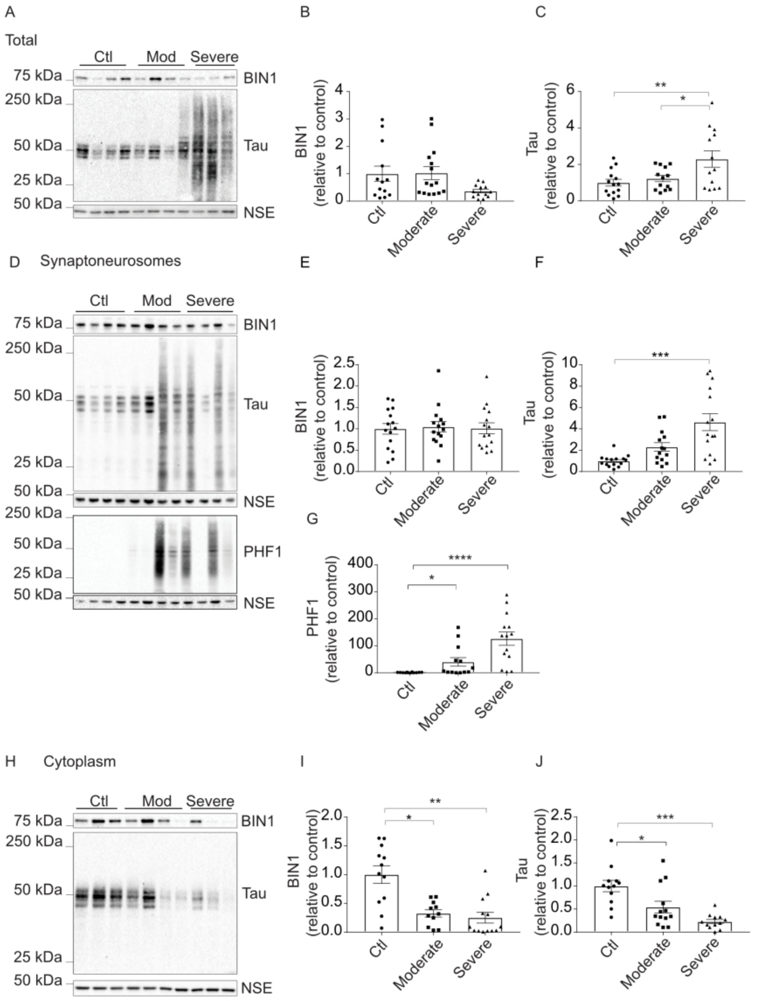

Figure 1. BIN1 and tau are lost from the cytoplasm and this is associated with accumulation of synaptic phosphorylated tau in Alzheimer's disease temporal cortex. A) Total homogenates from temporal cortex of control (Braak stage 0-III), moderate (Braak stage III-IV) and severe (Braak stage V-VI) Alzheimer's disease brain were western blotted using antibodies against BIN1, total tau, and neuron specific enolase (NSE). Bar charts show quantification of B) BIN1 and C) tau amounts following normalisation to NSE in the same sample. Data shown are mean ± S.E.M. expressed as fold average control. Following D'Agostino and Pearson normality testing, data were analysed using a one-way ANOVA with Holm-Sidak's multiple comparisons test. n=13 per group (BIN1) or 14 per group (tau). D) Synaptoneurosomes isolated from the same temporal cortex samples were also immunoblotted with antibodies against BIN1, tau, tau phosphorylated at Ser396/404 (PHF1), and neuron-specific enolase (NSE). Bar charts show quantification of E) BIN1, F) tau, and G) PHF1 in synaptoneurosome fractions following normalisation to NSE in the same sample. Data are mean ± S.E.M. expressed as fold average control. Following D'Agostino and Pearson normality testing, data was analysed using non-parametric Kruskal-Wallis test with Dunn's multiple comparison test. n=15 per group (BIN1 and tau) or 12 per group (PHF1). H) The cytoplasmic fraction was

blotted as above with antibodies against BIN1, tau, and NSE. Bar charts show quantification of I) BIN1 and J) tau in the cytoplasmic fraction following normalisation to NSE in the same sample. Data are mean ± S.E.M. expressed as fold mean control. Following D'Agostino and Pearson normality testing, BIN1 data was analysed using non-parametric Kruskal-Wallis test with Dunn's multiple comparison test and tau data using a one-way ANOVA with Holm-Sidak's multiple comparisons test. n=11 per group (BIN1) or 12 per group (tau). *$p<0.05$, ** $p<0.01$, ***$p<0.001$, ****$p<0.0001$.

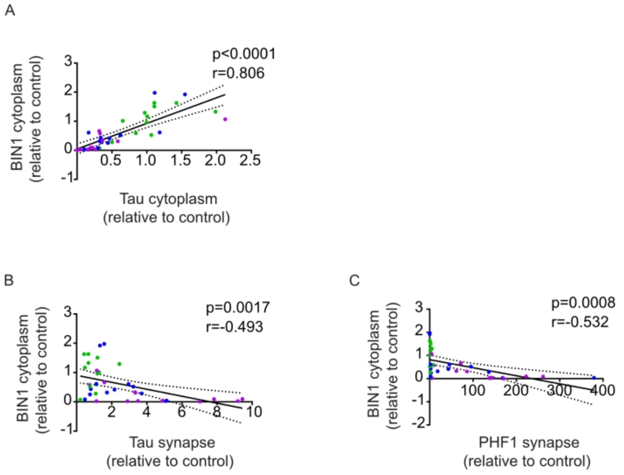

Figure 2. Loss of BIN1 correlates with loss of cytoplasmic tau and increased synaptic tau in Alzheimer's disease temporal cortex. Correlation analysis of BIN1 and tau amounts in A) cytoplasmic fractions shows a strong positive correlation between BIN1 and tau (n=38), and strong negative correlations between B) cytoplasmic BIN1 and synaptic tau (n=38), and C) cytoplasmic BIN1 and synaptic tau phosphorylated at Ser396/404 (PHF1) (n=36). Colours represent control (green), moderate (blue) and severe (purple) Alzheimer's disease Braak stage samples.

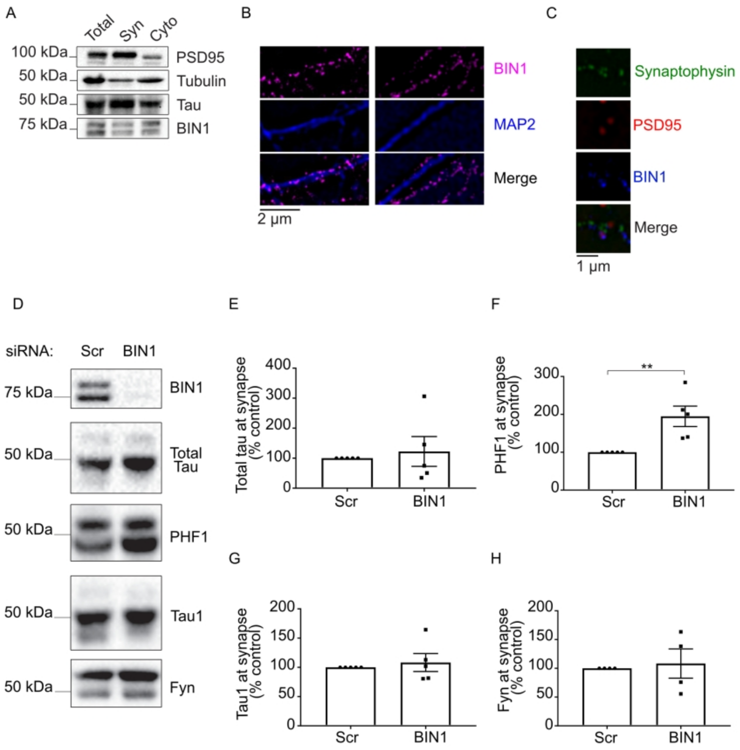

Figure 3: BIN1 knockdown in neurons increases the abundance of phosphorylated tau at synapses. A) Proteins from 22 DIV primary cortical neurons were biochemically fractionated into total, synaptic protein-enriched (syn) and cytoplasmic (cyto) fractions and western blotted with antibodies against PSD95, tubulin, tau and BIN1. Blots show the presence of BIN1 and tau in the synaptic fraction. B) N-SIM super resolution images of primary cortical neurons immunolabelled with antibodies against BIN1 (ab54764, pink) and the dendritic marker MAP2 (blue) showing that BIN1 is present within dendrites and axons in cultured neurons. C) N-SIM super-resolution images show close associations and some colocalization of BIN1 (ab54764, blue) with the pre-synaptic marker synaptophysin (green) and the post-synaptic marker PSD95 (red). D) Lysates from primary cortical neurons transduced with scrambled control shRNA (Scr) lentivirus or BIN1 shRNA (BIN1) lentivirus and biochemically fractionated as above were immunoblotted with antibodies against BIN1, total tau, tau phosphorylated at Ser396/404 (PHF1) and tau dephosphorylated at Ser199/202/Thr205 (Tau-1). Bar charts show quantification of synaptic E) total tau, F) tau phosphorylated at Ser396/404 (PHF1) and G) dephosphorylated tau (Tau-1) protein amounts. Data were normalised to the synaptic marker PSD95 in the same sample and are expressed as percentage mean control (scrambled siRNA). Data are mean ±

S.E.M. and were analysed using Mann-Whitney test. n=4-5, **$p<0.01$.

209x296mm (300 x 300 DPI)

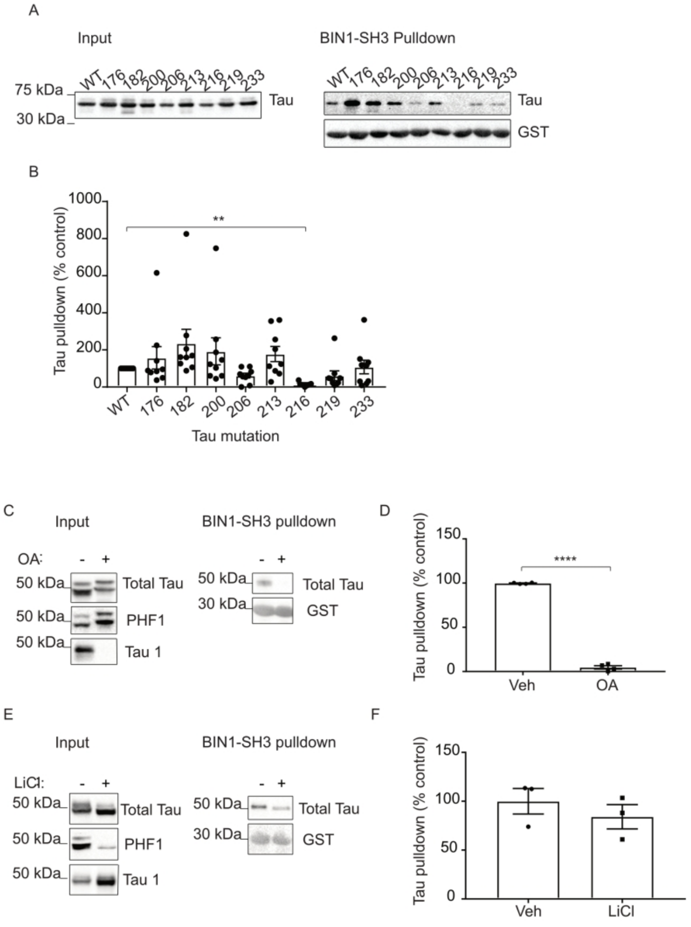

Figure 4: BIN1-SH3 interact via BIN1-SH3 and P216 in tau in a phosphorylation-dependent manner. A) HEK293 cells were transfected with wild type 2N4R tau (WT) or PxxP mutant tau constructs in which a single proline residue at site 176, 182, 200, 206, 213, 216, 219 or 223 was mutated to alanine to disrupt the PxxP sequence. Proteins in lysates from HEK293 cells (input) were pulled down with BIN1-SH3-GST beads, and western blotted with antibodies against total tau or GST. B) The amount of PXXP mutant tau pulled down by BIN1-SH3-GST was quantified and the bar chart shows this data relative to WT 2N4R tau (control). When P216 was mutated to alanine, tau binding to BIN1-SH3 was significantly reduced (**p=0.001). Following D'Agostino and Pearson normality testing, data were analysed using non-parametric Kruskal-Wallis test and Dunn's multiple comparisons test. Data shown are mean ± S.E.M, n=9. C) Primary cortical neurons were treated with either vehicle (-) or 50 nM okadaic acid (OA,+) for 4 hours. Proteins were pulled down from lysates with BIN1-SH3-GST. Western blots of neuronal lysates (input) with antibodies against total tau, tau phosphorylated at Ser394/404 (PHF1) and tau dephosphorylated at Ser199/202/Thr 205 (Tau-1) show increased tau phosphorylation following okadaic acid treatment. D) Quantification of the amount of tau from vehicle- or okadaic acid-treated neurons pulled down by BIN1-SH3-GST. Data is shown as percentage

relative to the mean of controls (vehicle). The amount of tau pulled down by BIN1-SH3-GST was reduced following okadaic acid treatment of primary neurons. Following Shapiro-Wilk normality testing, the data were analysed using an unpaired T-test. Data shown are mean ± S.E.M, n=4. ****$p<0.0001$. E) Lysates from primary cortical neurons show reduced tau phosphorylation following treatment with 25 mM LiCl (+) for 4 hours relative to vehicle-treated neurons (-). BIN1-SH3-GST pulldowns show that there was no difference in the amount of tau pulled down by BIN1-SH3-GST following LiCl treatment. F) Quantification of the amount of tau from vehicle- or LiCl-treated neurons pulled down by BIN1-SH3-GST. Following Shapiro-Wilk normality testing, data were analysed using a non-parametric Mann-Whitney test. Data shown are mean ± S.E.M, n = 3.

209x296mm (300 x 300 DPI)

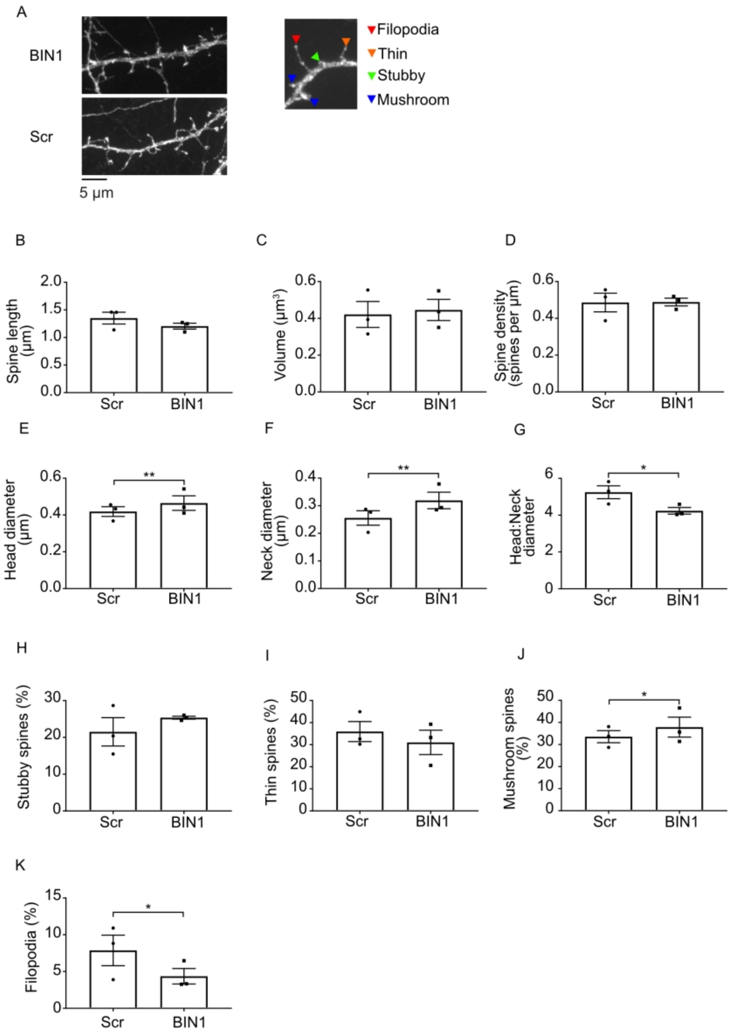

Figure 5: BIN1 knockdown alters dendritic spine morphology. A) Primary cortical neurons transduced with BIN1 shRNA (BIN1) lentivirus (top left) or scrambled control shRNA (Scr) lentivirus (bottom left) were transfected with a plasmid expressing eGFP and fixed at 23 DIV. Maximum intensity projections were generated from Z-stacks acquired using I-SIM super-resolution imaging. Five to ten different neurons per condition were analysed in each of three separate experiments, one dendrite from each cell was selected randomly for spine quantification and all branches of that dendrite were analysed. Dendritic spines were classified as either filopodia or stubby, thin or mushroom spines (right). Bar charts show quantification of spine B) length, C) volume, D) density, E) head diameter, F) neck diameter, G) ratio of spine head to neck diameter, and percentage of H) stubby, I) thin, J) mushroom spines and K) filopodia. Data are mean ± S.E.M. and were analysed using a randomised block 2-way ANOVA. n=3. *p<0.05, **p<0.01.

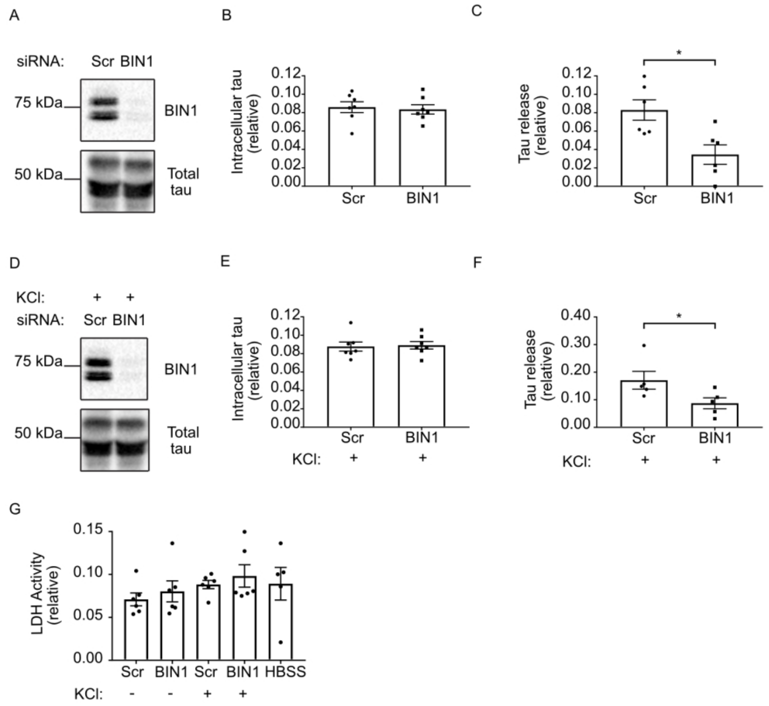

Figure 6: BIN1 knockdown reduces basal and stimulated tau release. A) Cell lysates from 21 DIV primary cortical neurons transduced with scrambled control shRNA (Scr) lentivirus or BIN1 shRNA (BIN1) lentivirus were western blotted with antibodies against BIN1 and total tau. B) Quantification shows no effect of BIN1 knockdown on intracellular tau amount. Shapiro-Wilk normality test demonstrated that the data were normally distributed, and so data were analysed using an unpaired T-test. C) Tau content in conditioned media from neurons was determined by ELISA. Extracellular tau amounts were quantified relative to intracellular tau from the same well, and the data shows reduced tau release upon BIN1 knockdown. Shapiro-Wilk normality test demonstrated that the data were normally distributed, so data were analysed using an unpaired T-test. D) Cells transduced as above were depolarised with 50 nM KCl (+) for 30 minutes, and the lysates were western blotted with antibodies against BIN1 and total tau. E) KCl treatment had no effect on intracellular tau amounts. Shapiro-Wilk normality test demonstrated that the data were normally distributed, so data were analysed using an unpaired T-test. F) Tau in conditioned media from KCl-stimulated cells was measured as described for basal conditions. Tau release from neurons in which BIN1 was knocked down remained reduced upon neuron depolarisation with KCl. Shapiro-Wilk normality test

demonstrated that the data were not normally distributed, so data were analysed using a Mann-Whitney test. G) Lactate dehydrogenase amounts were measured in medium from unstimulated (-) or KCl-stimulated (+) primary cortical neurons transduced with scrambled control shRNA (Scr) lentivirus or BIN1 shRNA (BIN1) lentivirus, and show no effect of treatment on cell viability. Shapiro-Wilk normality test demonstrated that the data were not normally distributed, so data were analysed using a Kruskal-Wallis test with Dunn's multiple comparison test. All graphs show mean ± S.E.M, n=7 (intracellular tau), n= 6 (tau release/intracellular tau, and lactate dehydrogenase assay). *p<0.05.

Table 1: Characteristics of temporal cortex tissue used in this study. Table shows details of sex, age, post-mortem delay (hours), Braak stage and Alzheimer's disease diagnosis for cases from which frozen temporal cortex sections was obtained.

**Sex**	**Age** (years)	**Post-mortem delay** (hours)	**Braak stage**	**Diagnosis**
F	74	64	II	Control
F	90	44	II	Control
F	73	27	I	Control
F	77	21	0	Control
F	80	22	II	Control
M	68	60	II	Control
M	80	55	II-III	Control
M	90	45	-	Control
M	78	24	III	Control
F	92	9	II	Control
M	82	47	I	Control
F	84	34	I-II	Control
F	90	50	II	Control
M	66	52	-	Control
M	82	18	I/II	Control

For Review Only

M	91	48	IV	Moderate Alzheimer's disease
M	88	79	III-IV	Moderate Alzheimer's disease
F	95	47	IV	Moderate Alzheimer's disease
M	84	86	IV	Moderate Alzheimer's disease
M	98	53	IV	Moderate Alzheimer's disease
F	86	55.5	IV	Moderate Alzheimer's disease
M	82	28	IV	Moderate Alzheimer's disease
M	86	52.5	IV	Moderate Alzheimer's disease
F	83	22	IV	Moderate Alzheimer's disease

For Review Only

M	93	13.5	IV	Moderate Alzheimer's disease
F	83	41.5	IV	Moderate Alzheimer's disease
F	97	67.5	III-IV	Moderate Alzheimer's disease
F	96	39	IV	Moderate Alzheimer's disease
F	92	19.5	III	Moderate Alzheimer's disease
F	92	29.5	IV	Moderate Alzheimer's disease
F	73	30	VI	Severe Alzheimer's disease
F	84	27	VI	Severe Alzheimer's disease
F	79	31	VI	Severe Alzheimer's disease

For Review Only

M	86	38	VI	Severe Alzheimer's disease
F	85	79	VI	Severe Alzheimer's disease
M	67	39.5	VI	Severe Alzheimer's disease
F	69	73	VI	Severe Alzheimer's disease
F	89	38.5	VI	Severe Alzheimer's disease
F	93	49	VI	Severe Alzheimer's disease
M	84	67	VI	Severe Alzheimer's disease
F	81	20	VI	Severe Alzheimer's disease
M	83	22	VI	Severe Alzheimer's disease

For Review Only

F	81	17.5	VI	Severe Alzheimer's disease
F	86	25	VI	Severe Alzheimer's disease
M	66	41	VI	Severe Alzheimer's disease

For Review Only

Table 2. Summary of temporal cortex cases and controls used in this study. Table shows the percentage of control, moderate Alzheimer's disease and severe Alzheimer's disease cases that were female, the mean age at death (+/-SEM) and the mean post-mortem delay (+/-SEM).

**Disease stage**	**Female** (%)	**Age (years)**<br>Mean +/- SEM	**Post-mortem delay (hours)**<br>Mean +/- SEM
Control	53.3	80.4 ± 2.07	38.1 ± 4.40
Moderate	57.1	89.6 ± 1.42	45.3 ± 5.50
Severe	64.2	80.9 ± 2.10	39.8 ± 5.00

For Review Only
