# Supplementary material for: Bridging integrator 1 protein loss in Alzheimer’s disease promotes synaptic tau accumulation and disrupts tau release
Source: Brain Commun. 2020 Feb 14;2(1):fcaa011. doi: 10.1093/braincomms/fcaa011 (PMC7272218; doi:10.1093/braincomms/fcaa011)
Supplement: fcaa011_Supplementary_Data [file fcaa011_supplementary_data.zip › Supplementary_figures_and_methods.pdf]

SUPPLEMENTARY DATA

Supplementary Figure 1

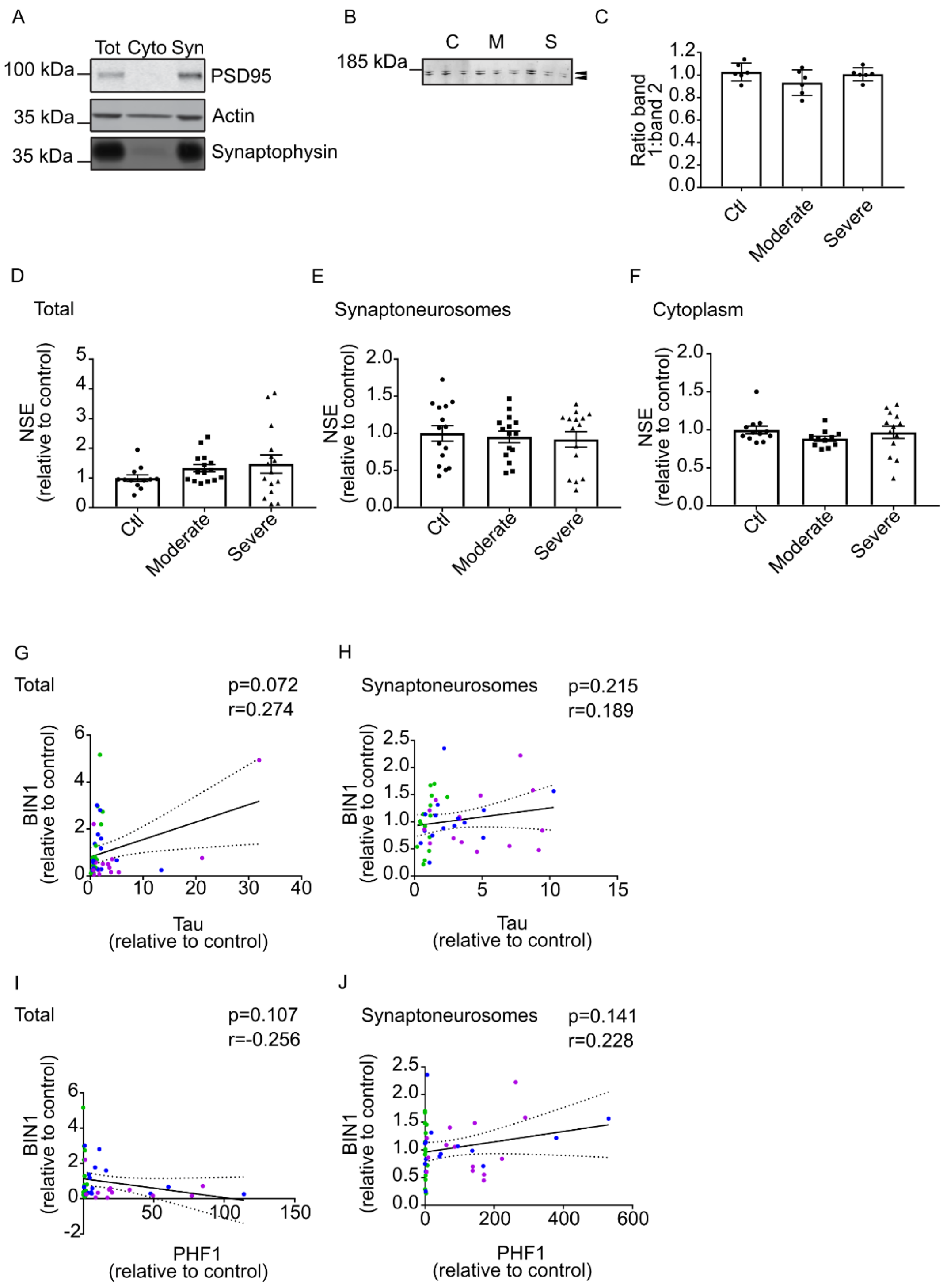

Supplementary Figure 1: Fractionation of Alzheimer's disease temporal cortex to isolate synaptoneurosomes A) Equal protein concentrations of total temporal cortex homogenates

together with cytoplasmic and synaptoneurosome fractions isolated from the same brain samples were western blotted using antibodies against PSD95, actin, and synaptophysin to confirm that synaptic proteins are found within the synaptoneurosome but not cytosolic fraction. B) To test for synaptic integrity, a subset of samples were western blotted using an antibody against the NMDA NR2B subunit which recognises full-length NR2B (top arrow head) and degradation products (bottom arrowhead). The ratio of the top/bottom bands are quantified in C and show reasonable preservation of synaptic integrity in these samples, with no differences between groups. Total homogenates, synaptoneurosome and cytoplasmic fractions of control (Braak stage 0-III), moderate (Braak stage III-IV) and severe (Braak stage V-VI) Alzheimer's disease brain were immunoblotted and probed for neuron-specific enolase (NSE). Bar charts show quantification of NSE in D) total (n=13), E) synaptoneurosome (n=15) and F) cytoplasmic fractions (n=11) following normalisation to controls. There are no significant alterations in this neuronal marker between groups. Following D'Agostino and Pearson normality testing, data were analysed by Kruskal-Wallis test with Dunn's multiple comparison test (total and cytoplasm) or one-way ANOVA with Tukey's multiple comparisons test (synaptoneurosomes). Graphs show mean ± S.E.M. Correlation analysis of BIN1 and tau amounts in G) total homogenates (n=44), and H) synaptoneurosomes (n=45), and BIN1, and I) phosphorylated tau (pSer396/404, PHF1) in total homogenates (n=42) and J) synaptoneurosomes (n=45) shows no correlation between total tau or phosphorylated tau and BIN1 in these fractions. Colours in G, H, I and J represent mild (green), moderate (blue) and severe (purple) stage samples. Full uncut western blots can be found in the supplementary material.

Supplementary Figure 2

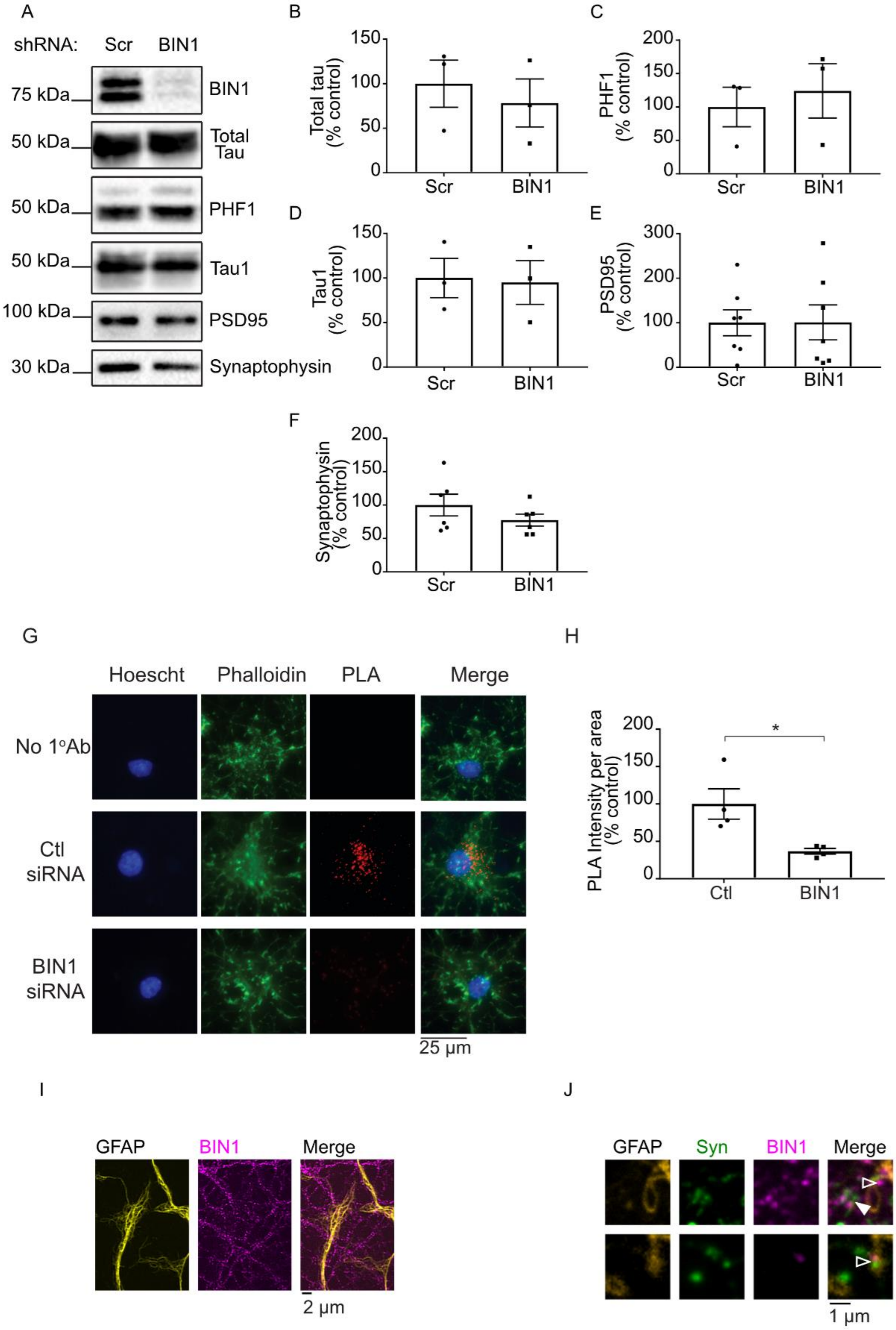

Supplementary Figure 2: BIN1 localization and validation of BIN1 knockdown in cortical neurons. A) Lysates of equivalent protein concentration (as determined by Ponceau red staining of membranes) from primary cortical neurons transduced with scrambled control shRNA (Scr) lentivirus or BIN1 shRNA (BIN1) lentivirus at 5 DIV were western blotted for BIN1, total tau, tau phosphorylated at Ser396/404 (PHF1), tau dephosphorylated at Ser199/202/Thr205 (tau-1), PSD95, and synaptophysin. Quantification of western blots for B) total tau, C) PHF1, D) tau 1, E) PSD95, and F) synaptophysin. Data are expressed as a percentage of average control (Scr). Following Shapiro-Wilk normality testing total tau, tau1, PSD95, and synaptophysin were analysed using an un-paired T-test. PHF1 data were analysed by Mann-Whitney test. Graphs show the mean ± S.E.M of 3 (total tau, PHF1 tau and tau 1), 7 (PSD95) or 6 (synaptophysin) independent experiments. G) Proximity ligation assays (PLA) were used to demonstrate interactions between endogenous BIN1 and tau in rat primary cortical neurons and to confirm BIN1 knockdown by BIN1 siRNA. Images show PLA signals (red) in neurons treated with Ctl (scrambled) siRNA indicating regions of BIN1-tau interactions. Strong PLA signals identified in the soma of neurons were markedly reduced upon BIN1 siRNA knockdown, residual interactions likely occurring as a result of incomplete knockdown. Nuclei of cells were stained with Hoechst 33352 (blue) and the actin cytoskeleton was labelled with phalloidin (green). While phalloidin can stain glial cells as well as neurons, previous work has shown our cultures contain fewer than 4 % glial cells (Garwood *et al*., 2011), furthermore tau is not found in rodent glial cells and therefore we are confident that the interactions between BIN1 and tau are in neurons. Only trace levels of PLA signals were observed in controls lacking primary antibody or in primary cortical neurons treated with BIN1 siRNA. H) Bar chart shows significantly reduced PLA signal intensity/area in neurons exposed to BIN1 siRNA (BIN1) relative to non-targeting control (Ctl) siRNA at 19 DIV. Data were analysed using Mann-Whitney tests. Data shown are mean ±S.E.M. n=3, *$p<0.05$. I) Instant SIM images show a

close association of some BIN1 puncta (ab54764, pink) with astrocytes (GFAP, yellow). J) Some BIN1 (ab54764, pink) close to synaptophysin (syp, green) puncta were found in association with the distal end of astrocyte processes (GFAP, yellow, open arrow heads), however many BIN1-synaptophysin pairs were not (closed arrow heads). Full uncut western blots can be found in the supplementary material.

Supplementary Figure 3

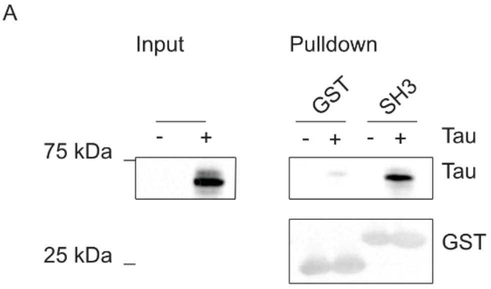

B

```
151  IATPRGAAPP GQKGQANATR IPAKTpPAPK TpPSSGEPPK SGDRSGYSSp  200
201  GSPGTpGSRS RTpSLpTPpT REPKKVAVVR TPpKSPSSAK SRLQTAPVPM  250
```

Supplementary Figure 3: BIN1-SH3 binds to PXXP motifs in tau. A) Lysates from HEK293 cells transfected with empty vector (-) or 2N4R human tau (+) were incubated with BIN1-SH3-GST (SH3) glutathione beads, or GST- (GST) beads as a control. Lysates (input) and GST-bound proteins (pulldown) were probed on western blots with antibodies against total (phosphorylated and nonphosphorylated) tau or GST. Tau was pulled down by BIN1-SH3-GST but not GST- only, no interactions were observed with GST-only and empty vector controls. B) Amino acid sequence 151-250 of human 2N4R tau in which the seven PxxP motifs are underlined, and prolines in these motifs that were mutated to alanine are indicated in lowercase and bold type. Full uncut western blots can be found in the supplementary material.

Supplementary Figure 4

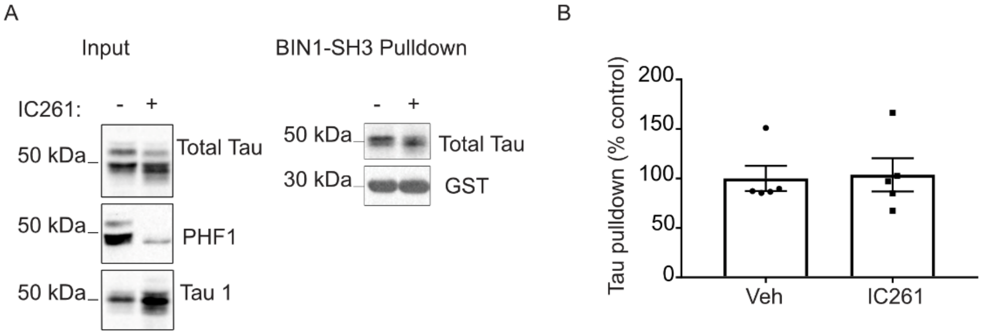

Supplementary Figure 4: Inhibiting casein-kinase 1 activity to reduce tau phosphorylation does not alter the interaction of tau with BIN1-SH3. A) Lysates from 21-23 DIV primary cortical neurons show reduced tau phosphorylation following treatment with 20 μM IC261 (+) for 4 hours relative to vehicle-treated neurons (-). BIN1-SH3-GST pulldowns show that there was no apparent difference in the amount of tau pulled down by BIN1-SH3-GST following reduction of tau phosphorylation by IC261 treatment. B) Quantification of the amount of tau from vehicle- or IC261-treated neurons pulled down by BIN1-SH3-GST shown as percentage mean control (vehicle). Following Shapiro-Wilk normality testing, data were analysed using a Mann-Whitney test. Data is mean ± S.E.M., n = 3. Full uncut western blots can be found in the supplementary material.

Supplementary Figure 5

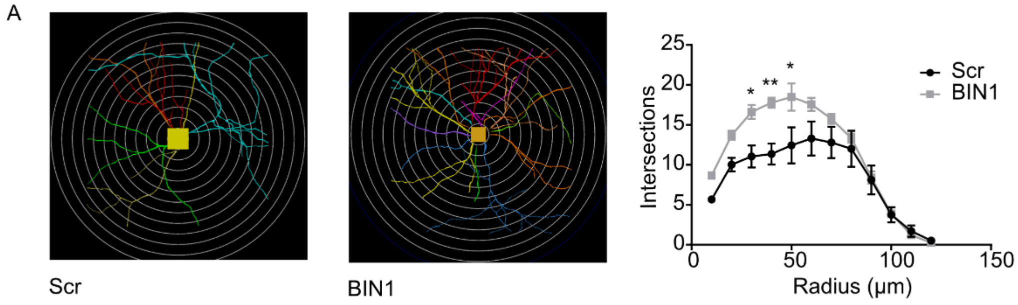

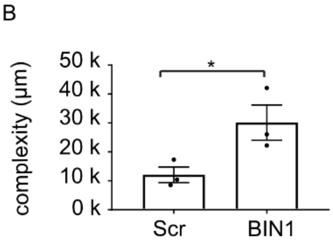

Supplementary Figure 5: BIN1 Knockdown alters neuronal complexity. A) Primary cortical neurons transduced with BIN1 shRNA (BIN1) lentivirus or scrambled control shRNA (Scr) lentivirus at 5 DIV were transfected with a plasmid expressing eGFP and fixed at 23 DIV. Maximum intensity projections were generated from Z-stacks acquired using I-SIM super-resolution imaging. Five to ten different neurons per condition were analysed in each of three separate experiments. The number of intersections at each radius were quantified (graph). Shapiro-Wilk test showed the data were normally distributed, data were analysed by One-way ANOVA with Sidak's multiple comparisons test. B) Quantification of complexity of primary cortical neurons transduced with scrambled control shRNA (Scr) lentivirus or BIN1 shRNA (BIN1) lentivirus. Complexity was calculated as (sum of the terminal orders + number of terminals) * (total dendritic length/number of primary dendrites), where terminals is the number of branch endings, and terminal orders is the number of branches between the terminal and the cell body. Data are mean ± S.E.M. and were analysed using a randomised block 2-way ANOVA. n=3. Graphs show mean ±S.E.M of 3 independent experiments. *$p<0.05$, **$p<0.01$.

Supplementary methods

Proximity Ligation Assays

Proximity ligation assays were performed as described by us previously (Gomez-Suaga *et al.*, 2019) in 23DIV neurons using primary antibodies against BIN1 (ab54764, Abcam, Cambridge, UK) and total tau (Agilent, CA, USA). The actin cytoskeleton was labelled with phalloidin-488 (Life Technologies, CA, USA), and nuclei were stained with 10 μgml^{-1} Hoescht-33342 (Thermo Fischer Scientific). Image stacks covering the whole volume of each cell were acquired using a Nikon Eclipse Ti-E microscope and images were analysed using Fiji. Z-stacks were converted to maximum intensity projections, and the phalloidin signal used to identify cell outlines. The intensity of duo-link signals in each cell was quantified using image J and is expressed as a proportion of cell area.

Analysis of neuronal complexity

For analysis of dendrite structure, neurons at 22 DIV were transfected with an eGFP-N2 plasmid (Clontech, Kyoto, Japan) using lipofectamine 2000 for 24 hours, fixed and the GFP signal imaged using a Nikon Eclipse Ti-2 inverted microscope with Vt-iSIM scan head. 3x3 large image stacks were acquired covering the entire volume of the neuron, with 0.2 µm between each image in the Z plane. Neurolucida^{TM} software (MBF Bioscience, VT, USA) was used to trace neurons and detect, classify and quantify the dendritic spines, and perform Scholl analysis. Neuronal complexity was determined as (sum of terminal orders + number of terminals) * (total dendritic length / number of primary dendrites]), where terminals is the number of branch endings, and terminal order is the number of branches between the terminal and the cell body (Pillai *et al.*, 2012).

References

Garwood CJ, Pooler AM, Atherton J, Hanger DP, Noble W. Astrocytes are important mediators of Abeta-induced neurotoxicity and tau phosphorylation in primary culture. Cell Death Disease 2011; 2:e167.

Gomez-Suaga P, Perez-Nievas BG, Glennon EB, Lau DHW, Paillusson S, Morotz GM, et al. The VAPB-PTPIP51 endoplasmic reticulum-mitochondria tethering proteins are present in neuronal synapses and regulate synaptic activity. Acta Neuropathol Commun 2019; 7:35.

Pillai AG, de Jong D, Kanatsou S, Krugers H, Knapman A, Heinzmann JM, et al. Dendritic morphology of hippocampal and amygdalar neurons in adolescent mice is resilient to genetic differences in stress reactivity. PLoS One 2012; 7:e38971.
