# Supplementary material for: Bridging integrator 1 protein loss in Alzheimer’s disease promotes synaptic tau accumulation and disrupts tau release
Source: Brain Commun. 2020 Feb 14;2(1):fcaa011. doi: 10.1093/braincomms/fcaa011 (PMC7272218; doi:10.1093/braincomms/fcaa011)
Supplement: fcaa011_Supplementary_Data [file fcaa011_supplementary_data.zip › Uncut_blots.pdf]

Uncut western blots

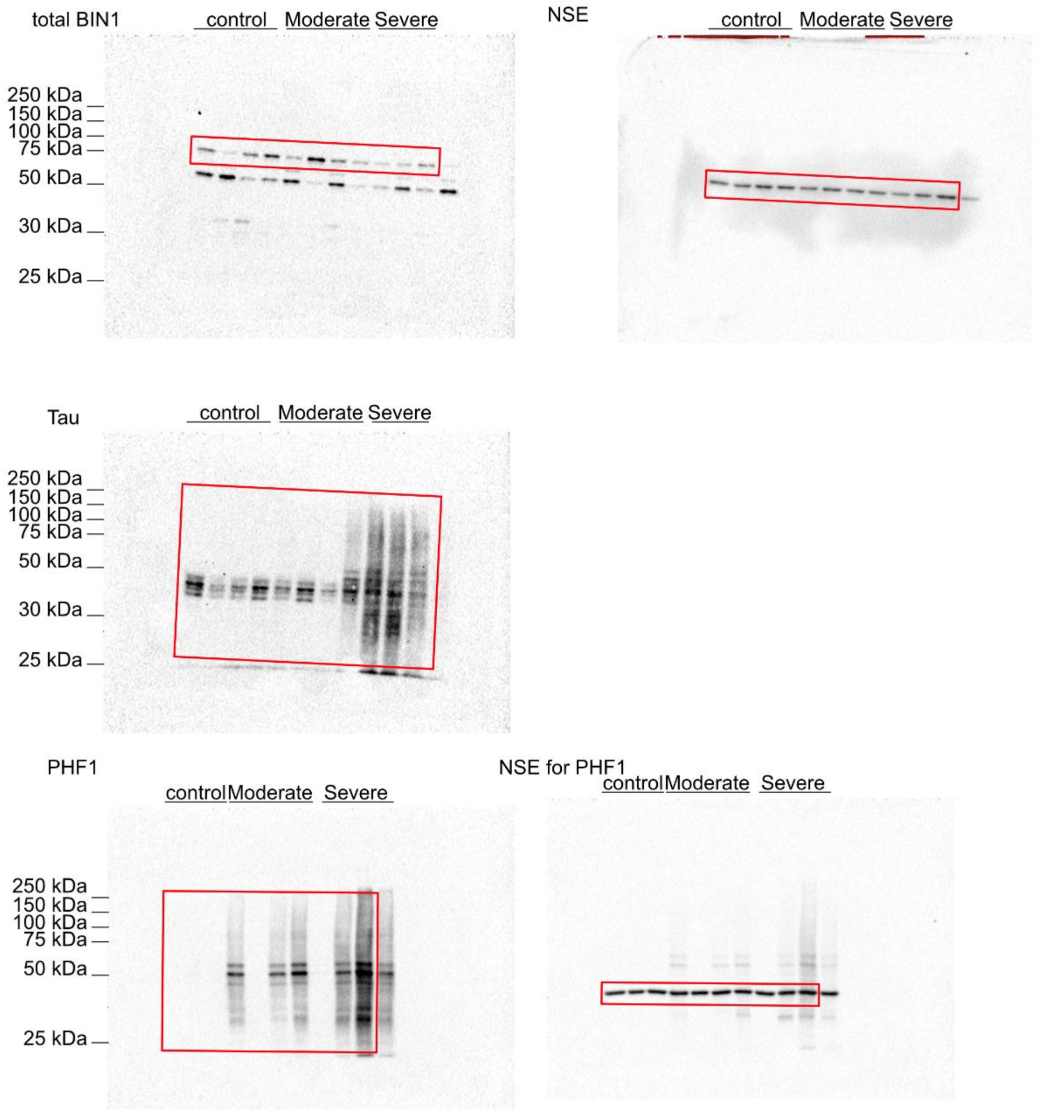

Figure 1A uncut western blots

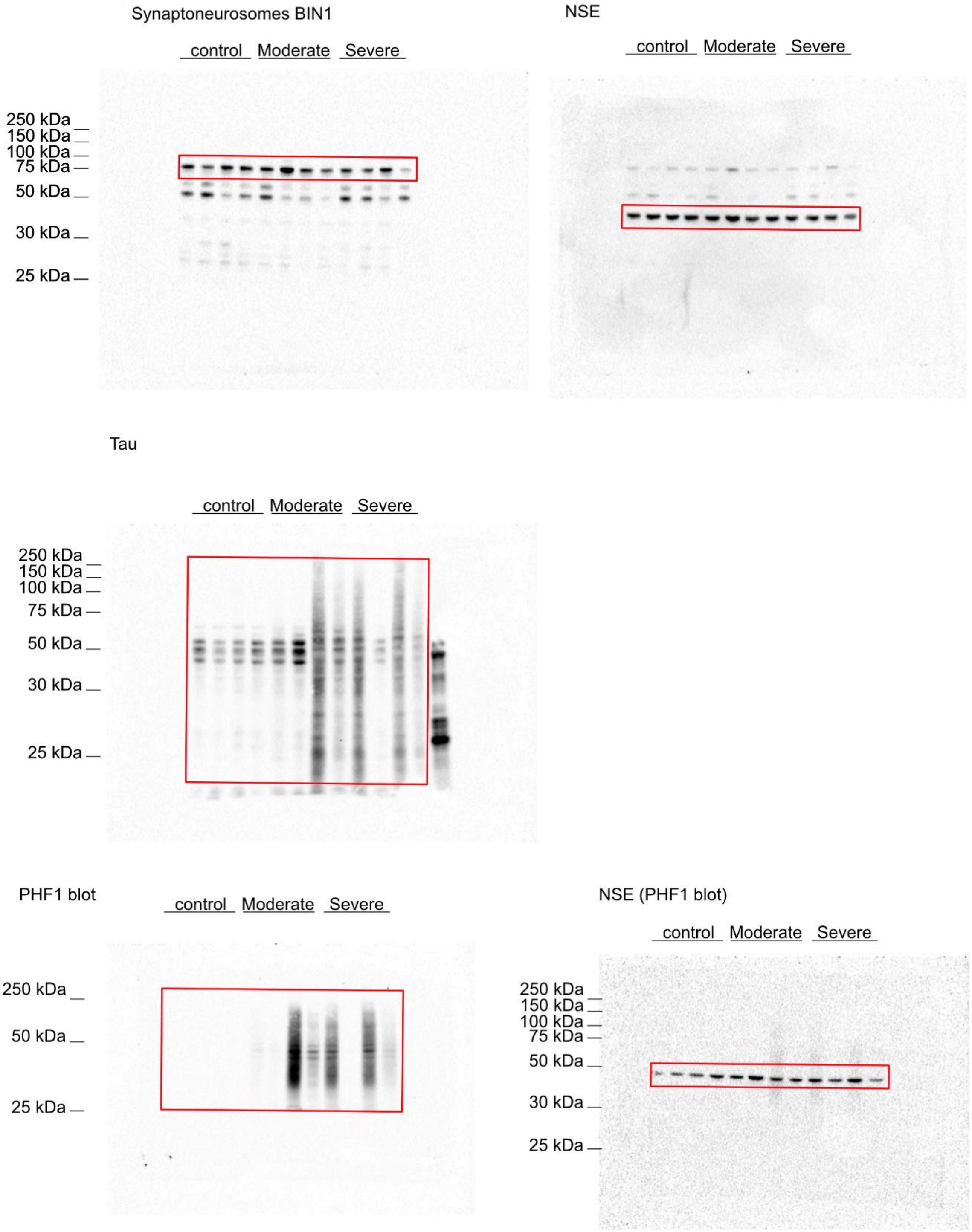

Figure 1E uncut western blots

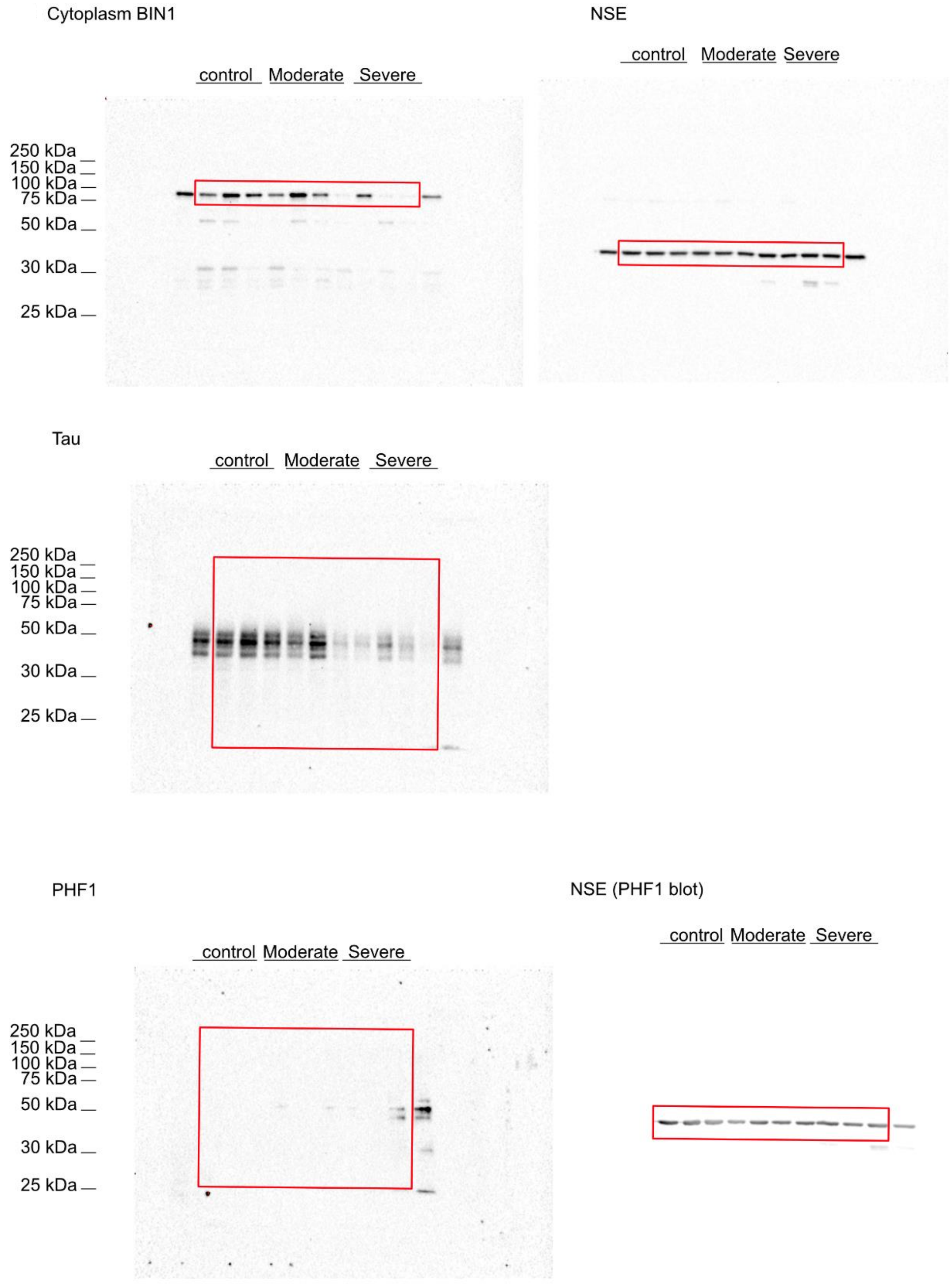

Figure 1I uncut western blots

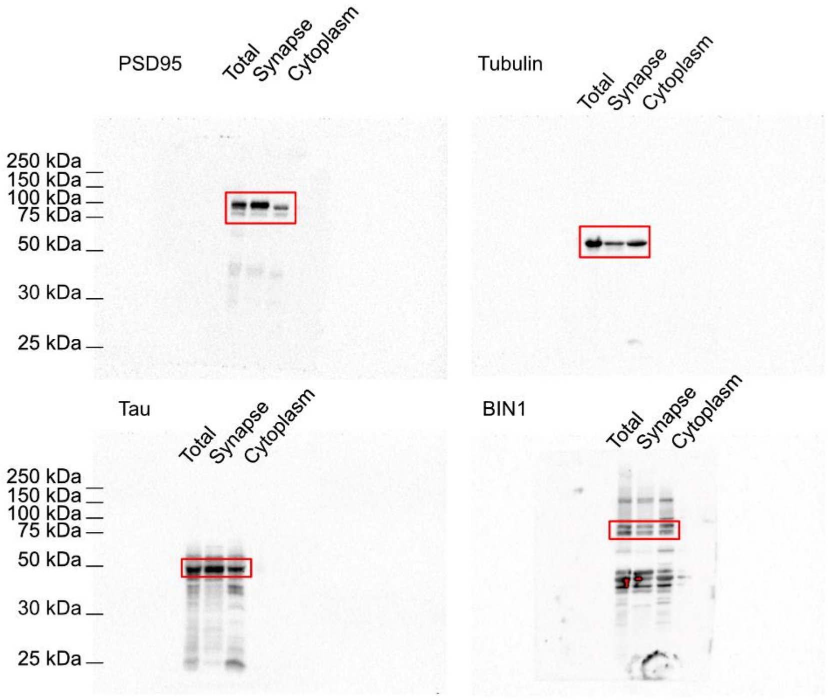

Figure 3A uncut western blots

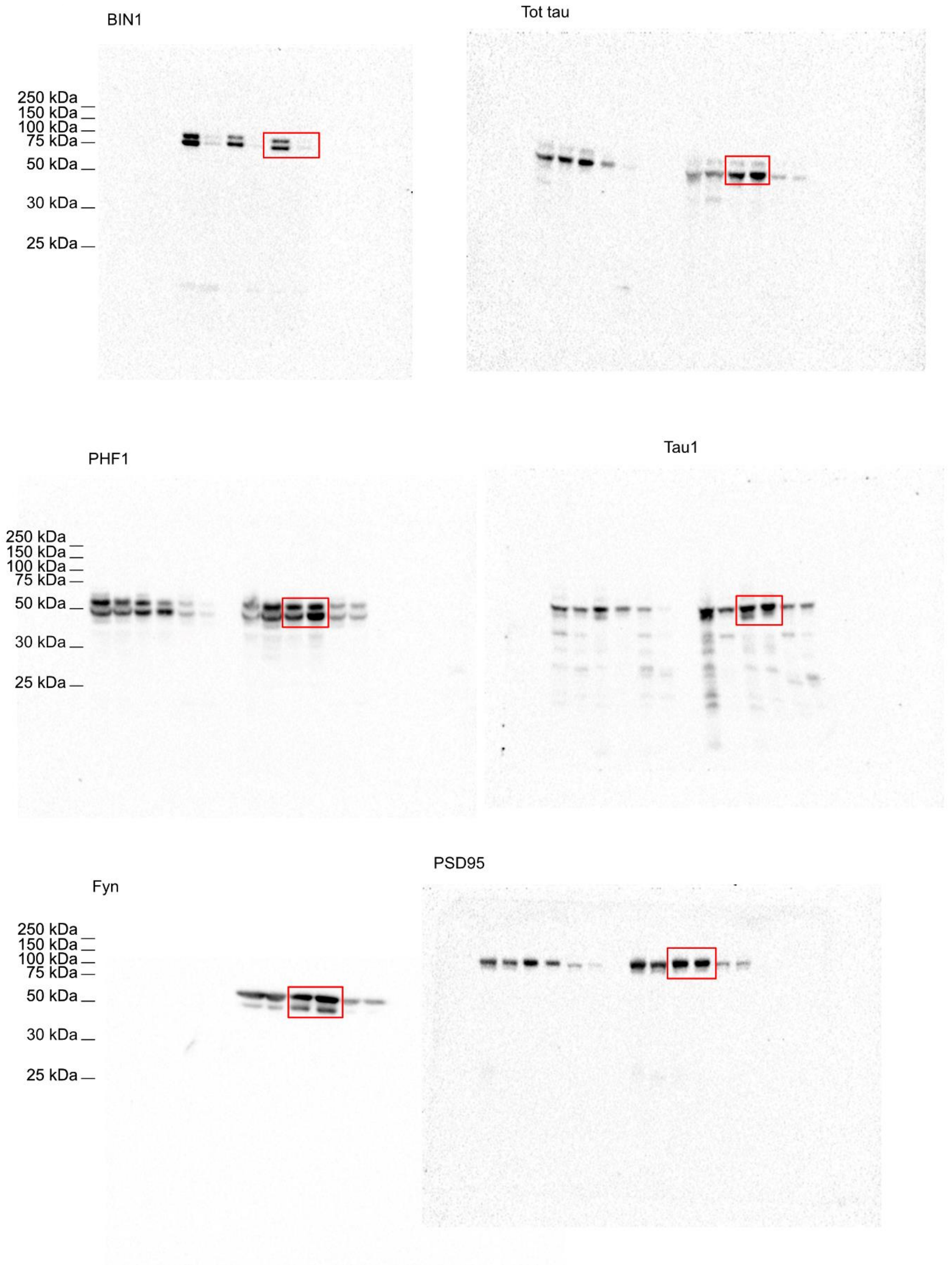

Figure 3D uncut western blots

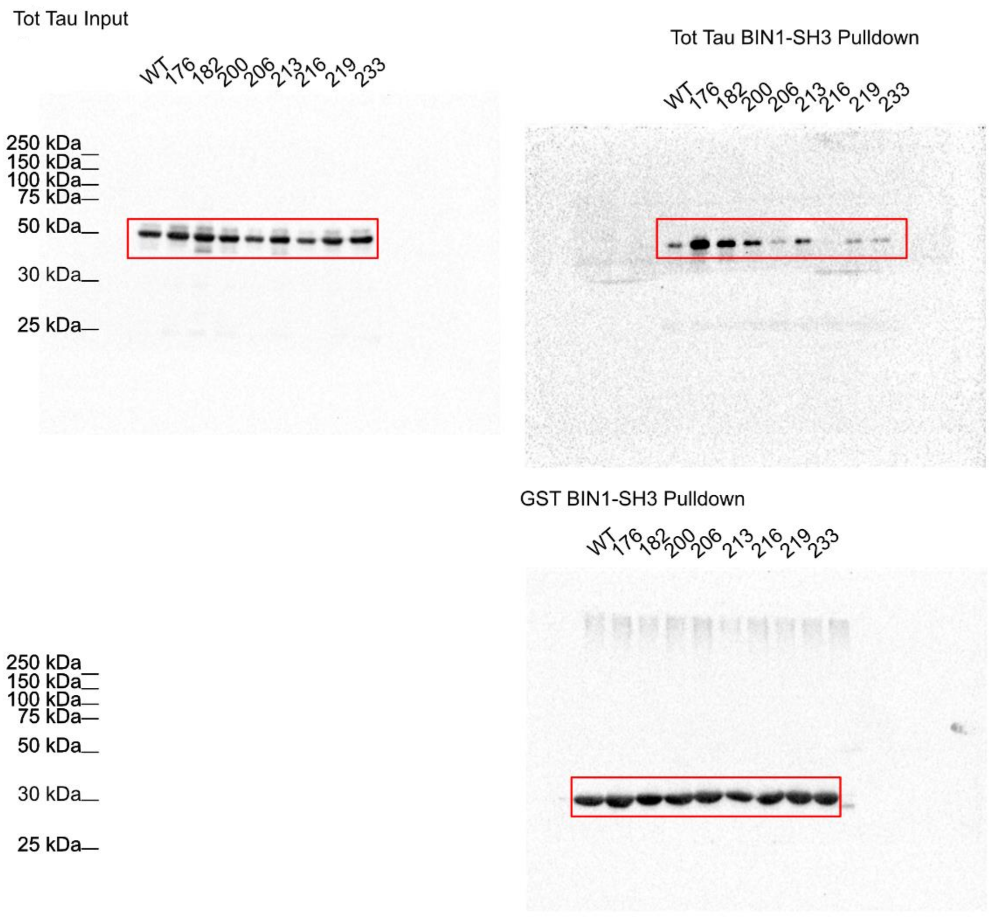

Figure 4A uncut western blots

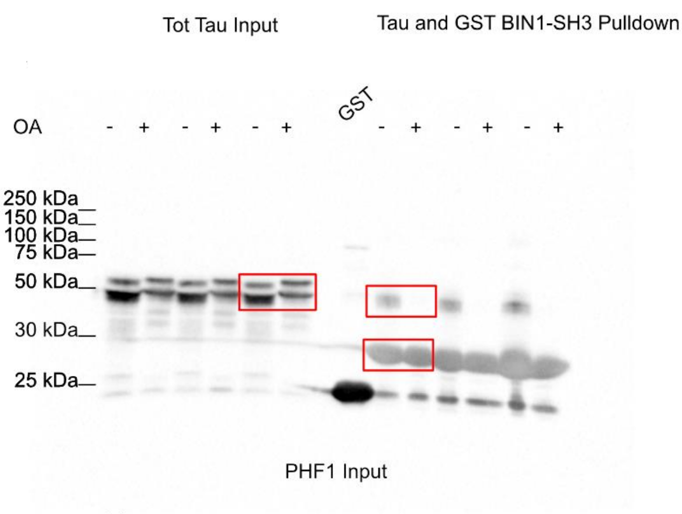

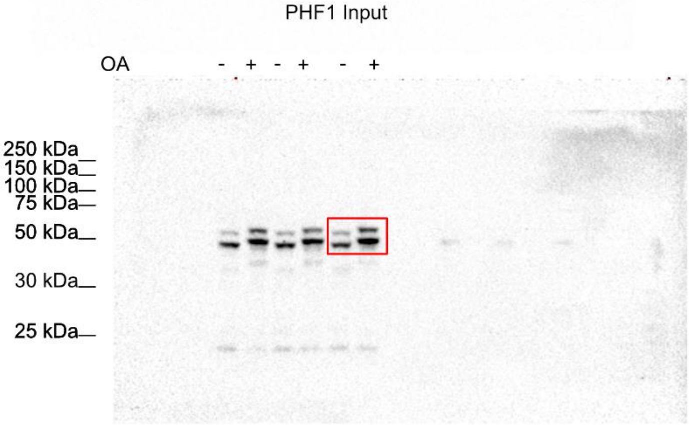

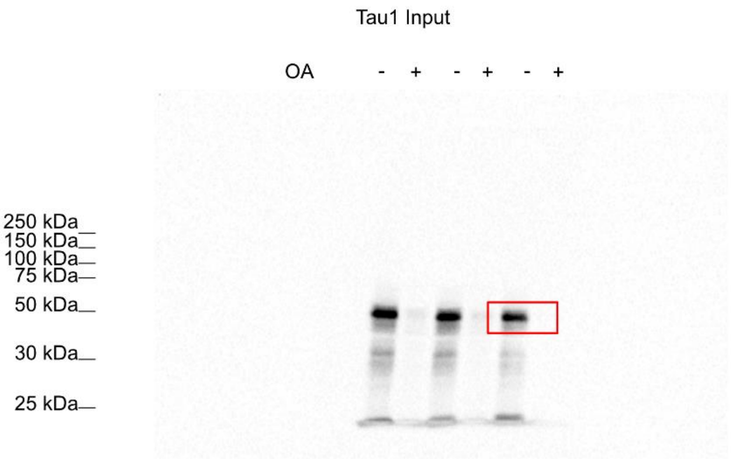

Figure 4C uncut western blots

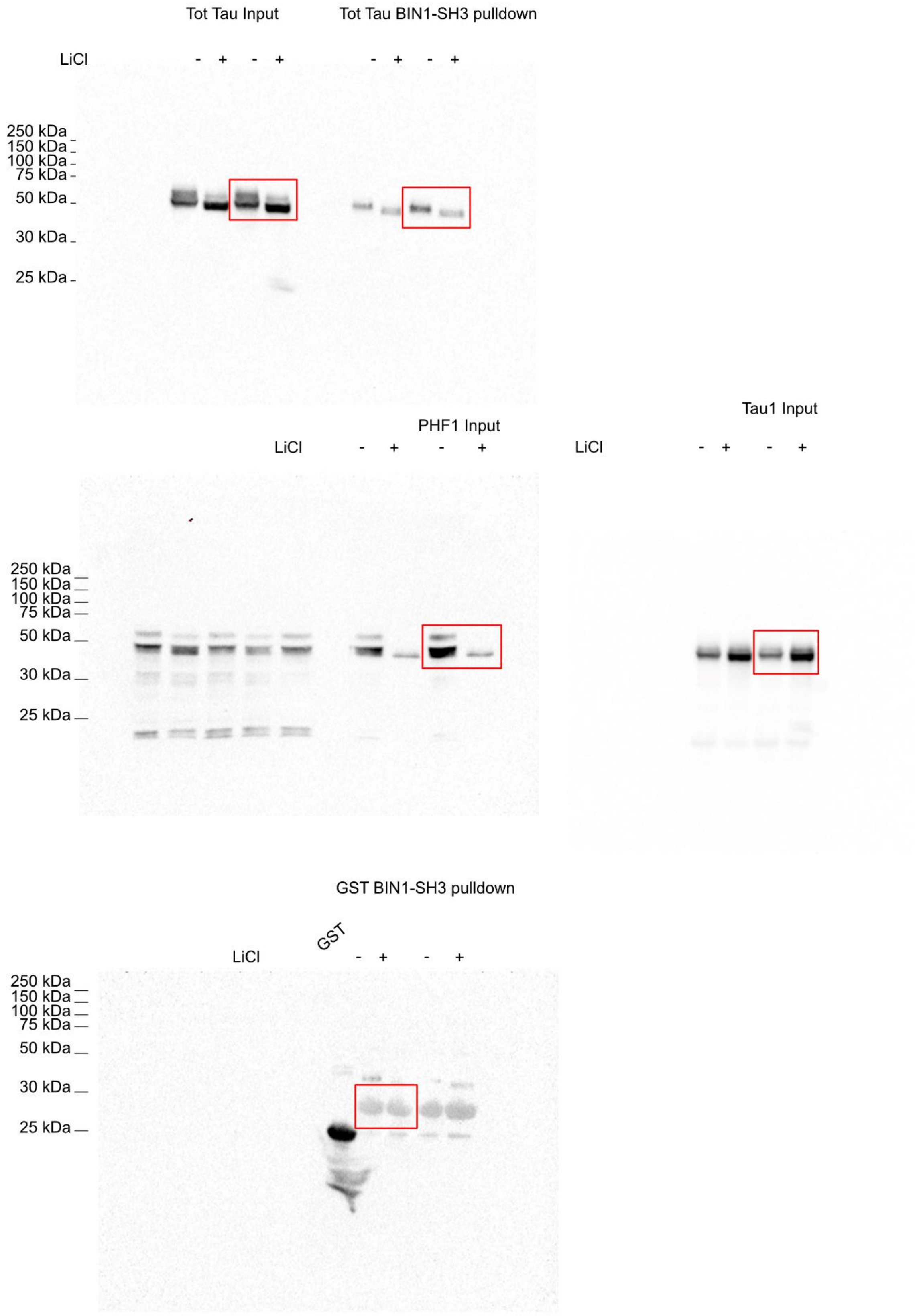

Figure 4E uncut western blots

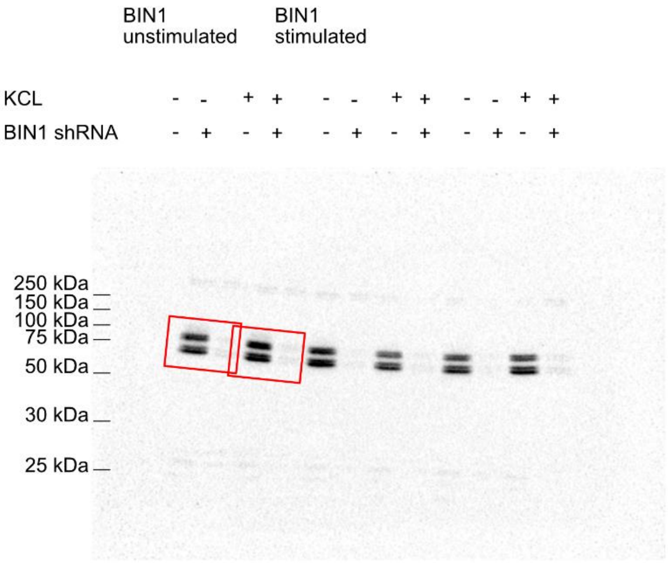

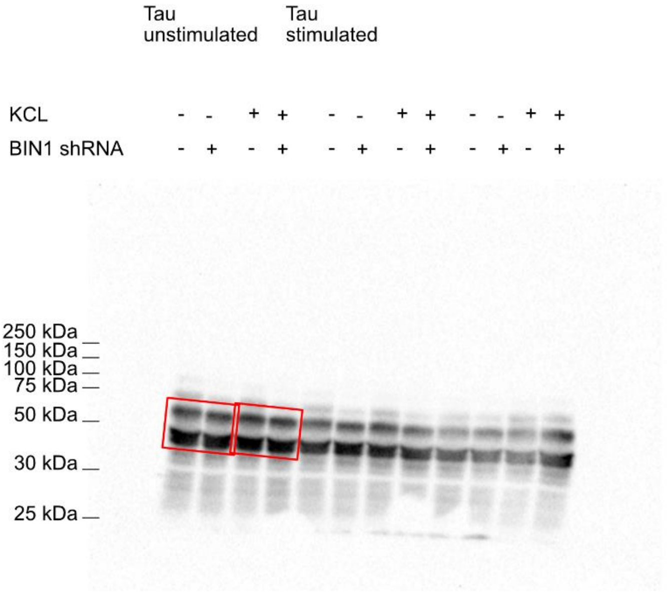

Figure 6 A and D uncut western blots

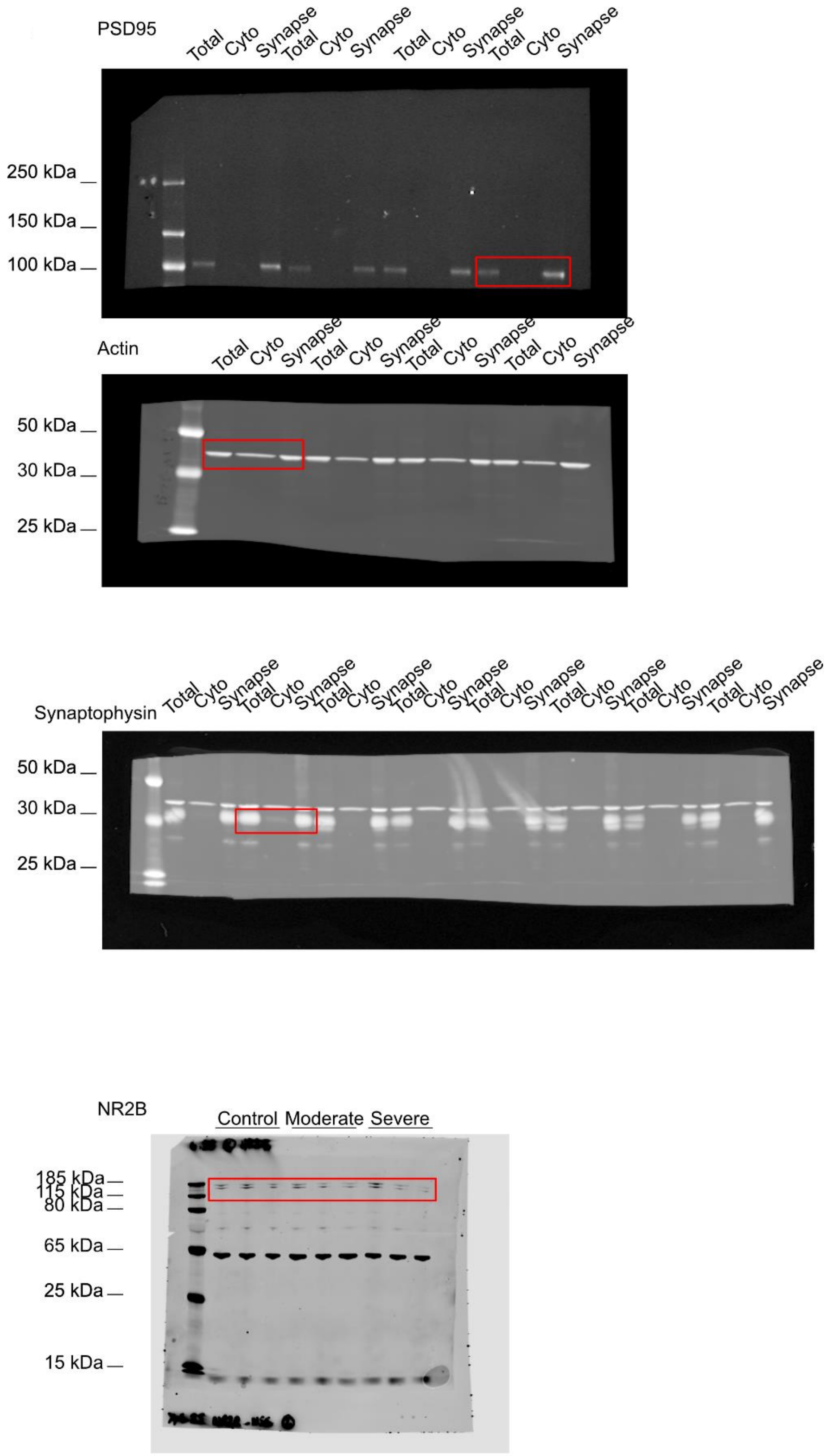

Supplementary Figure 1A and B uncut western blots

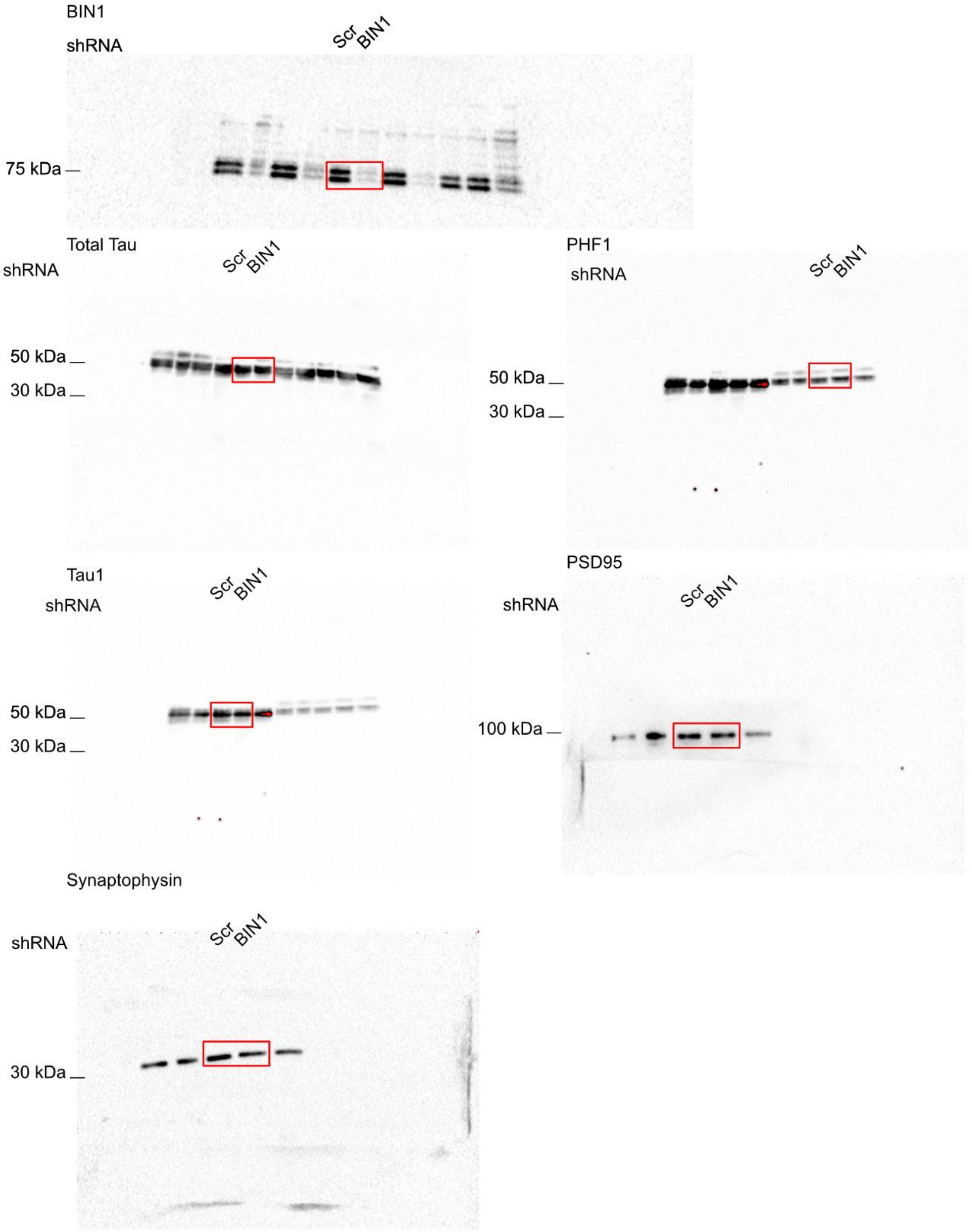

Supplementary Figure 2A uncut western blots

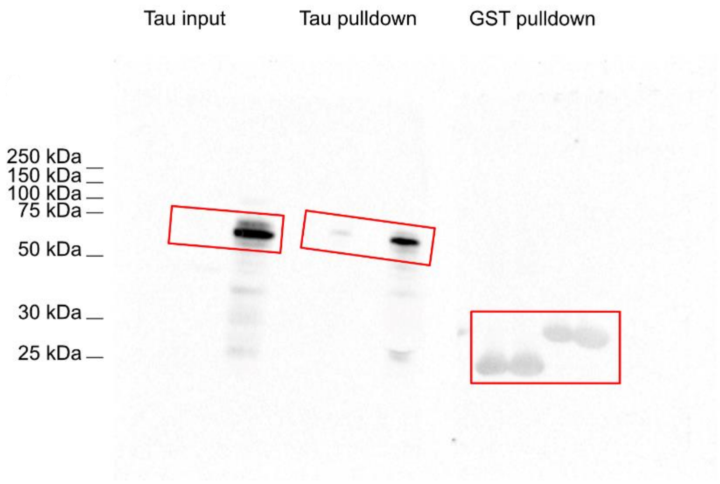

Supplementary Figure 3A uncut western blots

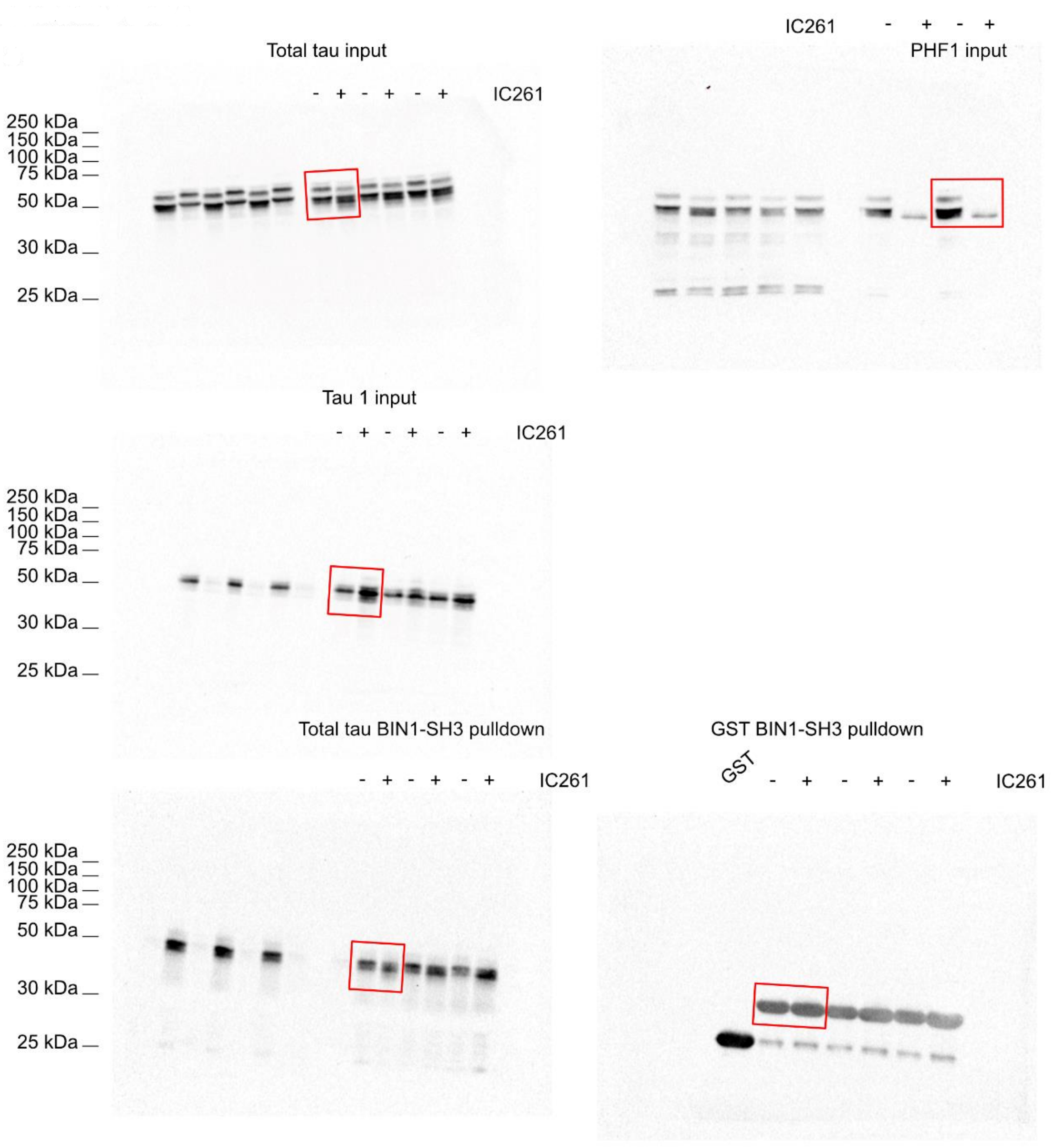

Supplementary Figure 4A uncut western blots
