# Supplementary material for: Bridging integrator 1 protein loss in Alzheimer’s disease promotes synaptic tau accumulation and disrupts tau release
Source: Brain Commun. 2020 Feb 14;2(1):fcaa011. doi: 10.1093/braincomms/fcaa011 (PMC7272218; doi:10.1093/braincomms/fcaa011)
Supplement: fcaa011_Supplementary_Data [file Original_Submission_decision_letter.pdf › Original_Submission_decision_letter.pdf]

BRAINCOM-2019-115 – Original Submission – 30/10/2019

Dear Dr. Glennon,

Thank you for your recent submission BRAINCOM-2019-115 entitled "Loss of BIN1 protein in Alzheimer's disease promotes synaptic accumulation of phosphorylated tau and disrupts tau release" to Brain Communications. Apologies for the delay in processing the paper, we had one reviewer who agreed but never got back to us so we recruited another who kindly got back to us very quickly.

The 2 reviewers were both very positive. They raise have some constructive critiques that I think you could address in a minor revision of the manuscript. Therefore, I invite you to respond to the reviewers' comments and revise your manuscript. The reviewers' comments are included at the bottom of this message.

In addition to the reviewers' comments, please note our editorial comments added below to comply with our requirements for presentation of scientific data at Brain Communications.

To revise your manuscript, click the link below or log into https://mc.manuscriptcentral.com/braincom and enter your Author Centre, where you will find your manuscript title listed under "Manuscripts with Decisions." Under "Actions," click on "Create a Revision." Your manuscript number has been appended to denote a revision.

*** PLEASE NOTE: This is a two-step process. After clicking on the link, you will be directed to a webpage to confirm. ***

https://mc.manuscriptcentral.com/braincom?URL_MASK=5d8febb547c842c6971e8ac42b6d97d5

Please upload a revised manuscript with the changes you have made shown either with "track changes" or in coloured text.

When submitting your revised manuscript, you will be asked to respond to the reviewer(s) comments. Please be as specific as possible in your response to all of the comments.

The editorial team at Brain Communications also aims to increase transparency in scientific publishing and to demonstrate the value that peer review brings to the scientific process. Please therefore consider whether you are willing to have reviewer comments on your paper and your response published as supplementary data with the paper if it is accepted. This is entirely optional and will only be done if both the authors and reviewers agree. When submitting your revision you will have the opportunity to update the answer you entered previously.

IMPORTANT: Your original files are available to you when you upload your revised manuscript. Please delete any redundant files before completing the submission.

Because we are trying to facilitate timely publication of manuscripts submitted to Brain Communications, we would appreciate your revised manuscript as soon as possible. If it is not possible for you to resubmit within 3 months, the option to submit a revision will expire, and we would then consider your revised paper as a new submission. However, if you need a little more time, please get in touch and we can extend this deadline for you.

Once again, thank you for submitting your manuscript to Brain Communications and I look forward to receiving your revision.

Yours sincerely,
Prof. Tara Spires-Jones
Editor, Brain Communications

Reviewer(s)' Comments to Author:

Reviewer: 1
Dr. Christopher Henstridge

Comments to the Author
In this manuscript, Glennon and colleagues have described the effects of BIN1 knockdown on the distribution and release of tau, utilising primary cell culture, molecular biology and the use of human

post-mortem tissue.

They show that BIN1-tau interaction is dependent on proline 216 and that phosphorylation of tau has a negative effect on this interaction. Furthermore, as BIN1 is lost from the cytoplasm, tau levels increase at the synapse and tau release is reduced.

BIN1 is one of the largest genetic risk factors for Alzheimer's, yet we don't fully understand its role, therefore this manuscript provides some useful information for the field. It also confirms/strengthens previous work suggesting an interaction between BIN1 and tau.

However, there are a few minor issues that need to be addressed:
1. This is clearly not the first submission of the manuscript as there are formatting errors throughout, especially with regards to citation style and reference to figures. On numerous occasions the authors refer to the wrong figure and in some cases the figures they refer to don't exist. Also, citation style varies with some papers referred to (ABC et al.) and others just numbered (1). Extensive formatting and proof reading is required.
2. The authors refer to a preprint by Hesse et al. on Biorxiv to describe how they validated their synaptoneurosomes. However, unfortunately the preprint has an error and incorrectly described analysing GluN2A degradation to validate synaptoneurosome integrity, when it should read GluN2B degradation, as described in the original paper by Bayes et al. Molecular Brain 2014. I suggest the authors redo this validation with the correct glutamate receptor subunit. I have contacted the authors of the Hesse et al. paper to inform them of their mistake.
3. In Figure 1 the authors analyse BIN1 levels in groups of "control", "Moderate AD" and "Severe AD" based on Braak staging. They see no significant difference in BIN1 levels. However, I think it may be more insightful to perform a correlation analysis between Braak score and BIN1 levels. Given the link between tau and BIN1 the authors propose, it might be better to look at this across the range of Braak scores rather than arbitrarily lumping them into groups. The authors do this nicely in Figure 2, when comparing protein aggreagation in different cellular compartments.
4. The "data availability" statement is very weak. The authors should mention whether raw data will be available and if so, where will it be stored and how will it be accessed.

Reviewer: 2
Dr. Claire Durrant

Comments to the Author
Summary

Glennon et al. present a well written manuscript identifying a potential link between cytoplasmic levels of BIN1 and mislocalisation of phosphorylated tau to the synapse. They show that low levels of BIN1 in the cytosol correlates with increased presence of phosphorylated tau at the synapse as well as reduced levels of basal and invoked tau release. By combining observations in human post mortem brain with in vitro mechanistic experiments, including identification of a likely tau binding site, this paper will be of interest to researchers interested in the role of tau -binding partners in the development of Alzheimer's disease. I enjoyed reading this manuscript and feel it will be a useful addition to the literature after a few clarifications/ addition of discussion points.

General points

• Could BIN1 lentiviral knockdown in the neurons (from 5 days in vitro) be affecting development rather than modelling an adult degenerative process, especially given the development of rat cortical culture synapses is minimal before 7 days in vitro and continues to mature for several weeks in culture (Ichikawa et al. 1993)? Addition of discussion about this possibility would be of interest as this could affect interpretation of changes to dendritic spine morphology and dendritic complexity for example. Highlighting similarity with knockdown using siRNA at 19 days in vitro (when synapses are more likely to be mature in culture) would strengthen the relevance of the lentiviral data. It is stated in the methods that two methods of knockdown were used but it is not entirely clear which experiments used shRNA lentivirus at 5 days vs siRNA lipofectamine at 19 days (e.g. in figure 3D- the figure image says siRNA but the legend says shRNA. The same is true for Figure 6 and supplementary figure 2.) Could this be clarified?

• As decreasing tau phosphorylation via LiCl did not result in an increased level of tau pulldown in figure 4F (neither did inhibiting casein-kinase 1 activity in supp fig 4), can it be certain that the decrease in pull down with OA treated cultures is entirely due to the increased phosphorylation of tau? (It is somewhat surprising that the binding of BIN1 and tau is maximal under physiological phosphorylation conditions if this interaction is entirely dependent on the phosphorylation status of tau, especially given the extent to which dephosphorylation of tau was achieved with LiCl and casein-kinase inhibition). Could

reversing the effect of OA through addition of lithium (OA +LiCl) or casein-kinase 1 activity demonstrate that the OA is not having effects other than those affecting phosophorylation of tau? Alternatively, could an OA dose-response experiment showing correlation between level of phosphorylation and binding levels be conducted? Increased discussion of this in the manuscript could be helpful.

• The n numbers in supplementary figure 2 (B-D) are not the same as those in figure 3 (E-G) indicating the total homogenates here may not be the same ones from which synaptosomes were generated in figure 3? Showing fractions from the same material (if possible/sufficient material is available from the neuronal samples) would provide greater certainty on whether BIN1 removal alters the location of phosphorylated tau (moving existing pool to the synapse) rather than the likelihood of tau phosphorylation (general increase across entire cell). Whilst the data shown does support the tau mislocalisation hypothesis, I feel this would be strengthened if the data was presented with cytosolic, total and synaptosomal fractions coming from the same source lysate.

Minor alterations/ clarifications

• Figure 1. It would be interesting to see what the levels of PHF1 are in the total and cytosolic fractions as well as the synaptosomal. Does the total or cytosolic ratio of PHF1 to total tau change with AD severity or correlate with BIN1 levels?

• Cytosolic fraction in figure 3A appear to still contain significant quantities of synaptic marker, can the authors comment on the purity of this fraction?

• Figure 3 E-H it is stated these values are normalised to PSD95, but there is no representative blot for PSD95 in fig 3D-please can this be included?

• Specific reference to figure 3H needs to be included in the figure legend.

• Figure 4: The effect of disruption of proline 216 is very strong. It appears 219 has similar effects (bar an outlier)- is this change significant? Do the authors feel that the close proximity of these prolines could mean both affect the binding to some degree?

• Figure 4: It would be good to see the quantification of PHF1 and Tau 1 in fig C and E. It looks by eye that there are clear changes in the ratio of phosphor: non-phosopho tau, but quantification of this would be informative.

• Table 2: It is worth noting that the moderate AD group appear to be significantly (around 10 years) older than either the control or severe group- is there a reason for this? Can the authors comment on this and whether they think this could impact their results? Could those with moderate phenotype actually be more resistant to the disease than those in the severe category who developed a worse pathology at a younger age?

• Supplementary figure 2B-F: please can the authors clarify what housekeeping protein/loading control has been used for normalisation?

• Supplementary figure 2 J needs to be described in the legend.

Editorial Comments:
Please include the following with your revision as they will be required if the paper is accepted:
- Full western-blots (non-cropped) in Supplementary Material
- completed ARRIVE and STROBE checklists
- an abbreviated summary of no more than 50 words
- a graphical abstract.
For more details on these, please see our instructions for authors at:
https://academic.oup.com/braincomms/pages/General_Instructions
