# Supplementary material for: Bridging integrator 1 protein loss in Alzheimer’s disease promotes synaptic tau accumulation and disrupts tau release
Source: Brain Commun. 2020 Feb 14;2(1):fcaa011. doi: 10.1093/braincomms/fcaa011 (PMC7272218; doi:10.1093/braincomms/fcaa011)
Supplement: fcaa011_Supplementary_Data [file Response_to_reviewers_comments_original.pdf › Response_to_reviewers_comments_original.pdf]

Glennon et al. Response to reviews

We thank the reviewers for their mostly positive and constructive reviews. Our responses to their specific comments (in blue) are given below. We indicate where **new data** and/or **discussion** has been added to the manuscript.

Reviewer: 1

1. This is clearly not the first submission of the manuscript as there are formatting errors throughout, especially with regards to citation style and reference to figures. On numerous occasions the authors refer to the wrong figure and in some cases the figures they refer to don't exist. Also, citation style varies with some papers referred to (ABC et al.) and others just numbered (1). Extensive formatting and proof reading is required.

We sincerely apologise for these issues. The formatting has been carefully checked and should all now be correct.

2. The authors refer to a preprint by Hesse et al. on Biorxiv to describe how they validated their synaptoneurosomes. However, unfortunately the preprint has an error and incorrectly described analysing GluN2A degradation to validate synaptoneurosome integrity, when it should read GluN2B degradation, as described in the original paper by Bayes et al. Molecular Brain 2014. I suggest the authors redo this validation with the correct glutamate receptor subunit. I have contacted the authors of the Hesse et al. paper to inform them of their mistake.

We thank the reviewer for pointing out this mistake. We have now performed these experiments using an antibody against the NR2B subunit of the NMDA receptor (**revised Supplementary Figure 1B, C**). The results of this analysis indicate that although there is some loss of synapse integrity in post-mortem tissues, most samples showed a ratio of approx. 1 in agreement with Bayes et al. (2014). There were no differences in this measure of synaptic integrity between control, moderate and severe AD groups. Therefore, we are confident that the protein differences we report in moderate and severe AD groups relative to controls have not occurred as a result of synaptic breakdown in these groups of samples. We discuss this finding on **Page 12** and we have updated the Methods section with the details of this antibody (**Page 11**).

3. In Figure 1 the authors analyse BIN1 levels in groups of "control", "Moderate AD" and "Severe AD" based on Braak staging. They see no significant difference in BIN1 levels. However, I think it may be more insightful to perform a correlation analysis between Braak score and BIN1 levels. Given the link between tau and BIN1 the authors propose, it might be better to look at this across the range of Braak scores rather than arbitrarily lumping them into groups. The authors do this nicely in Figure 2, when comparing protein aggregation in different cellular compartments.

I am afraid that we disagree with the Reviewer on this point. There is a large degree of variability in phosphorylated and aggregated tau amounts within Braak stages in different brain regions (e.g. Abner et al., 2011; Walker et al., 2017 and our Figure 1), which means that analyses based solely on Braak stage is not informative, and this will also significantly reducing the power of our current analyses. As we discuss in the paper, we hypothesise that there is a redistribution of intracellular soluble phosphorylated tau species as a consequence of lowered BIN1 amounts in AD, and therefore it is most appropriate to examine the correlation between BIN1 and phosphorylated tau amounts and not the NFT-like tau indicated by Braak stage. In support of this, we see no correlation between total tau amounts (which includes all

forms of tau and not just highly phosphorylated species) and BIN1 in either the total lysate and synaptoneurosome fraction. **We now show this data as Supplementary Figs 1G and H**.

4. The "data availability" statement is very weak. The authors should mention whether raw data will be available and if so, where will it be stored and how will it be accessed.

Our data availability statement has now been amended (**Pages 11-12**). Summary data are provided as a **new Supplementary file**. All raw experimental data is generated in electronic format and is backed up immediately after generation and analysis onto the King's College London servers which provide secure, long-term storage for research data.

Reviewer: 2

1. Could BIN1 lentiviral knockdown in the neurons (from 5 days in vitro) be affecting development rather than modelling an adult degenerative process, especially given the development of rat cortical culture synapses is minimal before 7 days in vitro and continues to mature for several weeks in culture (Ichikawa et al. 1993)? Addition of discussion about this possibility would be of interest as this could affect interpretation of changes to dendritic spine morphology and dendritic complexity for example. Highlighting similarity with knockdown using siRNA at 19 days in vitro (when synapses are more likely to be mature in culture) would strengthen the relevance of the lentiviral data. It is stated in the methods that two methods of knockdown were used but it is not entirely clear which experiments used shRNA lentivirus at 5 days vs siRNA lipofectamine at 19 days (e.g. in figure 3D- the figure image says siRNA but the legend says shRNA. The same is true for Figure 6 and supplementary figure 2.) Could this be clarified?

We thank the reviewer for this comment. We include discussion on **Page 20** that BIN1 is not expressed until DIV 7-14 in primary cultures (Sartori et al., 2019) and therefore although we cannot completely rule out any effect of knocking down BIN1 in immature neurons, it is expected that there is no effect of BIN1 knockdown until neurons have matured.

We apologise and have corrected **Figures 3 and 6** legends to correct these errors. shRNA was used in both of these sets of experiments. siRNA was only used for the experiment shown in Supplementary Figure 2G.

2. **As decreasing** tau phosphorylation via LiCl did not result in an increased level of tau pulldown in figure 4F (neither did inhibiting casein-kinase 1 activity in supp fig 4), can it be certain that the decrease in pull down with OA treated cultures is entirely due to the increased phosphorylation of tau? (It is somewhat surprising that the binding of BIN1 and tau is maximal under physiological phosphorylation conditions if this interaction is entirely dependent on the phosphorylation status of tau, especially given the extent to which dephosphorylation of tau was achieved with LiCl and casein-kinase inhibition). Could reversing the effect of OA through addition of lithium (OA +LiCl) or casein-kinase 1 activity demonstrate that the OA is not having effects other than those affecting phosophorylation of tau? Alternatively, could an OA dose-response experiment showing correlation between level of phosphorylation and binding levels be conducted? Increased discussion of this in the manuscript could be helpful.

We agree with the Reviewer that this point is worthy of further discussion, which we have now added to **Page 18**. We have confidence in our results with OA since our results from postmortem AD brain show that in conditions when tau is highly phosphorylated, tau is lost

from the cytoplasm and accumulates in synaptic fractions. Since we saw no increase in synaptic BIN1 this clearly indicates a disrupted association between BIN1 and tau since they are no longer in the same cellular compartments in agreement with our cell culture findings. Secondly, it is accepted that increased phosphorylation of tau disrupts its interactions with SH3 domain-containing proteins such as BIN1, and our results are entirely consistent with this. That said, we appreciate the comment that it is somewhat surprising that the binding of tau with BIN1 is not increased from baseline when tau is dephosphorylated since a degree of tau phosphorylation is expected in basal cell culture conditions. We discuss this point on **Page 18** and suggest that future dose-response experiments or experiments using tau phosphomimics could be used to further examine the effects of tau phosphorylation on its interactions with BIN1 in the future.

3. The n numbers in supplementary figure 2 (B-D) are not the same as those in figure 3 (E-G) indicating the total homogenates here may not be the same ones from which synaptosomes were generated in figure 3? Showing fractions from the same material (if possible/sufficient material is available from the neuronal samples) would provide greater certainty on whether BIN1 removal alters the location of phosphorylated tau (moving existing pool to the synapse) rather than the likelihood of tau phosphorylation (general increase across entire cell). Whilst the data shown does support the tau mislocalisation hypothesis, I feel this would be strengthened if the data was presented with cytosolic, total and synaptosomal fractions coming from the same source lysate.

The reviewer is correct that, while it would have been ideal to use total lysates and synaptosomes from the same neurons, it was not possible to perform all experiments in this way due to the large amount of starting material required for the synaptosome preps. We show below a representative blot from a total lysate generated as part of one synaptosome prep which shows no overall alterations in total or phosphorylated tau amounts and is very comparable to the total lysate data that we show in Supplementary Figure 2. The cells from which the total cell lysates samples were generated were treated identically to the cells which were fractionated, and we are confident that alteration in BIN1 expression does not alter total tau levels, but allows a pool of phosphorylated tau to mislocalise to synapses.

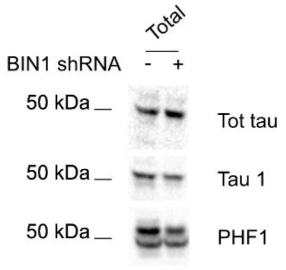

4. Figure 1. It would be interesting to see what the levels of PHF1 are in the total and cytosolic fractions as well as the synaptosomal. Does the total or cytosolic ratio of PHF1 to total tau change with AD severity or correlate with BIN1 levels?

We thank the Reviewer for this comment. We have added PHF1 blots and quantification to **revised Fig 1** and we discuss the results on **Pages 12-13**. Increases in the amount of phosphorylated tau are found with increasing Braak stage in total lysate fractions, similar to findings reported by us and others previously (Perez-Nievas et al., 2013; Kurbatskaya et al., 2016). There is also an increase in cytoplasmic PHF1 predominantly in Braak stage V-VI

tissues. As previously reported (Perez-Nievas et al., 2013), this is much less abundant than detected in the total or synaptoneurosome fraction when equivalent protein amounts from each fraction are studied. Importantly, while we find a strong positive correlation between total tau amounts and BIN1 in the cytoplasm, there is a negative correlation between phosphorylated tau and BIN1 in this fraction. This supports our *in vitro* data that phosphorylated tau loses its ability to bind BIN1. The results of all correlation analyses are shown in **revised Figure 2 and Supplementary Figure 1G-J**).

5. Cytosolic fraction in figure 3A appear to still contain significant quantities of synaptic marker, can the authors comment on the purity of this fraction?

Due to lower quantities of material available we used a different "synaptosome" fractionation protocol for cultured neurons than the synaptoneurosome fractionation used for human brain tissues. This synaptosome protocol generates enriched rather than pure synaptic fractions. This was already stated in the Methods section (Page 8, now Page 9). However, we have further clarified this in the manuscript **Page 14** and **Figure 3 legend**.

6. Figure 3 E-H it is stated these values are normalised to PSD95, but there is no representative blot for PSD95 in fig 3D-please can this be included?

We apologise for this oversight. A representative PSD95 blot is now shown in **Revised Fig 3**.

7. Specific reference to figure 3H needs to be included in the figure legend.

We discuss Figure 3H in the manuscript on **Page 14**. We thought it was important to assess Fyn levels since both Fyn and BIN1 interact predominantly with P216 in tau (Usardi et al., 2011; Lau et al., 2016) and it was possible that competition for binding between BIN1 and Fyn may have had consequences for Fyn localisation.

8. Figure 4: The effect of disruption of proline 216 is very strong. It appears 219 has similar effects (bar an outlier)- is this change significant? Do the authors feel that the close proximity of these prolines could mean both affect the binding to some degree?

We thank the Reviewer for this comment. Our analysis did not detect a significant decrease in the interaction between the BIN1-SH3 domain and tau-P219A, even with the outlier removed. However, it does appear that P219A may have some effect on tau-BIN1 binding and other have reported that proline 219 of tau is in direct contact with residues of BIN1 (Lasorsa et al. 2018). We discuss this point on **Page 18**.

9. Figure 4: It would be good to see the quantification of PHF1 and Tau 1 in fig C and E. It looks by eye that there are clear changes in the ratio of phosphor: non-phosopho tau, but quantification of this would be informative.

We agree with the reviewer that there are clear changes in the amounts of phospho- and non-phospho tau following treatment with LiCl and OA in Fig 4, and the effect of these treatments on tau phosphorylation is widely reported in the literature (e.g. Pooler et al. 2012; Van Dolah and Ramsdell 1992; Stambolic, Ruel, and Woodgett 1996). We have not quantified these findings since the blots are so clear and because of in some cases there is no signal to quantify (e.g. Tau 1 in OA-treated cells, and to a large extent PHF1 in LiCl-treated neurons).

10. Table 2: It is worth noting that the moderate AD group appear to be significantly (around 10 years) older than either the control or severe group- is there a reason for this? Can the authors comment on this and whether they think this could impact their results? Could those with moderate phenotype actually be more resistant to the disease than those in the severe category who developed a worse pathology at a younger age?

This is an interesting point. We requested age-matched controls from the Brain Bank, so can only assume that they have a bias towards older cases at Braak stages III-IV. We have added some discussion about possible resilience in some of the moderate cases to **Page 19**. It should be noted that there are several cases within the moderate group of similar or younger ages than those in the severe group. Moreover, our analysis of the relationship between phosphorylated tau and BIN1 distributions was conducted independently of Braak stage (see response to Rev 1, point 3) and therefore we do not consider this to confound our findings.

11. Supplementary figure 2B-F: please can the authors clarify what housekeeping protein/loading control has been used for normalisation?

These data were normalised to total protein levels. This information has been added to materials and methods (**Page 11**) and **Supplementary Figure 2 legend**.

12. Supplementary figure 2 J needs to be described in the legend.

We apologise that the description of Supplementary Figure 2J was mis-labelled in the Figure legend. This has now been corrected.

Editor comments

Please include the following with your revision as they will be required if the paper is accepted:
- Full western-blots (non-cropped) in Supplementary Material
- completed ARRIVE and STROBE checklists
- an abbreviated summary of no more than 50 words
- a graphical abstract.

We include these files with the submitted revision.

References

Abner EL, Kryscio RJ, Schmitt FA, Santacruz KS, Jicha GA, Lin Y, Neltner JM, Smith CD, Van Eldik LJ, Nelson PT. (2011). "End-stage" neurofibrillary tangle pathology in preclinical Alzheimer's disease: fact or fiction? J Alzheimers Dis. 25(3):445-53.

Bayés À, Collins MO, Galtrey CM, Simonnet C, Roy M, Croning MD, Gou G, van de Lagemaat LN, Milward D, Whittle IR, Smith C, Choudhary JS, Grant SG. (2014). Human post-mortem synapse proteome integrity screening for proteomic studies of postsynaptic complexes. Mol Brain. 7:88.

Lasorsa A, Malki I, Cantrelle FX, Merzougui H, Boll E, Lambert JC, Landrieu I. (2018). Structural Basis of Tau Interaction With BIN1 and Regulation by Tau Phosphorylation. Front Mol Neurosci. 11:421.

Lau DH, Hogseth M, Phillips EC, O'Neill MJ, Pooler AM, Noble W, et al. (2016). Critical residues involved in tau binding to fyn: implications for tau phosphorylation in Alzheimer's disease. Acta Neuropathol Commun. 4: 49.

Perez-Nievas BG, Stein TD, Tai HC, Dols-Icardo O, Scotton TC, Barroeta-Espar I, et al. (2013). Dissecting phenotypic traits linked to human resilience to Alzheimer's pathology. Brain. 136: 2510-26.

Pooler AM, Usardi A, Evans CJ, Philpott KL, Noble W, Hanger DP. (2012). Dynamic association of tau with neuronal membranes is regulated by phosphorylation. Neurobiol Aging. 33(2):431.e27-38.

Sartori M, Mendes T, Desai S, Lasorsa A, Herledan A, Malmanche N, Mäkinen P, Marttinen M, Malki I, Chapuis J, Flaig A, Vreulx AC, Ciancia M, Amouyel P, Leroux F, Déprez B, Cantrelle FX, Maréchal D, Pradier L, Hiltunen M, Landrieu I, Kilinc D, Herault Y, Laporte J, Lambert JC. (2019). BIN1 recovers tauopathy-induced long-term memory deficits in mice and interacts with Tau through Thr348 phosphorylation. Acta Neuropathol. 138(4):631-652.

Stambolic V, Ruel L, Woodgett JR. (1996). Lithium inhibits glycogen synthase kinase-3 activity and mimics wingless signalling in intact cells. Curr Biol, 6: 1664-8.

Usardi A, Pooler AM, Seereeram A, Reynolds CH, Derkinderen P, Anderton B, et al. (2011). Tyrosine phosphorylation of tau regulates its interactions with Fyn SH2 domains, but not SH3 domains, altering the cellular localization of tau. FEBS J. 278: 2927-37.

Van Dolah FM, Ramsdell JS. (1992). Okadaic acid inhibits a protein phosphatase activity involved in formation of the mitotic spindle of GH4 rat pituitary cells, J Cell Physiol, 152: 190-8.

Walker L, McAleese KE, Johnson M, Khundakar AA, Erskine D, Thomas AJ, McKeith IG, Attems J. (2017). Quantitative neuropathology: an update on automated methodologies and implications for large scale cohorts. J Neural Transm. 124(6):671-683.
