# Supplementary material for: Bridging integrator 1 protein loss in Alzheimer’s disease promotes synaptic tau accumulation and disrupts tau release
Source: Brain Commun. 2020 Feb 14;2(1):fcaa011. doi: 10.1093/braincomms/fcaa011 (PMC7272218; doi:10.1093/braincomms/fcaa011)
Supplement: fcaa011_Supplementary_Data [file Revision1_decision_letter.pdf › Revision1_decision_letter.pdf]

BRAINCOM-2019-115 Revision 1 8/1/2020

Dear Dr. Glennon,

Thank you for your careful revisions of your paper after peer review. It is a pleasure to accept your manuscript entitled "Loss of BIN1 protein in Alzheimer's disease promotes synaptic accumulation of phosphorylated tau and disrupts tau release" for publication in Brain Communications. Any comments made by the reviewer(s) are included below.

The next step is for your paper to go through "First Look". We will contact you again shortly if any final files or formatting changes are required for publication or if we have any queries.

If no changes are needed, your submission will be transferred to our Production Department and you will hear from the Production Editor in due course.

Thank you very much for your contribution. On behalf of the Editors of Brain Communications, we look forward to your future contributions to the Journal.

Yours sincerely,
Professor Tara Spires-Jones
Editor
Brain Communications
